# Five thousand years of inequality in the Carpathian Basin

**DOI:** 10.1126/sciadv.adu0323

**Published:** 2025-08-06

**Authors:** Paul R. Duffy, Fynn Wilkes, Henry Skorna, Martin Furholt, Cait Dickie, Kata Furholt, Giacomo Bilotti, Johannes Müller, Gary M. Feinman

**Affiliations:** ^1^ Cluster of Excellence ROOTS, Kiel University, Leibnizstrasse 3, Kiel D-24118, Germany.; ^2^ Institute of Pre- and Protohistory, Kiel University, Johanna-Mestorf Strasse 2-6, Kiel D-24118, Germany.; ^3^ CRC 1266, Kiel University, Johanna-Mestorf Strasse 2-6, Kiel D-24118, Germany.; ^4^ School of Culture and Society - Social Resilience Lab, Aarhus University, Jens Chr. Skous Vej 4, Aarhus C 8000, Denmark.; ^5^ Negaunee Integrative Research Center, Field Museum of Natural History, Chicago, IL 60605, USA.; ^6^ Department of Anthropology, University of Illinois, Chicago, IL 60607, USA.; ^7^ Department of Anthropology, Northwestern University, Evanston, IL 60208, USA.

## Abstract

The emergence of sedentary farming economies, especially in contexts intensified by plow agriculture, has been argued to underpin marked increases in economic inequality and its intergenerational transmission across Eurasia. To assess this presumed causal relationship, we examine relational (burials) and material (house sizes) inequalities in the Carpathian Basin, a large region in central Europe, from the time the first farmers arrived in southeastern Europe through the next five millennia to the Bronze Age. We find that although farming did increase the potentials for both relational and material inequalities, the potential was rarely reached and then only for short durations. We identify a series of leveling mechanisms varying over time, including the removal of material wealth from circulation through the placement in graves, community fission, and investments of surplus labor in infrastructural investments. In the Carpathian Basin, only after at least 5000 years were the intergenerational potentials of material wealth transmissions more broadly realized.

## Teaser

The introduction of farming, including plow agriculture, did not lead to greater or sustained inequalities in southeastern Europe.

## INTRODUCTION

**Fig. 1.**
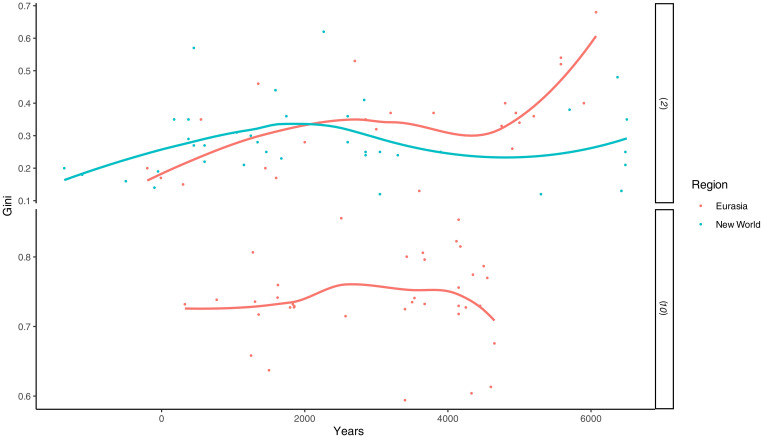
Changes in inequality found in two recent studies. (**Top**) Inequality values in house sizes since the local onset of agriculture; value 0 on the *x* axis [data from (*2*, *52*)]. (**Bottom**) Inequality values from cemetery contexts in the Carpathian Basin since the local onset of agriculture [data from (*10*)].

Over the past decade, a wave of research has explored the deep historical roots of inequality. Although inequality has different dimensions, researchers use the Gini coefficient as a key metric to probe wealth disparities, as they relate to house sizes in the Americas and Western Eurasia. The Gini coefficient uses income data or material wealth to produce a value between 0 and 1, which represents the degree to which resources in a society are concentrated in a few hands. Focused principally on Eurasia, one recent hypothesis links the onset of plow (or land-limited) agriculture to growing disparities in intra-settlement house sizes and so wealth (*1*–*3*). In one dataset, inequalities in Eurasia stabilized ~2500 years after farming was introduced and then ratchet up in a way that they do not in the Americas [(*2*); Fig. 1, top]. Researchers argue that once plow agriculture became entrenched, a “warrior elite” allowed expanded territorial control and inequalities blossomed. These foundational investigations provide support for the long-entrenched notion (*4*) that, once advanced agriculture was established, surpluses were regularly generated, private property became institutionalized (*5*, *6*), and while regionally variable, there was an inevitable ratcheting up of economic inequality.

However, empirical measures of inequality from later European prehistory are greatly underrepresented in these narratives. House size Gini values from fifth-millennium Ukraine indicate there were agricultural strategies and social mechanisms that reduced inequalities at a time when community sizes exploded (*7*, *8*). Although inequalities in mortuary customs become more pronounced in the early second millennium BCE, these arguably constitute differences in aspirational power and variable densities of interpersonal social networks rather than demonstrable hierarchies (*9*). Moreover, mortuary findings from the Carpathian Basin illustrate a waxing and waning of inequalities from the fifth to second millennium BCE, such that the mean Gini value does not increase significantly over time [(*10*); Fig. 1, bottom]. These studies present clear contrasts to the predominant narrative outlined above and beg close consideration before the advent of farming and Malthusian demographic growth are seen as the inevitable prime movers of economic inequalities.

Here, we complement mortuary Gini data with variation in house sizes from the Carpathian Basin in central Europe to compare changes in different modes of inequality (*11*) over time. In addition to contrasting Gini measures, however, we seek to understand the leveling mechanisms that dampened inequalities across long temporal sequences. To this end, we use the framework, empirical foundation, and expectations of collective action theory and “bottom-up” perspectives to orient and interpret the data from the Carpathian Basin (*12*–*15*). We integrate the investigation of inequalities with measures of settlement density, site size, and longevity, long recognized as key parameters related to social stress and transformation (*16*). In addition, we introduce measures of social conformity and social cohesion as indices of prosocial or cooperative behaviors that fostered social interactions despite social stresses and the potential for social inequalities to increase. Although inequalities are ever-present, we find that over the course of 5000 years (~6000 to 1000 BCE), the sequence of change in the Carpathian Basin generally exhibits a limited increment in the disparities of house size within settlements. We argue that we can account for the repeated rise and collapse of inequalities in this sequence by invoking social institutions that promoted cooperation rather than accumulation, conflict, and social distinction, as illustrated by the persistence of community labor projects. We also point to the importance of the failsafe of “voting with one’s feet” in the trajectory, as our data highlight decreasing site longevity from the Late Neolithic (LN) onward. Dispersion acted as a pressure release for contexts of high conflict in which nascent hierarchies were rejected by local communities.

### Prehistory of the Carpathian Basin

**Fig. 2.**
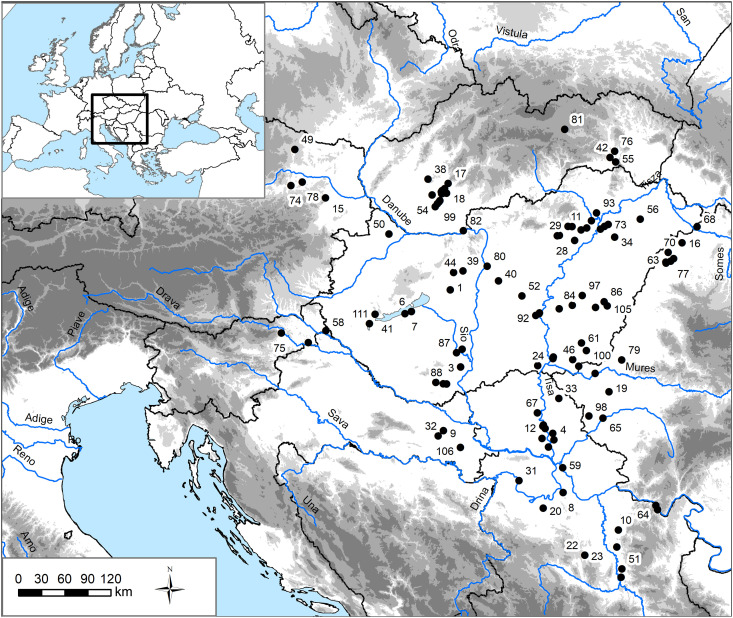
The Carpathian Basin, including sites used in the analysis. Site numbers correspond to ID numbers in data S1; site numbers not visible have coordinates listed in data S1. Modern country borders in black and modern rivers in blue. Base maps from Esri’s ArcGIS 10.8.

The Carpathian Basin is situated in central Europe and comprises a large sedimentary plain ringed by the Carpathian Mountains and drained by the Danube River and its tributaries (Fig. 2). The Basin’s rich record of systematically collected archeological data makes it ideal to explore the development of socioeconomic inequities. Over the past decades, a boom in development-led cultural heritage excavations, in addition to expansion in scientific investigations in all periods, has markedly improved our understanding of the timing and scale of social change in prehistory, providing a huge corpus of data upon which to draw (*17*).

The first farming communities from the Near East expanded through Anatolia and the Balkans, becoming established in the Carpathian Basin as the Early Neolithic (EN) Körös-Starčevo-Criş group around 6000 BCE (*18*). Their pit huts and small wattle-and-daub structures are found along the Danube and Tisza rivers and major tributaries, and their economy was founded on the Old-World domestic focus on cereals, cattle, sheep, and goat (*19*). The Middle Neolithic (MN) Linear Pottery culture (LPC) and several later MN cultures saw a major expansion of settlement into new territories of the Basin and an adaptation of the animal economy to local conditions between 5500 and 5000 BCE (*20*). In a small number of cases, settlements grew to 10 or 20 ha (*21*–*24*). Tell settlements, artificially raised mounds of settlement debris, began to grow in this period, and many eventually lasted hundreds of years. The first enclosures are founded late in this period, with deep ditches dug around settlements, sometimes with accompanying palisades (*23*–*25*). In the LN, ~5000 to 4500 BCE, settlement sizes grow even larger, this time up to 100 ha (*26*–*29*). Site enclosure becomes more common in some cases including multiple parallel ditches and palisades far beyond any functional requirement of fortification. The first formal cemeteries appear in the early fifth millennium (*30*).

However, by the Early Copper Age (ECA), ~4500 BCE, the size of villages markedly contracts (*25*, *31*). The dense populations of the LN disappear, and the equivalent in population disperses across the landscape (*32*). People still enclose sites but not at the scale of previous generations. Formal cemeteries become common. Then, ~3800 BCE in the Middle Copper Age (MCA), the visibility of human settlement drops again (*33*). The wattle-and-daub houses characteristic of domestic settings for 2500 years disappear, mostly to be left with sherd scatters, pits, and occasionally, cemeteries. Toward 3000 BCE in the eastern Carpathian Basin, the Yamnaya group enters from the east and builds thousands of burial tumuli (kurgans) but leaves little evidence of habitation (*34*). Economy turns to more mobile pastoralism, focused on caprines rather than cattle and pigs (*35*). This pattern continues for another 1600 years into the Bronze Age.

By the end of the Early Bronze Age (EBA), ~2200 BCE, wattle-and-daub houses again begin clustering into villages and grow once more into deep tell deposits (*36*, *37*). Cemeteries are founded, and, over the course of a few hundred years, sometimes grow to include thousands of burials (*9*, *38*). Beginning around 1900 BCE, settlements up to 20 ha reemerge and again become fortified around dense housing during the Middle Bronze Age (MBA) (*39*–*43*). This all ends by 1600 BCE, however, when fortified tells were abandoned (*44*).

During the Late Bronze Age (LBA), people continue to be buried in formal cemeteries but often in different places (*45*). Archeologically recognizable housing, however, again becomes rare in the Carpathian Basin. Starting in the 14th century in the southeastern part of the Basin, the practice of site enclosure reemerges but on a scale hitherto never seen. The LBA “megaforts,” as they have been called, can extend 6 km in diameter and cluster together across vast distances (*46*). Unlike in previous periods, these enclosures do not contain houses. Instead, there are “activity areas” and potentially the remains of some kind of dwellings. Although they are still poorly understood, researchers nonetheless argue for the presence of classes of elites and subordinates (*45*, *47*). As in the LN, the scale of ditch building goes far beyond any functional requirement of fortification (*48*).

The best single metric study of social inequality currently available for the Carpathian Basin across a broad swath of time is Wilkes’ calculation of a Gini coefficient, based on burials, for the MN to the MBA (*10*). In this study, changes in inequality appear to be relatively short lived, as the average Gini coefficient remains largely stable between 5200 and 1000 BCE, shifting insignificantly from 0.73 to 0.75. However, there is a notable increase in the variance of inequality during this period. This is evidenced by the much wider range of Gini coefficients observed among Bronze Age cemeteries, where the gap between the highest and lowest values reaches 0.26 compared to just 0.02 in the LN. This growing disparity is also reflected within specific segments of society and is corroborated by other inequality metrics, such as generalized entropy measures.

If we were to expect incremental increases in inequality after agriculture has been established in the region, then we would see it over this 5000-year span. Similarly, if a transformational new technology such as plow agriculture made a great difference, then we would expect it as early as the fifth millennium BCE (*49*). Similar to others cited above, specialists in the Carpathian Basin often argue that the Bronze Age, ~2500 years after the introduction of farming, was the point at which social inequalities became substantial and enduring (*50*, *51*). Were this so, we would expect greater inequality in the later part of the sequence than the earlier part.

## MATERIALS AND METHODS

We calculate wealth inequality (house size Ginis) and contextualize it with other metrics built from archeological data—site size and density, site apogee, social cohesion, and social conformity. These measures satisfy relevant parameters for understanding collective action and are measurable across time and different cultural traditions. Our sample from the Carpathian Basin includes data from 110 sites, although few sites could provide data on all proxies.

The time frame of the sample is differentiated into the Neolithic, Copper Age, and Bronze Age, each with an Early, Middle, and Late subphasing. Table 1 illustrates which variables could be coded for which chronological phase. Our criterion for including sites in our sample was the presence of at least one of six variables (beyond radiocarbon dating) coded in our methodology. Additional methodological details, including site dating, calculation of house Gini value, social conformity, site size, and density estimation, are provided in the Supplementary Materials. Extended tabulated details of individual sites are found in data S1, and radiocarbon dates are found in data S2. The statistical analyses and figures were produced in R and are available on GitLab (https://gitlab.com/bilottigiacomo/5000-years) and Zenodo (10.5281/zenodo.15274708).

**Table 1.** Study sample by fine chronological period and coded variables.

	EN	MN	LN	ECA	MCA	EBA	MBA	LBA
**Dates BCE**	**6000**	**5500**	**5000**	**4500**	**3800**	**2700**	**2200**	**1500**
Site size	5	13	27	4	4	2	23	12
Density	7	21	19	3	3	1	4	5
Social cohesion	0	2	10	3	3	2	26	7
Social conformity	8	20	21	3	3	1	6	5
Gini	8	22	21	3	3	1	4	6
Apogee	6	7	16	1	2	0	3	5
**Total sites**	**8**	**23**	**28**	**5**	**5**	**2**	**26**	**13**

### Gini coefficient of inequality

The Gini coefficient calculates inequality using the distribution of some resource or resources in a population (*52*, *53*). For example, grave goods deposited in a cemetery population can be added together to calculate the fraction that each member held within it. Those numbers are then converted to a single value between 0 (complete equality) and 1 (complete inequality) for comparison with other cemeteries. The Gini coefficient has been criticized as a simplistic measure of inequality, as it does not capture where within the distribution inequality occurs or does not reflect shifts in wealth concentration over time. For example, two populations with very different internal wealth structures can share the same Gini value (*54*, *55*). It also performs relatively poorly with small samples, although this is common with most dispersion measures (*56*). Nevertheless, the Gini coefficient remains widely used due to its general familiarity and its usefulness for making straightforward comparisons between sites or groups.

Gini values built on house sizes and mortuary data produce different results, as they constitute different forms of wealth (*52*, *53*, *57*, *58*). Gini values for cemetery data tend to be higher, especially when burials without grave goods are included in the calculation (*52*). Disparities in mortuary display reflect relational differences between the deceased. Once buried, differences in grave goods (if outside domestic spaces) are less enduring than material wealth such as houses, which can be inherited and persist across generations, even if modified and rebuilt (*11*, *59*, *60*).

We calculated Ginis using the outlines of houses to approximate their size as a measure of wealth and therefore variation between contemporary communities and communities over time. We only calculate a Gini coefficient if five or more houses could be recognized using both excavated and remote sensing (magnetometry) data (data S1, “houses”). To accommodate small sample sizes, we used a normalized Gini coefficient. The specific formula is found in the Supplementary Materials.

### Site size and density

Additional social variables help us understand the Gini values and their variation in our sample across space and time. Site size and density are often considered indicators of higher social complexity in ancient societies, as the number of social interactions increases with both, possibly creating organizational stresses that affect resilience (*61*, *62*). Where higher population densities and larger site sizes are accommodated, greater social cohesion might be expected, especially if nested in neighborhood groupings (*63*). Eventually, institutions, including social hierarchies, intervene to reduce stress, but we might expect them to come with heightened levels of social inequalities (*3*). Enduring large site sizes are often attributed to permanent political authorities and, therefore, greater inequalities.

Site size estimates are taken directly from site reports and publications whenever possible but sometimes using Datinf software when needed and feasible. We measure settlement density by dividing the settled area by the house floor (in hectares). Only sites where house Gini values could be measured (five or more structures) were used to calculate density. We measure density by focusing on housing area only. Communal areas, ditches, and other settlement features are excluded.

### Site apogee

The persistence, or apogee, of a settlement might also be related to inequality levels. Our intention in measuring apogee is to calculate the length of an unbroken span of time during which a site, its houses, and ditches were constructed and used, in keeping with the focus on the dominant use-life of a settlement in the literature (*64*). Intensified agricultural production has been shown to be weakly positively correlated with apogee and is often related to governance (*14*, *64*–*66*). Higher levels of inequality in political leadership tend to lead to slightly shortened settlement duration (*14*, *64*–*66*). At the same time, the calculation of apogee lengths allows us to assess the durability of inequality regimes. Analyses of this relationship support the idea from collective action theory that community institutions such as those related to protection and hosting feasts sustained growth, helped regulate the stress of communal living, and attracted newcomers. The length of a settlement’s life is also an indicator of a community’s resilience and sustainability (*67*, *68*). To date, mostly central places or urban centers have been investigated for apogee and only using relative chronological data.

We calculate the duration of a settlement using the median (med) and SD of radiocarbon data in OxCal 4.4’s span function (script in the Supplementary Materials, radiocarbon dates in data S2, and OxCal output statistics in data S1, “apogee”) (*69*, *70*). We measure apogee where sites have a minimum of five radiocarbon dates and an attempt by archeologists to date the earliest and latest component. We require an unbroken sequence of occupation during which the ditches were arguably used (generally less than 75 uncalibrated years between dates). We also inform our modeling with absolute dating and discussion undertaken by researchers who have worked on our site sample and compare settlement duration estimates. The study includes 27 previously unpublished radiocarbon dates from the sites of Drenovac, Úľany nad Žitavou “Dolné diely,” Vinkovci-Sopot, Vlkas, and Vésztő-Mágor.

### Social cohesion

The social cohesion of a community is observable through architectural layouts, feasting, collective mortuary behavior, and other material manifestations of communality (*71*–*74*). Most such features are not present across thousands of years in comparable ways, but one exception is the organization needed to construct communal ditches around shared spaces. Regardless of their primary function, ditches and fortifications required many people involved in their construction and signal the cohesion and collective actions of a community (*75*). Ditches are often considered fortifications, but decades of research on ditches and enclosures in European prehistory indicate that many served a variety of other functions (*76*, *77*). In addition, collective, ritualized monument construction is often identified as an activity that channels human energy and fosters cooperation rather than being a measure of the coercive power of leaders. Both ethnographic and archeological case studies point to the ability of even small-scale societies with ephemeral leadership structures to undertake monumental constructions requiring hundreds of thousands of person-hours (*16*, *78*). Although the absence of ditches in our sample does not by definition imply a lack of social cohesion, it is the most visible sign of cohesive activity in the Carpathian Basin demonstrated across 5000 years. We estimate the sediment moved as the volume of a triangular prism, calculated using the formula for V-sectioned features (see additional details of method and sites in the Supplementary Materials and data S1, “ditches”).

### Social conformity

The variable of social conformity is based on the premise that the way in which built space is shaped, especially as a result of the placement of houses within a settlement, can be seen as a reflection of the degree to which the agents constructing those houses conform to an overall rule, aligning their house with others, creating an overall formal structure of rows, circles, or otherwise (*79*). This assumption is based on practice theory (*80*–*82*) and space syntax theory (*83*), which furthermore stress the effectiveness of such built orders in maintaining formal social relations for the house inhabitants [see also more recent settlement scaling theory (*84*)].

To determine the degree of social conformity, two-dimensional house plans were coded on a scale from 1 (each house individually placed, no visible overall order) to 5 (more than 80% of houses strictly accord to a structure) (table S3). The presence of a similar house orientation is counted as an extra point, effectively resulting in a scale from 1 to 6. We would expect that lower conformity to social rules expresses weaker intracommunity relationships and lower resilience. In relation to site size and density, we would expect more order and integrative structures as these values increase.

## RESULTS

**Fig. 3.**
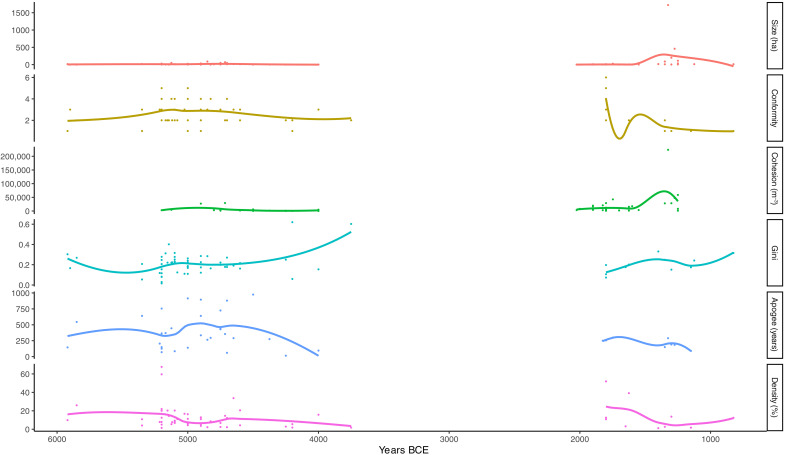
Results of coded variables over time.

We present inequality values next to the results from other variables associated with social stress and resilience on a timeline in Fig. 3.

### Inequality

**Fig. 4.**
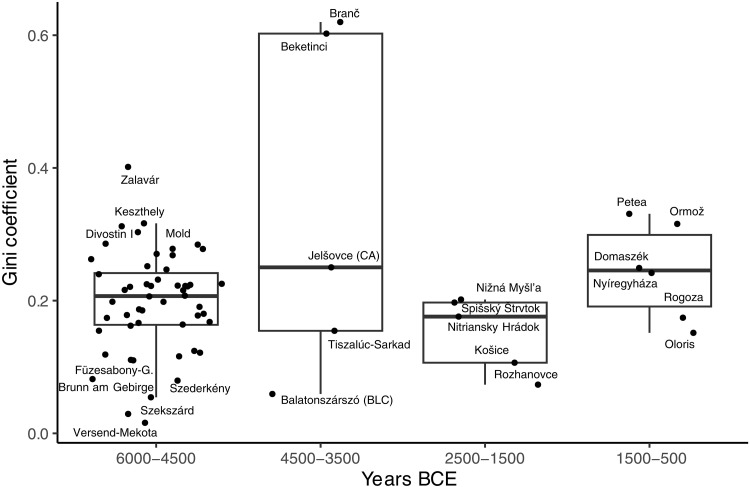
Gini coefficients by time frame. BLC, Balaton-Lasinja-Culture; CA, Copper Age.

The Gini values for the houses in the study range between 0.016 and 0.620, averaging 0.209 (table S1). We illustrate trend lines in Fig. 3 with local polynomial regression fitting, which uses nearby points to weight the line, but there is a large gap in the data points for all variables between 3800 and ~2000 BCE (see the “Prehistory of the Carpathian Basin” section). For this reason, we group sites into “early” and “late” prehistoric groups for Welch’s two-sample *t* tests. Gini values do not significantly differ (*t* = −0.315, df = 17.97, and *P* = 0.757). Mean values are not different from one phase to the next (early = 0.210 and late = 0.202), although there is a high variance (and small sample size) in the 4500–3500 BCE period (Fig. 4).

The highest house Gini values (~0.6) are Beketinci and Branč, 1.5-ha ECA sites that do not stand out in other ways. An apparent trend toward increasing Gini values in the Bronze Age is not significant (*S* = 106.97 and *P* = 0.106). It is unlikely that the type of measurements available for early and late periods affects our conclusions, as the measurements for the Bronze Age come exclusively from excavations and apex sites such as fortified tells, where we would expect the highest degree of inequality (*85*).

### Site size

**Fig. 5.**
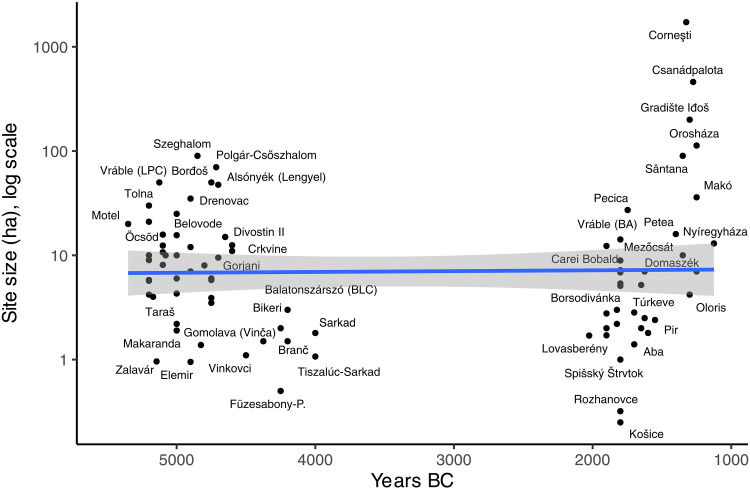
Site size (log scale) over time. BA, Bronze Age.

**Fig. 6.**
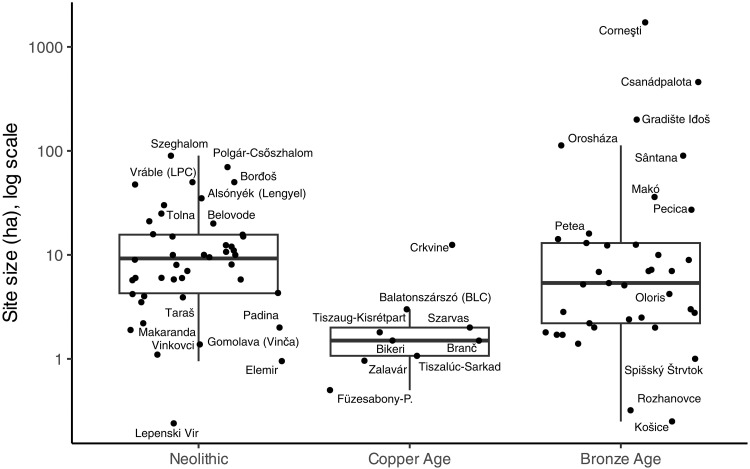
Site size (log scale) by period.

Mean site size is not different between the early and late periods (*t* = 1.312, df = 36.196, and *P* = 0.198; Fig. 5), although the LBA megaforts are obviously unprecedented in the early period. The mean values of the Neolithic and Bronze Age are nonetheless quite different because of the outliers (early = 13.35 ha and later = 75.97 ha), but MBA settlements were still only a quarter of the size of the largest in the LN (Fig. 6).

### Site apogee and social cohesion

**Fig. 7.**
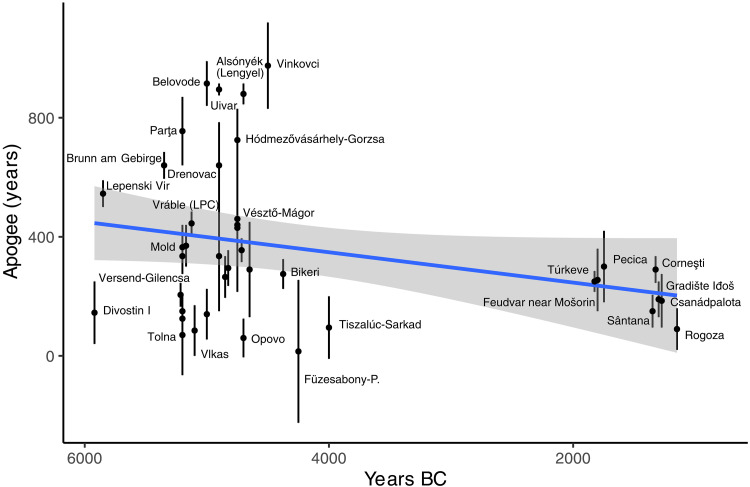
Site apogee over time.

Site apogee, calculated using median radiocarbon date spans, exhibits a clear temporal trend. Over the course of 5000 years, site apogee significantly decreased (*t* = −3.316, df = 37.756, and *P* = 0.002; Fig. 7). In the Neolithic, well-dated sites such as Belovode, Hódmezővásárhely-Gorzsa, and Parța highlight that settlement apogee is much longer earlier in the prehistoric sequence (early = 398 years and late = 214 years). Despite the enormous size of some of the LBA sites, the data indicate that they were nevertheless short lived.

**Fig. 8.**
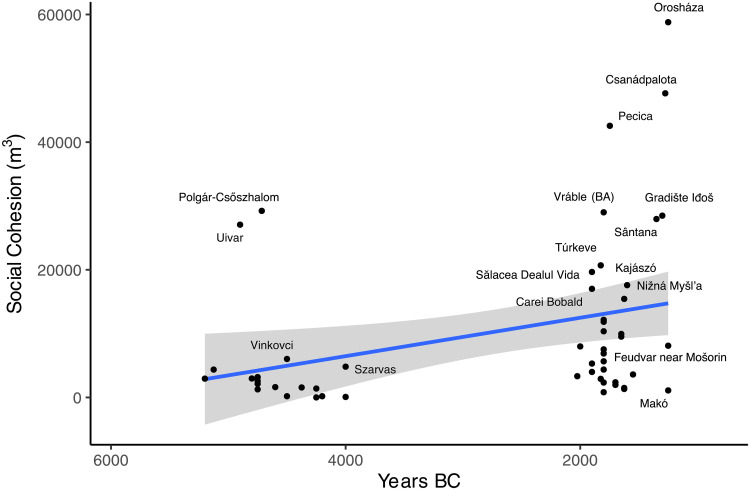
Social cohesion scores (ditch volume in m^3^) over time. Corneşti, an outlier at 223,692 m^3^, is not shown.

Social cohesion scores, measured using community investment in ditch construction, also present an unambiguous temporal trend (Fig. 8; *S* = 15,514, *S* rho = 0.375, and *P* = 0.006). High values can be found in the LN (early mean = 5021 m^3^), but ditch construction reaches its apex in the LBA (late mean = 19,505 m^3^), especially clear when we include identifiable ditches at sites that have not seen excavation (Fig. 8 and fig. S1). Despite their short-lived nature, the megaforts are a scale of construction. Corneşti (1722 ha), the largest, has 220,000 m^3^ of tested ditch volume and an additional 20,000-m length of ditches untested and unmeasured (table S2). LN sites such as Polgár-Csőszhalom (29,232 m^3^) and Uivar (27,071 m^3^), however, were already in the lower ditch bracket of LBA sites by ~4800 BCE, demonstrating an impressive ability for communities with little demonstrable social inequality to organize labor for joint infrastructure investments.

### Social conformity and site density

Social conformity scores, measured using the degree of organization and symmetry in settlement layout, differ between periods (Fisher’s exact test, *P* = 0.018, early = 2.7, and late = 2.4) (fig. S2). They also exhibit a temporal trend in the Bronze Age, where conformity falls over time between 1800 and 1100 BCE (fig. S3). Housing density does not show appreciable change over time (*S* = 51,109, *P* = 0.074, and *S* rho = −0.227; fig. S4), and housing density is overall very low (mean early = 14.94% and mean late = 11.07%). There is no relationship between density of housing, size, social inequality, or other variables (e.g., fig. S5).

## DISCUSSION

The present dataset includes much of central Europe over 5000 years and suggests that the global narrative on the inevitability of growth in inequalities, and the role of new technologies in amplifying them, is premature. Gini values from houses are at odds with the cemetery Gini values, where variance is highest toward the end of the sequence, in the MBA (*10*). Hence, the Gini values for the house sample are not similar to the values observed in the cemetery Gini dataset for the same periods, although cemeteries and settlements available for comparison do not draw on the same communities.

Lack of statistical change in wealth in both burial and house size Gini data over 5000 years contrasts with patterning often described for social dynamics after the onset of farming and especially since the introduction of land-limited agriculture (*1*, *2*, *86*). Our findings are nonetheless consistent with previously published Gini values for the Balkan Neolithic ~5000 BCE (*1*, *87*). However, increases in inequality starting in the third and second millennia in the Near East are not present in the Carpathian Basin, highlighting the different properties of these samples.

In the Copper Age, housing is far less durable than that of the wattle-and-daub structures of previous millennia, and where present, settlements are much smaller, with less social conformity. The sites of the earlier Bronze Age are again recognizable as settlements with houses, but differences in house size do not rise even to the modestly high Gini values seen in the ECA. A similar drop in the visibility of settlement takes place in the LBA, although the many sites and megaforts attest to potentially large populations (*46*). Higher density or larger site size might be expected to correlate to some degree with higher Gini values given the association of greater population sizes with greater complexity or inequality (*61*), but neither settlement density nor site size is correlated with Gini values in houses in our sample. This may not be surprising for this part of Europe, where recent research at the Trypillia megasites east of the Carpathian Basin indicates that house Gini scores fall as the largest sites are founded and grow (*8*).

Communal property or land-use rights, however, probably played important roles in all periods, as social cohesion scores demonstrate. Ditch, palisade, and even rampart construction were common features of village life in the Carpathian Basin that undoubtedly took great community focus and would have required regular maintenance and coordination (*86*). At the largest Neolithic settlements and Bronze Age megaforts, integrative architectural structures are known or suspected and likely facilitated large settlement populations and gatherings (e.g., as at Parţa, Polgár-Csőszhalom, Sântana, and Lăpuş) (*88*, *89*).

The shortening of site apogee over time is a clear and surprising trend in the data and an indication that social cooperation over time need not correlate with increasing long-term coresidency. Apogee peaks in the LN and then falls, perhaps expectedly, as ECA cemeteries demonstrate incipient forms of inequality. Decreasing settlement longevity from this time forward could have been a way further inequality was avoided, using the time-tested strategy of “voting with your feet” [see (*90*)]. Social and environmental circumscription preventing such dispersals are implausible in the study area given prehistoric population estimates [e.g., (*39*, *91*)]. Neither settlement density nor increases in cooperation over time seem to promote greater site apogee, given that the largest sites in the sample are among the shortest lived. This contrasts with areas such as central Mexico, where greater infrastructural investment is positively correlated with apogee length (*64*).

### Concluding thoughts

When identifying inequality in Bronze Age Europe, archeologists generally highlight not house size variation but evidence from cemeteries where people are buried with varying amounts of grave goods such as ceramics, metals, or exotic items. There are certainly inequalities in the distribution of wealth in the graves of the Carpathian Basin, with variation identified between cemeteries (*9*, *10*, *92*, *93*). However, we emphasize that the wealth of the minority does not suggest social stratification and the power of the few over the majority. Rather than asserting the coercive or dominative abilities of those associated with burial wealth, the aspirational differences between people represent the celebration of the deceased with their possessions from life (*9*). The settlements of a few hundred people with which these graves are associated seem more indicative of communities with collective, decentralized leadership without coercive power [(*13*), but for exceptions, see (*94*)]. Site size hierarchy visible in many parts of the Carpathian Basin is easily explained by factors besides social stratification (*95*). Animal technologies such as equestrianism introduced new ways to resist central authorities and growing inequalities as much as they offered new ways of asserting power (*96*).

Moreover, cemetery wealth consists of trade goods, prestige goods, and locally made objects placed with the dead rather than inherited by the living. For this reason, cemetery Ginis represent relational wealth, a wealth based on individual personalities and positions in social networks (associated trade partners) that are not heritable, as personal ties are often terminated at death (*11*, *60*). Wilkes’ Gini analysis of 6000 burials in the northern Carpathian Basin dating between ~5200 and 1000 BCE found little evidence of changes over time when analyzing the mean values in the single time periods, but the range—the distance between the maximum and minimum value—nonetheless indicates a widening tolerance for inequalities in some communities in the Carpathian Basin (*10*). The widespread introduction of weights and balances ~1500 BCE, combined with the fragmentation of bronze, is consistent with metal as a currency, a stable form of value, and easily inherited wealth (*97*).

The lavish burial rituals of the MBA nonetheless return to near-invisible housing and limited mortuary display during the LBA (*98*). In much of the Carpathian Basin, the LBA may have seen an increasingly mobile society with a pastoral way of life. Although it is also possible that LBA societies exhibited inequalities in access to animal herds, and the concordant heritable inequalities such resources have (*99*), there is currently little evidence of it. Instead, the dynamic interactions between settlement size and density, social conformity, cohesion, and settlement apogee indicate an ability for community residents in this sample to mute the growth and institutionalization of inequalities.

In sum, it is not that inequalities were never present in our sample but rather that inequalities were never great and did not continue to grow in multiple domains as they did in the Near East. It is also possible that European sites such as Knossos and tell societies in northern Mesopotamia were land limited in a way that those of the Carpathian Basin never were [see (*1*, *100*, *101*)], with the latter exhibiting more intensive management of production more similar to southwest Germany in the Iron Age (*102*). Although plow agriculture may have been commonly used in the study area during the third and second millennia BCE, strict limitations on land may have rarely been a concern. More empirical comparisons of environments in areas with different degrees of inequality would be important in identifying the importance of this factor. A recent global-scale evaluation of the impact of greater productivity, including plow agriculture, indicates only the growing potential for inequality with their introduction, not its realization (*103*).

Our findings problematize recent conclusions using Gini coefficients, some regional, and some cross-cultural. Regionally, the Carpathian Basin sequence does not support the assertion that durable social inequality continued to ratchet up after the Neolithic [see (*104*, *105*)]. The heritability of wealth varies depending on the form it takes, with material wealth (such as houses, property, and animals) being very heritable and relational wealth, such as networks of trade partners, much less so. Although the inequalities were able to manifest in our sequence, they were never very great or did not last for very long.

Notably, the proxies used here do not allow a fine-grained understanding of why some communities tolerated growing social inequalities or precisely why they ended up dissolving. While our data provide important context for patterns in inequalities in the Carpathian Basin, site-based and microregional scales and chronologies are critical for sensible and nuanced appreciation for social stability or change visible in the sequences. We are also aware that the apogee measurement, dependent on the collection of radiocarbon dates, is biased by research tradition such as a particular focus on the EN (*106*) and the fact that flatter areas of the radiocarbon curve might lead to longer span estimates.

In a broader comparative sense, however, we fail to see the inevitabilities projected at the onset of farming proposed by some influential voices. During the temporal sequence we investigated, we found no indication that mass mobilization warfare, revolution, state failure, or lethal pandemics interrupted the development of inequalities in our sequence (*6*). The logic that plow agriculture, once introduced, was sufficient to disrupt extant cultural practices regarding interhousehold inequality is incorrect or, at least, insufficient. Traction animals and extensification may have played an important role in the development of urbanism and inequality in fourth-millennium–BCE Mesopotamia (*107*), but the same outcomes do not characterize the Carpathian Basin sequence, although the same technologies were available. We anticipate that the interaction between other parameters, including those investigated here, may end up being critical in understanding the necessary conditions for enduring inequality.

## Supplementary Materials

### The PDF file includes:

Supplementary Text Figs. S1 to S5 Tables S1 to S3 Legends for data S1 and S2 References
