# Supplementary material for: Five thousand years of inequality in the Carpathian Basin
Source: Sci Adv. 2025 Aug 6;11(32):eadu0323. doi: 10.1126/sciadv.adu0323 (PMC12327458; doi:10.1126/sciadv.adu0323)
Supplement: Supplementary file 1 — Supplementary Text Figs. S1 to S5 Tables S1 to S3 Legends for data S1 and S2 References [file sciadv.adu0323_sm.pdf]

Science Advances
AAAS

Supplementary Materials for

Five thousand years of inequality in the Carpathian Basin

Paul R. Duffy *et al.*

Corresponding author: Paul R. Duffy, prduffy@ufg.uni-kiel.de

Sci. Adv. **11**, eadu0323 (2025)
DOI: 10.1126/sciadv.adu0323

The PDF file includes:

Supplementary Text
Figs. S1 to S5
Tables S1 to S3
Legends for data S1 and S2
References

Other Supplementary Material for this manuscript includes the following:

Data S1 and S2

Supplementary Text

How sites were assigned mid-date
Using absolute dates for individual sites, we have calculated a mid-date of site occupation based on the average of the earliest and latest date. If radiocarbon dates were not available, we used the generally accepted absolute dates for the region the site belongs to.

Calculating house Gini coefficients
We restricted measurement to settlements that have plans from five or more houses. Consequently, like cemetery Gini values in this region, house measures suffer in some periods (especially ca. 4000-2300 BC) when houses and burials are very rare. The house sizes were mostly measured from excavation or geomagnetic plans using the software datinf®, but we used measured values by the excavator when available. When excavation or magnetic images were measured, we included only clear structures whose outlines could be estimated. Special structures or exceptionally large buildings were included in the calculation of the Gini index only in cases where the respective publication states a domestic purpose of such buildings, such as at Banjica (*108*) or Branč (*109: 326*). Clear non-domestic structures, like the Sanctuary of Parţa (*110: 204-246*), were not included in the calculation of the Gini index. We avoided including ambiguous structures where investigators were uncertain of the nature of features, such as the sub-rectangular features associated with post holes in LBA mega-forts (*111*). In general, sites were treated as single-phase phenomena except when a clear distinction was given in the literature. Investigations at Drenovac showed that the size of anomalies correspond with the sizes of the excavated structures (*112*).

Since the Gini index is derived from the relation between the line of total equality and the Lorenz curve (line of observed inequality), we calculate the Gini indices using the formula for the area of a trapeze (see below). Our formula recognizes that the maximal possible Gini index of a given site is based on the sample size (*113: 92*). The reported Gini indexes (G) for each site therefore are normalized Gini indices (also known as Lorenz-Münzner coefficient (*114: 53ff*) with R code by (*115, 116*).

$$G = \frac{2\sum_{i=1}^{n} ix(i)}{\sum_{i=1}^{n} x(i)} - \frac{n+1}{n}$$

$$ASE(G) = \sqrt{\frac{1}{(n\bar{x})^2}\sum_{i=1}^{n}(Z_i - \bar{Z})^2}$$

$$CI_{0.8}(G) = [G - |z_{0.1}|ASE(G); (G + |z_{0.9}|ASE(G)]$$

Apogee site list
Some of the sites in our sample have had modeling of the dates and duration estimates provided by the excavators and radiocarbon specialists, but most sites do not have modeled sequences using stratigraphic data. Consequently, we generate spans confined to a single Oxcal phase to

make them more comparable but note discrepancies between our span estimates and those of other experts based on different modeling criteria. If five or more dates were available for dating but we consider them unrepresentative, we explain why they were excluded.

Some sites have over one hundred dates, making the calculation of span a little different from sites which have 5 or 6 dates. For example, the ditch construction at the Late Bronze Age for Cornești begins ca. 1400 BC (Phase 2) and ends that same century. Phase 3 and 4 have several dates but are isolated pits and colluvium in ditches. Below, we exclude Phase 4 and explain our reasoning. In general, we aim to correspond well to the archaeological discussion of site history (e.g. *117, 118*). We note which radiocarbon dates were excluded from the Oxcal model to calculate apogee (below) and list all relevant dates in Data S2. We use the median value for apogee to reduce the impact of extreme outliers, but we compare means in the text below because they are more comparable to the published literature.

Alsónyék (LPC and Lengyel). The Alsónyék site has three Neolithic / Copper Age components; all have been well dated, but we include in this study only the Linear Pottery Culture (LPC) and Lengyel components. The earliest occupation is the Starčevo (*119*), but there is a hiatus of about 200 years between this component, and the next phase, the LPC (*120*). The LPC is continuous with the Lengyel component, the dominant occupation at the site, with almost 60 dates (*121*). One late outlier is removed (Poz-68348, 5355+/- 35). There is extensive modeling and discussion of the absolute chronology of the Alsónyék site. Researchers calculate a continuous span of occupation between 940 and 1130 years (68% probability) (*122: 293*), in broad agreement with our mean calculation of 860, if somewhat longer.

Balatonszárszó-Kis-erdei-dűlő (LPC and Lengyel). The LPC and Lengyel components may be continuous at this site. Three LPC dates can be found in (*123*), reproduced with additional dates in (*124*). The Lengyel component has one date in (*120*) placed a thousand years later than the LPC component (OxA-13784) and is not included.

Belovode. There are 17 dates and five horizons modeled for the sequence (*125*). The earliest horizon begins ca. 5500 BC during the Starčevo, ending during Vinča D2, ca. 4500 BC. All dates including residuals in their model are included in our calculation, which does not discriminate between phases, providing a mean of 925 in broad agreement with their conclusions.

Borđoš. The site has multiple components, primarily the Late Neolithic and Late Bronze Age. We modeled the Neolithic component, which has thirty-nine dates. Like the excavators did for their model, we removed two outliers from ours (Poz-63490 and Poz-63491) because they are much earlier (*26: 11*). Hofmann et al.'s model spans 5025 to 4515 BC, a good agreement with our estimate of 420 years.

Brunn am Gebirge. The site has over 100 dates, has been subdivided into phases, and has extensive chronological modelling (*126*). The excavators date all phases to 490 years, shorter than our unphased mean estimate of 640 years (*126: 223*).

Corneşti-Iarcuri. The most definitive publication is (*118*), which includes 109 dates, discusses taphonomic processes, sample type, outliers, and the different chronological components of site use. Ninety-one dates come from the LBA and EIA. Settlement phase I of the LBA begins at the

transition from the 16th to 15th century, when Ring 1 was built, and when the areas inside the fortification were settled and buildings were constructed. Ring II was built next at the turn of the 14th century, during Settlement phase 2. There is continuous settlement into the 12th century. Though there is a Settlement phase 3, there is almost no overlap in 2 standard deviations with Settlement phase 1, indicating a break. Researchers model the major component of use to be ca. 400 years (*118*), yet given the large number of dates involved in the calculation, we also exclude some MBA and LBA dates (MAMS-35586, MAMS-26690, MAMS-26689, MAMS-29744) that show a break in the main LBA sequence, generating a shorter mean of 290 years.

Csanádpalota-Földvár. There are 10 radiocarbon dates from the site, nine from the LBA, and all included here (*127*). The tenth (DeA-3471) dates to the sixth century BC. Given the current evidence, the excavators estimate a range between 1430 and 1120 BC, ca. 300 years during which all enclosures were built, though some earlier ditches seem to remain open for the sequence (*127: 240*). Their dates are unmodelled however, and slightly longer than our mean estimate of 200 years, but within our 2 s.d range (35-390 years).

Divostin. The main published sources of dates are (*128*), (*129*) and (*130*). Divostin I (ID 22), the Starčevo occupation, is continuous. There is then a gap of several hundred years. Divostin II (ID 23) sees reoccupation during the early Vinča, and then another gap of several hundred more years, before two final dates. All Starčevo dates are used for the Divostin I apogee, and all but these last two are included for the Divostin II Vinča (Bln-867 and BM-574).

Drenovac. Excavators say that the site was occupied in the Starčevo (6100-5900 BC) and Late Vinča culture (5300-4700/4500 BC), but dates are published by (*131*). We publish four new dates here. While four dates fall within the Starčevo noted by the excavator, a single date (BRAMS-2239) falls in between these and the Vinča as well. The Starčevo dates were too few to meet requirements, and the anomalous Vinča date is excluded. The rest of the Vinča dates are included.

Feudvar near Mošorin. Eleven dates for House 24 are published by (*132*), all are included. Because the wood charcoal from the roof and wall possibly suffer from 'old wood', and the cereals and short lived samples are statistically indistinguishable, Roeder suspects that the lifespan of the house the samples come from are ca. 250 years or less (*132: 274*), and finds the possibility that the span was up to 500 years rather unsatisfactory. Roeder's estimate is consistent with our mean estimate of 255 years. Additional thoughts are found in (*133*), who uses an early computer simulation of the dates. The author also reports there are 47 dates total, yet to be published.

Füzesabony-Gubakút. Domboróczki (*134*) modeled the development of the site into five phases in 2005 and provided a more refined chronology of 12 subphases with additional dates in 2009. His model of initial occupation 5560-5405 BC with an abrupt depopulation after 5220 BC is comparable to our mean value of 340 years. An obvious late outlier (Deb-5871) is excluded.

Füzesabony-Pusztaszikszó. There are six Copper Age dates, all included (*135*). One Bronze Age date (DeA-6747) is excluded (*136*). Raczky et al. calculate the span as 0-120 years, with one

standard deviation (*135: 562*). Our mean is somewhat higher (185 years) with a larger standard deviation (0-405 years).

Gomolava (Vinča). Thirty-three dates are published, primarily found in (*137*) and (*138*). The excavators distinguish three phases, all within the Vinča B2-D period. Orton models them by phase, excluding charcoal, beginning ca. 4800 BC, and ending 4650, shorter than our mean estimate of 300 years. Our model span is the same as that calculated by (*137*). There are four outliers dating to the Late Copper Age component (GrN-7371, GrN-7373, GrN-7375, OxA-21132).

Gradište Iđoš. All dates are collected in (*46*). Two non-overlapping Bronze Age phases are modeled and the first of which, when the enclosures were constructed, lasts about 200 years, comparable to our mean value of 180 years. None of the dates from the later phase come from the ditches, suggesting an abandonment and reoccupation of the site. These younger dates are excluded (KIA-51813, KIA-51809, OXa-38713, OxA-38715, OxA-40223, OxA-38224).

Hódmezővásárhely-Gorzsa. The site dates were summarized and modelled in (*139*). Shell based dates for earlier strata were then provided by (*140*). All are included. The dates were most recently modeled by (*141*), who employ a charcoal outlier model, lasting 4850-4400 BC, almost 500 years. This generates a shorter timespan than our model mean value of 735 years.

Hódmezővásárhely-Kökénydomb. There are 10 dates initially published in (*142*), and re-calibrated with a couple of others and modeled by (*139*). All are included here.

Kakucs-Turján. The excavators define eleven Early and Middle Bronze Age phases based on the relative stratigraphy, but only 12 dates are available for six of the phases (*143, 144*). These dated phases run approximately 1850-1700 BC, though the excavators argue based on the relative chronology of the earlier ceramics that the site was in use starting ca. 2400 BC. This discrepancy makes it impossible to use the modeled dates alone, so the site is excluded from analysis.

Lepenski Vir. The site is occupied during the Mesolithic and Early Neolithic, and has over a hundred dates for the sequence. Radiocarbon dating places the site in the Early and Middle Mesolithic, called the Proto-Lepenski Vir phases, with a gap in the Late Mesolithic (*145*). Occupation resumes during the Transitional phase, ca. 6100 BC, with the arrival of the Neolithic to the Central Balkans.

The Transitional Phase, Lepenski Vir phases I-II, probably lasted only 140-190 years (*145: 29*). The trapezoidal structures used to construct a Gini value come from this phase. Phase III is the Early Neolithic Starčevo culture, when these structures were backfilled, though occupation at the site continues. Arguments have been made that there was a gap between phases II and III based on stratigraphy, but the radiocarbon dates contradict this idea. At the onset of Phase III, there is nonetheless a change in material culture, house style, and mortuary rituals. Abandonment of the site takes place 5720-5540 BC (68%), indicating a continuous span of occupation of about 500 years. For calculation of span we have relied on Lepenski Vir I-III as detailed in Table 1 of Borić (*145*), using values corrected for the freshwater fish effect. We arrive at a similar mean span of 545 years.

Mold "Im Doppel". There is one series of continuous dates across houses at the site (*146*). They calculate date ranges for individual houses, but they do not calculate a modelled span for the the occupation. The first house starts at 5300-5260 and the latest house dates between 5060 and 5040 BC, providing a slightly shorter estimate than our estimate of 375 years.

Öcsöd-Kováshalom. There are two series of dates, the first from excavation of the settlement (*147, 148*). Several were abandoned to create Harris Matrix based chronological models in (*149*), which yielded a span of 0–610 years for Tisza I and a span of 0-193 years for Tisza II. The second series derives from excavation of a ditch falling within this these phases (*150*). The whole sequence is argued to fall between 5363/5266 and 4894/4771 BC, drawing the chronology into the Szakálhát period, slightly longer than the mean span of 380 years we calculate.

Opovo-Ugar Bajbuk. Ten dates are published for the three phases of the site by (*138*), two of which were dated. The model suggests a brief occupation of 80 years, consistent with our mean estimate of 75 years.

Parţa. There are two spatial components to this site, Parța 1 and Parța 2 (*110*), and both are used in the calculation of radiocarbon span. Both sites have Early Neolithic Starčevo habitation, though Parța 2 has more of it (ca. 30-40 cm) (*151*). Most evidence of habitation is from Parța 1 during the Middle to Late Neolithic, Banat Phase II. Level 7b (which contains the houses with Gini values produced in this paper) dates to this phase. Parța 1 was mostly abandoned at the beginning of Vinča C, when Parța 2 again has more evidence of use, followed by Tiszapolgár and Medieval deposits. The Belgian dates (Lv- series) are often cited as coming from Parța 2, but they are corrected in the Uivar monograph and given more precise contextual information.

Bayliss et al. model the Middle-Late Parța 1 dates on the basis of the stratigraphy and using the charcoal outlier model for the 11 bulk charcoal samples (*141*). Their model suggests the Neolithic occupation began 5535-5335 BC, ending 5130-4945 BC (68% probability).

We calculate the span by including the Starčevo dates from Parța 2 as well, even though there is a gap of 140 uncalibrated radiocarbon years between them and the Banatuli culture dates. We do this because only relatively small areas of the tells were investigated, and based on the continued finds across both tells for the entire sequence, the settlement was likely continuous. Our model results in a mean span value of 765 years.

Two dates were excluded because their provenience is unclear (OxA-7453, Ly-8935).

Pecica-Sanțul Mare. There are twenty Bronze Age dates published for the site, and all are included (*152, 153*).

Polgár-Bosnyákdomb. There are seven dates, all dating to the backfilling of the ditch around the site, and corresponding to a short time frame (*154*). Based on collected ceramics at the 6 ha site there is occupation leading back to the Middle Neolithic, though the Late Neolithic material dominates. For this reason, we consider the available radiocarbon dates at the site unrepresentative and we do not calculate the span for our dataset.

Polgár-Csőszhalom. Eighty-nine dates were analyzed from the tell and flat settlement of the site in 2010 (*155*). Sixty-seven dates analyzed by the Debrecen lab derive from the 1989-2002

campaign, 60 from the tell, and 16 from the flat settlement (used to calculate the house Gini value). The authors build models, exclude outliers, and calculate spans, resulting in four phases. Using sequence calibration and separate phase modeling, the use span of the tell is estimated at 371, and 396 years, respectively (*155: 371*). The horizontal settlement has a calculated span of 239 years (mean value) from the Debrecen lab, but the Vienna (AMS) lab dates (from different features and not on charcoal) indicate a different span, earlier in time–the dates are therefore not easily pooled. The ditch system is calculated separately, with a span mean of 79 years, but overlaps with other phases without a break in occupation. No definitive conclusion is reached by the authors, but the consensus based on the current data suggests a summed span of close to 400 years. In our model, we sum all dates, except a Late Bronze Age Date (Deb-2219) and the seven dates (*155: 364-6*) considered to be outside of the tell's inner stratigraphy or strongly deviate from the expected range (Deb 1711, Deb 1713, Deb 1714, Deb 1936, Deb-1772, Deb-2522 and Deb-3232). We end up with a comparable span of 355 years.

Rogoza near Maribor. The relative ceramic chronology and (unmodelled) absolute dating of 11 dates from the site place it in use within about 200-250 years, longer than our mean of 110 but within the 2 standard deviations of our calculation (*156: 73*).

Sântana-Cetatea Veche. Thirteen dates are available that parse the three enclosures into roughly two phases (*157*). The oldest enclosure dates to the 15th century but overlaps with the later enclosures, which are contemporary and date to the later 15th and earlier 14th centuries. The authors exclude a burial from the modeling of the site feature dating, but it falls within the range of enclosure use, ca. 150 years, compatible with our mean span of 155 years.

Szederkény-Kukorica-dűlő. Thirty-nine dates are available for the site, and all are used here (*123*). Their most plausible model suggests a use span of 120-155 years (68%), comparable with our value of 155.

Szeghalom-Kovácshalom. There are 12 dates from the site (*28*). Eleven are Middle and Late Neolithic (ca. 5000-4900, and one is a slight outlier ECA date (UGAMS-38414). Intensive surface collection and excavation at the site indicates that it could have been longer lived, beginning the Szakálhát, around 5300 BC, with a short break before the first Early Copper Age date (excluded).

Szemely-Irtás. The site has a number of houses excavated, but all of the dates come from human burials, so no models have been provided (*158*). All are included.

Tiszalúc-Sarkad. There are six Copper Age dates within 100 years (*135*), and a Late Neolithic date (Bln-2675) which is excluded (*159*).

Tolna-Mözs. The site has 13 dates, modelled as a single phase (*160*) with a span of 45-245 years, at one standard deviation. We exclude one outlier (SUERC-100837) but still arrive at a similar estimate, a mean of 130 years.

Túrkeve-Terehalom. There are 32 dates modeled using the stratigraphic sequence of the tell (*161*). Altogether the model suggests an occupation ca. 1950-1675 BC, or 275 years, comparable to the mean value of 255 calculated here.

Uivar. There are 182 dates, modeled and discussed by Bayliss et al. (*141*) as part of the 'Times of our Lives' project. The earliest ditch dates to the lowest level (5) on the tell, the Vinča B/Szakálhát period (*162: 552*), beginning ca. 5200 BC. They model the end of the Neolithic occupation ca. 4600 BC. They do not include any dates from the Eneolithic Foeni-Petreşti culture, which follows the latest Neolithic levels at the tell, but there is a 300-year gap between level (1) and the Tiszapolgár. We exclude one very early date (Poz- 18968) and dates after the gap (Hd - 22711 and later). Our calculated mean of 900 years is longer than their span.

Versend-Gilencsa. The site has 68 dates and was modelled in different ways using the stratigraphy of the site; replicate dates have been combined before modeling (*163*). Like Jakucs et al, we remove three post-Neolithic outliers, and some stratigraphic deviants (UBA-22596, UBA-22602, and SUERC-58578). The burials start earlier and last longer, in contrast to the settlement site. The excavators suggest the site was likely short lived, lasting only a few decades, but the occupation overlaps with a challenging part of the radiocarbon curve. For this reason, the mean we calculate (205+/40 years) likely doesn't correspond to their understanding of the site chronology.

Vésztő-Bikeri / Körösladány-Bikeri. These two sites, only meters away from each other, are treated as one because they represent continuous occupation. Though modeled by Yerkes (*139*), the most comprehensive analysis of the Bikeri sites is found in the site monograph (*164*). Twenty-two dates are known from Vésztő, which the authors parse into three phases, each with internal breaks. of ca. 200 years. Based on stratigraphic data, the ditch and palisade system were constructed in the first phase. No dates are available from the ditches in that phase, but burned daub in the foundation trenches of houses in the second phase suggest the houses were leveled and rebuilt into what ended up being most archaeologically visible and dated, essentially the end of nearby Late Neolithic sites (*164: 166-167*). Fifteen dates come from the second phase, to which most features excavated on the site date. Four dates during the third phase of the site's use are not associated with residential activities, but kilns and child burials are probably associated with the use of Körösladány-Bikeri across the stream.

There are ten dates for this site adjacent to Körösladány, which fall into an additional two settlement phases, the first of which co-occurs with the second phase at Vésztő. The second phase at the site expanded its size, with additional ditch building, when the Vésztő site was no longer in use. Our estimate of 280 years for both sites is consistent with their modeling.

Vésztő-Mágor. The enclosure dates to the Late Neolithic, and there is a break between it and the Early Copper Age strata. There is also a gap between the ECA and the Middle Bronze Age. The site's published Neolithic dates have been modeled in combination with other sites, but not at the site level (*139*). Four older LN dates are currently published (*159*), and we add an additional two previously unpublished here.

Vinkovci-Sopot. Dates from two different labs show a slightly different pictures of the occupations timeframe for the site (*165*). The dates (unmodelled) fall into three continuous

stratigraphic phases across almost a millennium. We exclude three early Neolithic dates (Beta-251909, Beta-251910, Beta-251911). Our mean estimate of 985 years corresponds well to the estimate from Krznarić-Škrivanko (2015).

Vlkas 'Do hulského chotára'. We publish here for the first time twelve dates from the LPC, and three from the site which are Late Bronze Age (and excluded).

Vráble 'Véľke Lehemby'. Dating of three neighborhoods of houses at the site indicates they were contemporary, lasting 220-290 years (68%) (*166*). Burial contexts date somewhat later at the site, as the settlement was going into disuse. For this reason, our model estimated mean site use is 440 years, longer than the settlement alone.

<u>Social cohesion site list</u>

The calculation of ditch volume can be used across the Carpathian Basin in prehistory due to the nature of the geology and practice of digging ditches since the Middle Neolithic. It is nonetheless an imperfect proxy and does not incorporate other time and energy measures such as tree felling, palisade building, preparing of ramparts, or transporting heavy stone slabs for building defenses. Very few ditches have been excavated in their entirety. Most often, the outlines and extent of ditches can be seen in aerial imagery or magnetometry data. We included only sites where most of the ditch could be confidently inferred based on a combination of excavation, coring, remote sensing, or geometric regularity. We did not include sites such as Parța (Romania), which had very little of the ditches excavated, and the extent of them around the site could not be confidently assumed.

Many sites have more than one ditch, but their number and sizes usually preclude investigating them all. As such, in this measure we only report on the ditches tested and for which the original investigators were confident they date to the period in question. Consequently, the measure is almost always a minimum volume value. It also means that the more ditches there are at a site, the less likely they were all investigated, and therefore the more extreme the underrepresentation of larger values. If additional ditches were identifiable their length was measured even if their width and depth were not. This value was used to illustrate the range not calculated into given sites (see table S2, fig. S1).

Ditch systems were dug over an unspecified amount of time. These periods tend to be over a few hundred years, the length of most relative chronological time blocks such as "the Late Neolithic". Even when time frames are long (such as 500 years) we expect that these ditch systems required redigging and maintenance which lessens the effect of longer used ditches on the power of the metric.

The depth of a ditch or palisade is generally measured from the surface, which includes the ploughzone. Nonetheless, not all excavators report exactly how depth was measured, potentially introducing a small amount of error to some values. If there are significant depressions still visible on the modern surface, these are added to the calculation. For example, If there is a 2 m depression between an enclosed site and outside the ditch, this 2 m will be added on to whatever the depth of a 3 m ditch, for a total of 5 m. When a range is given for the dimensions of a feature (e.g. 4-5 m wide), we calculate an average.

We calculate volume using the formula (top width x depth x length) / 2. The raw data can be viewed in Data S1 (Ditches). We've noted the shape of the ditches when known, but not modified the calculation, as the differences would be marginal.

Aba-Belsőbárándpuszta-Bolondvár. The site is created by a ditch 25 m wide, and 2 m deep, running east-west dividing the site in two. In the smallest artificial island there is a small circle with a 6 m wide, 0.5 deep ditch (*167: 7*). The slopes to the west, south, east, and north seem to be natural.

Balatonszárszó-Kis-erdei-dűlő (Copper Age). Only one ditch has been excavated. It is 3 m wide and 0.8 m deep (*168*).

Bogács-Pazsagpuszta. There are two ditches at the site (*169: 155-156*). The inner ditch is not visible in the magnetometry data, and so the extent is unknown. The outer ditch is semi-circular and presumably extends across the plateau to the break in slope in the north and south. Ditch 2 is 10-15 m wide, and 1.4 m deep, according to cores taken in 2016.

Borsodivánka-Marhajárás. The site is enclosed by a single ditch that is visible in the topographic data and evidenced by coring north and south of the tell (*169: 163-164, 169*). The ditch connects the Eger and Rimar rivers, setting it apart from the open settlement. The width is 13-19 m according to the surface depression and coring, and is at least 3 m in depth, with fluvial sediments interspersed and an additional 1 m depression on the surface.

Branč-Helyföldek (Copper Age). The site consists of a settlement and some burials of the Copper Age Ludanice and Brodzany-Nitra group, as well as of a burial ground and some features of the Bronze Age Nitra to Mad'arovce culture. Our analysis refers to the Copper Age settlement features which have been published by J. Vladár and J. Lichardus (*109: 313ff*). The settlement was surrounded by a ditch of 2 m depth and 6 to 7 m width and a wooden palisade. The full extent of this fortification could not be investigated.

Carei-Bobald. There are possibly two ditches, but definitely one. The excavator reports it as 16-17 m wide and 6.5 m deep (*170: 169*), though Kienlin reports it as 12-14 m (*171: 100*)—we use Nemeti's values. There is magnetometry data for the ditches, but only the inner ditch is measured.

Cornești-Iarcuri. The site has four enclosures, but each enclosure includes two ditches. Locations of excavation are illustrated in (*48: 2018*). Enclosure 1 (the innermost) has two ditches 10 meters apart—the outer ditch (AU1) is 7 m wide and 2.1 m deep, while the inner ditch (AU3) is 11 m wide and 4.7 m deep. Enclosure IV has a width of 10 m. A gateway was excavated with a ditch 1.7 m deep and 9.2 m wide.

Csanádpalota-Földvár. The site has four concentric enclosures, with 29 component ditches excavated (*127*). There are two types. The first group belong to the enclosure system and are U or V-shaped cross sections, 2-3 m deep and 4-7 m wide. Feature 23, for example, part of Enclosure 3, is 6-7 m wide, and 1.5-2 m deep running straight in a N-S direction. Other ditches are narrow and shallower and do not fit into the enclosure system, often cross cutting other

ditches. These are not included in the calculation here. The innermost ditches were nearly 3 m deep with V shaped cross sections similar to the others. There are 10 ^{14}C dates from the sites, 9 from the BA. They date between 1430 and 1120 BC.

Feudvar near Mošorin. The defensive ditch is dated to the Early Bronze Age. Drillings and geomagnetic survey indicate the ditch was up to 30 m wide and more than 5 m deep (*172: 19, 173*).

Füzesabony-Pusztaszikszó. There are two concentric palisades, with about 25% of the circumferences excavated. Depth and width values can be found in (*174*), with a good plan and reconstruction for the site published in (*25: 301*).

Gelej-Pincehát (Pincehalom). The central part of the site was likely enclosed by a ditch but it is heavily disturbed and largely covered by trees (*169: 189-190*). This inner ditch is ca. 10-14 m wide and cored to 4 m deep, with fluvial sediments interspersed with human activity.

Gorjani Topole. Three enclosures discovered using Google Earth surround the site (*175*). Multiple additional ditches were discovered with subsequent magnetometer survey, but their temporal association is still unclear. Testing of the innermost three enclosures revealed an outer ditch of 2 m in depth. This, and the associated palisade and inner ditch, are radiocarbon dated to the Sopot period.

Gradište Iđoš. There are four enclosures (*111: 295*). The first includes a double ditch, and an upstanding rampart. The second, identified by magnetometer survey, connects the Grcka dn Berculja streams. The third was identified in Google Earth imagery. A fourth links up to both channels and marshy land. No details are published for Enclosure I, although it was excavated in T16. The 'ancillary ditch 2' is a U-shaped ditch and has a width of 6.1 m and depth of 2.95 m up to the modern surface level. Ditch 3 is 2.35 m wide and 1.95 m deep (including topsoil). The ^{14}C data from the ditches indicate all LBA dates.

Hajdúböszörmény-Pródi halom. There are several concentric circular magnetic anomalies indicating ditches and palisades at this site (*176: 174*). The ditch excavated in 2007 is 10 m wide and 1.7-2.5 m in depth or lower (average depth is 2.1m), though digging lower than this was not possible due to the high water table.

Hódmezővásárhely-Gorzsa. Drillings indicate a V-shaped ditch 3-4 m deep, 5 m wide and dated from the earliest establishment of the site (*177: 145-146*). There is also a palisade trench (U-shaped in profile) 1 m deep and 0.5 m wide, just 3 m inside the ditch.

Hódmezővásárhely-Kökénydomb. The ditches at this site were identified using Google Earth and then confirmed with magnetometry (*178*). There are at least 7 ditches, two of which have been cored. The innermost cored ditch (Core B1) shows that it is 1.7 deep and the outer ditch (Core B9) is 2.5 deep. The width of these ditches can be estimated from the magnetometry plan (no scale on p. 266, but the trench figure has a scale on p. 259).

Kajászó-Várdomb. An artificial ditch 1.5 m deep encloses the site on the north and NE sides (Horváth et al. 2001:10). The width is provided in Horváth et al (2001), Plate VI. There are additional measurements of depressions noted, but they appear to be natural.

Kakucs-Turján. There are three ditches visible in the magnetometry data (Pető (*179: 27*). The innermost and middle ditch are 4 m wide and 3-3.5 m deep. The narrower, northern ditch has not been cored or excavated.

Košice-Barca. The ditch around the site is 18 m wide and 2.5 m deep (Vladár (*180: 277*). It appears it would extend around the whole site, or at least the west, south and east. There is no scale in the original publication but one is provided in (*181: 115*). The ditch likely was built across two or more Bronze Age phases.

Lovasberény-Mihályvár. Traces of the ditch are found on the west side, 1.5 m deep, with a width provided in (*167: 12*). Another ditch may have been present at the time of use, but the description is not very clear.

Makó-Rákos-Császárvár. Double ditch with internal ditch cored to 1.8-2 m depth, width estimated to 3 m (*182: 141*). Scale generated using Google Earth. No data on the external ditch.

Mezőcsát-Laposhalom. The site has one ditch, 17-21 m wide, and 2.9 m deep according to 2013 coring data (*169: 213*). An additional depression of 0.5 is present on the surface. There is another parallel linear anomaly but it is unknown if it is Bronze Age or later.

Nagykőrös-Földvár. The ditch is identifiable running along the north, east, and south (*183: Fig. 111*). The ditch is 4 m wide and 3 m deep.

Nitriansky Hrádok Zámeček. There are several ditches and enclosures at the site, but the widest and most visible ditch (Ditch III) likely extended around the entire site (*181: 116, 184: 64-69*). It is 1-12 m wide and 4-5 m deep.

Nižná Myšl'a. There is an older ditch and a younger ditch. The older is quite small, but the younger one connected the southern slope to the western slope, probably surrounding the settlement on three sides (*185: 40*). The younger, outer ditch is 25-27 m wide, and 5-6 m deep. A plan with a scale is provided by Jaeger (*186: 105*).

Öcsöd-Kováshalom. The most detailed account of the ditch system at the site is published where the magnetometry data are presented (*150*). There are three ditch systems, and the innermost and outermost were tested in 2020, and 2021, respectively. The inner ditch is 3.25 m wide and 2.95 m deep, in a V-shaped cross section. The outermost ditch is irregular with multiple recuttings, and infillings. A simplified representation of it can be described as 2 ditches. One is 220 cm at the top, 40-50 at the bottom, and a depth of 190-205 cm. Ditch P4 is excluded.

Orosháza-Nagytatársánc. The site enclosures form a semi-circle and end on the linear paleomeander running NE-SW (*187*). An outer ditch is 8 m wide and 1 m deep (0.89-1.37 m). The innermost ditch is 9 m wide and 2.2 m deep. With the depression, it is 4.8 m deep.

Otomani-Cetatea de pământ. A defensive ditch 8 m wide and 2.5-3 m deep was found between the river bank and a row of pillars, though the whole island was not enclosed (*188: 130*). The extent of the ditch can be seen in aerial imagery (*171: 158*), where at least the white areas on the dark cultivated land encircling two thirds of the island represent ditches.

Otomani-Cetăţuie. On the south side there is one ditch, ca. 20 m wide and 4.3 m deep, with another 1-2 m of depth on the surface. The fortification on the northern side has two ditches and a rampart. The inner ditch is 16 m wide and 5.3 m deep, and the outer is 14 m wide and 3.75 m deep (*189*). The approximate locations of the ditches are illustrated in (*190: 50*). For scale in this image, the shortest trench that cuts across the northern ditch is 32 m long.

Pecica-Sanţul Mare. There are likely more than one ditch at the site but only investigations at the largest one have any details published (*153*). The main ditch was likely dug more than 8 m deep. The ditch according to the profile by O'Shea et al. (*153: 108*) is probably 35 m wide. The length of the ditch can be seen in Nicodemus (*191: 135*), which extends from the river on both sides, in an arc, turning the tell into an artificial island.

Pir-Cetate. The ditch is 20 m wide, and 2 m deep (*192: 114*). The depression with the settlement is another two meters, making the ditch around 4 m deep. The length of the ditch, which divides the tell, is 90 m (*171: 135*) though it is also visible in the magnetometer data.

Pişcolt-Ógát. There are two ditches though the outermost is of unknown date. The inner ditch is 10-12 m wide (*171: 109*), and has been cored to over 3 m (*193: 187*). There is magnetometry data for the site showing its extent (*171: 115*).

Polgár-Bosnyákdomb. There is an oval ditch around the settlement, and two excavation blocks were opened over it. Both trenches verified a 2.4 m deep ditch with a V-cross-section likely dug at the establishment of the settlement (*148: 21*). The magnetometry plan depicts the extent of the enclosure, 80% of it visible.

Polgár-Csőszhalom. There is a good summary image in (*194: 836*). The five individual roundel ditches are not given values, but for the excavated ditches, an average of 7 m wide and 4 m deep is given. These values are assigned to those ditches excavated.

Polgár-Nagy Kasziba. Original report published in (*195*), but the data are reproduced in (*196*); in the latter, Fig. 3 includes magnetometry data showing a double enclosure. Raczky and Anders report that the ditch width is 1 m and 0.65 m deep, V-shaped in profile (*25*). Additional ditches are known from excavation, but it is not clear if they extend.

Rozhanovce. The site has a ditch 15-16 m wide and 4 m deep. A plan is published in (*197*).

Sălacea Dealul Vida. There are up to four ditches. A linear ditch appears to be medieval, but the primary, innermost circular Bronze Age ditch investigated is 21-22 m wide (*171: 146, 198: 52*). This ditch was excavated to 7.3-7.55 m in depth (*199: 208*). Magnetometer survey highlighted

ca. 40% of the extent of the ditch (*171: 153*). The dating of two additional outer ditches identified in the survey is unknown.

Sântana-Cetatea Veche. There are three enclosures. Enclosure I is rectangular and has a ditch and palisade. Enclosure II has a ditch and palisade combination. Enclosure III would have worked in combination with II (*157: 195*). Enclosure III has a rampart 27 m long and 2.5 m high, with a palisade, and two ditches in front of it. The first (inner) is 6.5 m wide and 2.48 m deep, and the second (outer) is 7.6 m wide and 2.5 m deep. Enclosure I has material from the Early Copper Age to the end of the Bronze Age in it, and some details are published (*200*), but it is excluded because the dimensions of this feature are not clear.

Soroksár-Várhegy. There is a single ditch measuring 3.6 m wide and 1.67 m deep running on the eastern half of the settlement (*201: 8*).

Spišský Štrvtok. A ditch is found on the eastern flank of the site, 6 m wide and 2 m deep (*202: 286*). There is a map with a scale in (*181: 117*).

Szakáld-Testhalom. The site has two ditches, but the inner one has been largely filled in (*169: 229*). Ditch 2 encloses the tell component of the site, is 17-19 m wide, and 4 m below the surface. A site map with magnetometry data is provided by Kienlin (*169: 235*).

Szarvas 38. There are two enclosures at this site. The inner palisade is 1.5 m wide and 2 m deep. An exterior ditch is U-shaped, 18 m wide and 2.8 m deep (*203: 59*). The reconstructed plan for the enclosures are found in (*204*) and (*25*).

Tard-Tatárdomb. The site has two ditches, though they were unlikely contemporaneous (*169: 237*). The inner and earlier ditch is 8-17 m wide, and 3.9 m deep, according to coring in 2013 (*205*). The outer and later ditch is 4-8 m wide but there is no depth information. The inner ditch visible likely surrounds the tell on the NE side as well (*169: 242*).

Tiszaalpár. A site plan is provided in (*206: 17-18*), where the Bronze Age component is the smaller mound in the southeast of the image. There are two ditches, one to the west and one to the south side, creating a moat joined with the river to the north and east. The ditches are 14 m wide and 2.5 m deep.

Tiszalúc-Sarkad. There is a single ditch for a palisade at this site, much of which was excavated (*207*). The palisade is 0.4 m wide and 1.3 m deep.

Tiszaug-Kisrétpart. There is a single ditch that is likely connected with wetland or the Tisza. The reconstructed area can be found in (*208: 232*). The ditch width is 6-7.5 m and depth 2.6-3 m.

Túrkeve-Terehalom. Magnetometry and coring indicate that the ditch surrounded the entire tell (*209: 36-37*). Coring indicates it was 20 m wide, and 4-4.5 m deep. Google Earth imagery, where the ditches can be seen as white colored anomalies, was used to provide a scale.

Uivar. Several ditches were identified in magnetometry data—the most recent and comprehensive discussion of them is in Chapter 7 of the Uivar monograph (*162*), with the latest interpretation in Fig. 2-2-05. There are two inner ditches and an additional four in the outer zone. The excavators list a combined ditch length of at least 1815 m around the tell, and 4240 m for the outer zone, dug over the course of five centuries (*162: 562*). They estimate a ditch volume of more than 30,000 m^3 combined. Our calculations use their ditch lengths, and most lengths and widths, but slightly different assumptions (e.g. only calculating a recut of ditch D5/D8 for consistency with other sites, and taking some averages), yielding a total of 27070 m^3.
The inner two ditches (D1/1043, width 5 m, depth 4 m, and D2/1029, width 2.6 m, depth 2.2 m) were in Block IV. The third (D4) is in block VII (w: 3.0 m, d:3.8 m), the fourth (D5/D8) is in VIII (w: 4.4 m, d: 2.5 m, and appears to have been recut), the fifth (D6/D9) is in Block IX (w:4.5 m; d: 2.7 m), the sixth (D10) is in Block X (w:3.5m: d:2 m), and the last one was not tested, but might be in Block VI (w:6 m, d: 2.5m.).

Végegyháza-Zsibrik-domb. There are several ditches notable in magnetometry data (*210*). The triple ditch ranges between 2 and 4 m wide, 1.8 and 2 m deep, with V-shaped cross sections. These values are averaged and assigned to each of the three ditches, which all go around the site.

Vésztő-Bikeri / Körösladány-Bikeri. Bikeri consists of two sites next to each other, which were used sequentially (see above). This Vésztő site has two enclosures, a wide outer ditch and a shallow palisade. The two can be seen in (*211: 79*), though the western and southern components of them have eroded away. The innermost trench (F20) is a u-shaped palisade and the outermost F 22/F 66 is a V- or trapezoidal-shaped ditch. The inner palisade varies between 0.40 and 0.8 m in width, and extends about 1.6 m below the surface. The outer ditch is up to 1.5 m in places (*212: 125*).

The adjacent Körösladány site has a triple enclosure system (*211: 79-80*). The inner ditch is 1.7 m deep, 0.5 m wide and U-shaped for a palisade. The middle ditch is trapezoidal in cross-section, 3.8 m wide, and extended 2.2 m below the surface (*212: 125*). The outer ditch is 2-2.3 m wide, trapezoidal in cross-section and 1.6-1.8 m below the modern surface.

Vésztő-Mágor. Several ditches are visible in the magnetometry data of the site, most of them probably in clusters (*211: 83, 213*). One ditch cluster, anomaly M6, was excavated and revealed three ditches. An inner ditch or ditch cluster (anomaly M7) is unexcavated, as is a third ditch or ditch cluster north of M5.

Vinkovci-Sopot. Geophysical survey revealed the extent of two ditches circling the settlement, and excavations of the ditches demonstrates they are 6 m in width, and 3 m in depth (Krznarić-Škrivanko 2015).

Vráble (Fidvar). There are three ditches (A, B and C) (*214: 112*). Ceramics from Ditch A date it to the Únětice, Hatvan, Early and Late Mad'arovce phases, but probably was excavated earlier in the sequence (*214: 117*). It is 3.25 m deep (*215: 467*). Ditch B was also excavated, and is approximately 4 m deep with a V-shape, the width was estimated at 22 m. Ditch B is the last phase of the settlement. Ditch C is the maximal extent and was the classical phase of the site. It has been explored using drillings, indicating a depth of 3 m.

Vráble 'Véľke Lehemby'. Two ditches identified in magnetometer data surround the settlement (*216: Fig. 2.1.5*). Both were excavated, with the inner ditch 1 m wide and 0.8 deep, and the outer 3.5 m wide and 1.5 m deep.

<u>Oxcal Script used to calculate Apogee</u>

```
Plot()

{
 Sequence("Alsónyék")
 {
  Boundary("Start Alsónyék ");
  Phase("Phase Alsónyék ")
  {
   R_Date("OxA-30357",6317,32);
   R_Date("SUERC-58484",6307,33);
   R_Date("OxA-30356",6306,32);
   R_Date("OxA-30355",6305,33);
   R_Date("SUERC-58485",6305,34);
   R_Date("SUERC-57544",6267,35);
   R_Date("SUERC-51468",6247,33);
   R_Date("SUERC-57548",6245,34);
   R_Date("SUERC-51459",6244,31);
   R_Date("SUERC-52848",6240,30);
   R_Date("SUERC-51462",6232,32);
   R_Date("OxA-30432",6230,35);
   R_Date("OxA-X-2587- 14",6230,35);
   R_Date("SUERC-51460",6220,30);
   R_Date("Poz-68720",6180,40);
   R_Date("SUERC-57543",6173,34);
   R_Date("Poz-68719",6160,40);
   R_Date("SUERC-51464",6160,30);
   R_Date("SUERC-51461",6132,33);
   R_Date("Poz-68349",6130,35);
   R_Date("Poz-67495",6120,40);
   R_Date("SUERC-51463",6056,31);
   R_Date("Poz-68350",6010,40);
   R_Date("Poz-67767",6000,50);
   R_Date("SUERC-52797",5931,33);
   R_Date("OxA-27582",5907,31);
   R_Date("SUERC-52832",5902,32);
   R_Date("MAMS-20649",5861,18);
   R_Date("SUERC-52803",5860,30);
   R_Date("MAMS-20669",5857,24);
   R_Date("SUERC-52812",5849,31);
   R_Date("OxA-27567",5847,36);
```

```
R_Date("MAMS-20651",5838,19);
R_Date("MAMS-20648",5834,18);
R_Date("SUERC-52804",5833,32);
R_Date("SUERC-52814",5832,32);
R_Date("SUERC-52805",5829,32);
R_Date("MAMS-20653",5828,19);
R_Date("MAMS-20673",5824,24);
R_Date("MAMS-20670",5820,27);
R_Date("SUERC-52853",5815,28);
R_Date("MAMS-20671",5815,23);
R_Date("SUERC-53379",5813,31);
R_Date("SUERC-52795",5805,33);
R_Date("MAMS-20650",5801,18);
R_Date("MAMS-20656",5800,19);
R_Date("SUERC-52823",5798,32);
R_Date("SUERC-52806",5790,28);
R_Date("MAMS-20652",5790,19);
R_Date("MAMS-20676",5787,25);
R_Date("SUERC-52827",5779,32);
R_Date("SUERC-52824",5778,33);
R_Date("SUERC-52807",5778,31);
R_Date("SUERC-52843",5775,29);
R_Date("SUERC-52828",5775,30);
R_Date("SUERC-52808",5774,29);
R_Date("MAMS-20678",5770,26);
R_Date("MAMS-20672",5766,23);
R_Date("SUERC-52822",5765,30);
R_Date("MAMS-20667",5764,20);
R_Date("SUERC-52825",5764,32);
R_Date("SUERC-52802",5757,32);
R_Date("SUERC-52826",5753,33);
R_Date("MAMS-20658",5752,18);
R_Date("MAMS-20664",5744,24);
R_Date("SUERC-52817",5744,32);
R_Date("MAMS-20677",5744,25);
R_Date("MAMS-20662",5735,24);
R_Date("SUERC-52815",5729,32);
R_Date("MAMS-20666",5728,23);
R_Date("MAMS-20668",5727,18);
R_Date("MAMS-20663",5698,25);
R_Date("MAMS-20674",5696,24);
R_Date("SUERC-53036",5695,27);
R_Date("SUERC-52847",5686,31);
R_Date("SUERC-52846",5682,30);
R_Date("SUERC-52838",5676,30);
R_Date("MAMS-20659",5638,24);
```

```
   R_Date("SUERC-52845",5545,31);
  };
  Boundary("End Alsónyék");
  Span();
 };
};


{
 Sequence("Balatonszárszó-Kis-erdei-dűlő")
 {
  Boundary("Start Balatonszárszó-Kis-erdei-dűlő");
  Phase("Phase Balatonszárszó-Kis-erdei-dűlő")
  {
   R_Date("OxA-13655",6339,32);
   R_Date("OxA-13651",6330,33);
   R_Date("OxA-13650",6292,33);
   R_Date("OxA-13654",6280,32);
   R_Date("SUERC-78058",6261,29);
   R_Date("SUERC-78052",6240,29);
   R_Date("OxA-36840",6235,34);
   R_Date("OxA-13653",6234,33);
   R_Date("OxA-13642",6229,35);
   R_Date("OxA-13783",6227,38);
   R_Date("OxA-13640",6221,32);
   R_Date("SUERC-78056",6221,29);
   R_Date("OxA-13795",6216,36);
   R_Date("OxA-13799",6161,35);
   R_Date("SUERC-78057",6143,29);
  };
  Boundary("End Balatonszárszó-Kis-erdei-dűlő");
  Span();
 };
};


{
 Sequence("Belovode")
 {
  Boundary("Start Belovode");
  Phase("Phase Belovode")
  {
```

```
  R_Date("MAMS-22078",6422,23);
  R_Date("MAMS-22073",6389,23);
  R_Date("MAMS-22077",6365,25);
  R_Date("OxA-14684",6354,36);
  R_Date("MAMS-23379",6305,27);
  R_Date("OxA-14680",6268,37);
  R_Date("OxA-14683",6258,36);
  R_Date("OxA-14681",6182,37);
  R_Date("MAMS-22074",6179,23);
  R_Date("MAMS-22071",6151,28);
  R_Date("OxA-14700",6145,40);
  R_Date("MAMS-22076",6143,27);
  R_Date("OxA-14679",6090,36);
  R_Date("MAMS-23381",6038,27);
  R_Date("MAMS-22075",6026,23);
  R_Date("OxA-14682",6025,36);
  R_Date("MAMS-22079",5923,25);
  R_Date("MAMS-23378",5922,26);
  R_Date("MAMS-23377",5839,26);
  R_Date("MAMS-23376",5829,24);
  R_Date("MAMS-23380",5808,26);
  R_Date("OxA-14628",5800,36);
  R_Date("MAMS-22067",5741,23);
  R_Date("MAMS-22070",5735,26);
  R_Date("MAMS-22069",5669,25);
 };
 Boundary("End Belovode");
 Span();
};
};
```

```
{
 Sequence("Borđoš")
 {
  Boundary("Start Borđoš");
  Phase("Phase Borđoš")
  {
   R_Date(6270,80);
   R_Date(6115,35);
   R_Date(6110,90);
   R_Date(6110,40);
   R_Date(6110,40);
```

```
  R_Date(6100,40);
  R_Date(6060,60);
  R_Date(6055,35);
  R_Date(6040,40);
  R_Date(6020,40);
  R_Date(6010,35);
  R_Date(6000,35);
  R_Date(5990,40);
  R_Date(5980,40);
  R_Date(5955,35);
  R_Date(5950,40);
  R_Date(5920,40);
  R_Date(5910,40);
  R_Date(5900,40);
  R_Date(5900,35);
  R_Date(5890,40);
  R_Date(5880,40);
  R_Date(5875,35);
  R_Date(5860,120);
  R_Date(5860,40);
  R_Date(5850,35);
  R_Date(5850,35);
  R_Date(5840,40);
  R_Date(5840,35);
  R_Date(5830,40);
  R_Date(5815,35);
  R_Date(5800,40);
  R_Date(5795,30);
  R_Date(5740,35);
  R_Date(5670,40);
 };
 Boundary("End Borđoš");
 Span();
};
};


{
 Sequence("Brunn am Gebirge")
 {
 Boundary("Start Brunn am Gebirge");
 Phase("Phase Brunn am Gebirge")
  {
  R_Date("VERA-1795",6891,38);
  R_Date("Poz-66177",6870,40);
  R_Date("ETH-11148",6785,75);
```

```
R_Date("Ki-13615",6785,60);
R_Date("Ki-13610",6755,60);
R_Date("VERA-1808",6747,38);
R_Date("Ki-13612",6700,60);
R_Date("Poz-66176",6680,40);
R_Date("ETH-11141",6660,75);
R_Date("VERA-1804",6657,38);
R_Date("ETH-13538",6605,85);
R_Date("ETH-13537",6565,85);
R_Date("VERA-1800",6521,30);
R_Date("ETH-11122",6520,55);
R_Date("ETH-11127",6520,50);
R_Date("Poz-91064",6520,40);
R_Date("ETH-11143",6505,75);
R_Date("Ki-13613",6500,60);
R_Date("VERA-1799",6498,34);
R_Date("ETH-11131",6485,80);
R_Date("ETH-11145",6480,70);
R_Date("ETH-11124",6470,55);
R_Date("ETH-11139",6470,75);
R_Date("Poz-91063",6470,50);
R_Date("VERA-1805",6470,33);
R_Date("ETH-14827",6460,70);
R_Date("VERA-1797",6453,29);
R_Date("Ki-13614",6435,60);
R_Date("Poz-79948",6430,40);
R_Date("VERA-202",6429,31);
R_Date("VERA-192",6410,60);
R_Date("Poz-66175",6410,50);
R_Date("VERA-201",6403,30);
R_Date("Poz-79951",6400,40);
R_Date("ETH-11133",6395,75);
R_Date("VERA-199",6394,30);
R_Date("ETH-11138",6390,65);
R_Date("Poz-66180",6390,40);
R_Date("VERA-200",6387,33);
R_Date("VERA-195",6386,31);
R_Date("Poz-79949",6380,40);
R_Date("VERA-193",6372,31);
R_Date("VERA-197",6369,35);
R_Date("VERA-1806",6368,29);
R_Date("ETH-11130",6365,55);
R_Date("ETH-11147",6365,70);
R_Date("ETH-11128",6360,60);
R_Date("ETH-11150",6360,70);
R_Date("VERA-3414",6339,64);
```

```
R_Date("ETH-11149",6335,70);
R_Date("VERA-1802",6333,43);
R_Date("VERA-1798",6330,31);
R_Date("VERA-3415",6330,48);
R_Date("VERA-1813",6327,45);
R_Date("ETH-11134",6325,70);
R_Date("ETH-11132",6320,65);
R_Date("VERA-1886",6318,32);
R_Date("ETH-11146",6315,70);
R_Date("Hd-14234",6315,35);
R_Date("Poz-79952",6300,40);
R_Date("Poz-79944",6300,40);
R_Date("VERA-1796",6298,44);
R_Date("ETH-11137",6285,70);
R_Date("VERA-1885",6282,37);
R_Date("Poz-79931",6280,90);
R_Date("VERA-1811",6280,36);
R_Date("VERA-1810",6278,39);
R_Date("VERA-1812",6270,31);
R_Date("Poz-66179",6270,40);
R_Date("ETH-11121",6265,55);
R_Date("ETH-11129",6265,75);
R_Date("ETH-11140",6265,70);
R_Date("ETH-11123",6260,70);
R_Date("VERA-1801",6257,32);
R_Date("VERA-1803",6256,31);
R_Date("Poz-79946",6255,35);
R_Date("ETH-11125",6235,70);
R_Date("VERA-1887",6227,37);
R_Date("VERA-4797",6224,37);
R_Date("VERA-4798",6223,38);
R_Date("VERA-1889",6217,37);
R_Date("VERA-196",6216,41);
R_Date("Poz- 79932",6210,70);
R_Date("Ki-13611",6205,60);
R_Date("VERA-1807",6200,31);
R_Date("UBA-15997",6191,27);
R_Date("VERA-1809",6184,29);
R_Date("Poz-79945",6180,40);
R_Date("VERA-1892",6174,37);
R_Date("UBA-16000",6167,26);
R_Date("VERA-4799",6162,41);
R_Date("ETH-11126",6150,75);
R_Date("UBA-15999",6143,29);
R_Date("Hd-14396",6135,23);
R_Date("VERA-1893",6129,35);
```

```
  R_Date("VERA-1814",6121,31);
  R_Date("UBA-15998",6105,25);
  R_Date("VERA-3420",6095,45);
  R_Date("VERA-4800",6091,37);
  R_Date("Hd-14199",6060,40);
 };
 Boundary("End Brunn am Gebirge");
 Span();
};
};
```

```
{
 Sequence("Corneşti-Iarcuri")
 {
  Boundary("Start Corneşti-Iarcuri");
  Phase("Phase Corneşti-Iarcuri")
  {
   R_Date("MAMS-30435",3223,18);
   R_Date("MAMS-35578",3204,20);
   R_Date("MAMS-30433",3200,18);
   R_Date("MAMS-35576",3179,18);
   R_Date("MAMS-26692",3170,27);
   R_Date("MAMS-29742",3165,22);
   R_Date("MAMS-29745",3160,21);
   R_Date("MAMS-30434",3138,19);
   R_Date("MAMS-35957",3132,22);
   R_Date("MAMS-35577",3130,19);
   R_Date("MAMS-35580",3128,18);
   R_Date("MAMS-29739",3126,21);
   R_Date("MAMS-30432",3116,24);
   R_Date("BETA-258641",3110,40);
   R_Date("MAMS-30423",3106,18);
   R_Date("Poz-53343",3105,35);
   R_Date("MAMS-29746",3104,21);
   R_Date("MAMS-29741",3102,21);
   R_Date("MAMS-29747",3101,21);
   R_Date("Poz-53345",3100,30);
   R_Date("MAMS-35575",3099,18);
   R_Date("Poz-53344",3095,30);
   R_Date("Poz-53347",3095,35);
   R_Date("Poz-53342",3085,35);
   R_Date("Poz-53352",3085,35);
   R_Date("MAMS-26691",3085,25);
```

```
   R_Date("Poz-53351",3075,35);
   R_Date("MAMS-35955",3071,23);
   R_Date("Poz-45667",3070,30);
   R_Date("BETA-258640",3060,40);
   R_Date("Poz-53346",3050,30);
   R_Date("MAMS-35579",3044,19);
   R_Date("BETA-258642",3040,40);
   R_Date("Poz-53349",3020,35);
   R_Date("MAMS-30427",2987,18);
   R_Date("BETA-258645",2970,40);
   R_Date("MAMS-30436",2970,18);
   R_Date("MAMS-30426",2947,18);
   R_Date("MAMS-29740",2938,20);
  };
  Boundary("End Corneşti-Iarcuri");
  Span();
 };
};
```

```
{
 Sequence("Csanádpalota-földvár")
 {
  Boundary("Start Csanádpalota-földvár");
  Phase("Phase Csanádpalota-földvár")
  {
   R_Date("DeA-8211",3119,24);
   R_Date("DeA-8209",3074,25);
   R_Date("DeA-3485",3037,35);
   R_Date("DeA-3484",3025,36);
   R_Date("DeA-3483",3012,35);
   R_Date("DeA-8296",3009,27);
   R_Date("DeA-3470",2976,35);
   R_Date("DeA-8210",2964,24);
  };
  Boundary("End Csanádpalota-földvár");
  Span();
 };
};
```

```
{
 Sequence("Divostin I")
 {
  Boundary("Start Divostin I");
  Phase("Phase Divostin I")
  {
   R_Date("Bln-866a",7200,100);
   R_Date("Bln-899",7200,100);
   R_Date("BRAMS-2402",7160,28);
   R_Date("Bln-826",7120,100);
   R_Date("Bln-866/866a/931",7104,58);
   R_Date("Bln-823",7080,180);
   R_Date("Bln-866",7060,100);
   R_Date("Bln-931",7050,100);
   R_Date("Bln-862",6995,100);
   R_Date("Bln-824",6970,100);
   R_Date("Bln-896",6950,100);
   R_Date("BM-573",6935,98);
   R_Date("Bln-827",6910,100);
   R_Date("Z-1505",6900,150);
  };
  Boundary("End Divositn I");
  Span();
 };
};


{
 Sequence("Divostin II")
 {
  Boundary("Start Divostin II");
  Phase("Phase Divostin II")
  {
   R_Date("Z-336B",6052,90);
   R_Date("Bln-865",6020,100);
   R_Date("Z-336a",6005,93);
   R_Date("Bln-865a",5965,100);
   R_Date("Bln-898",5860,100);
   R_Date("Bln-863",5825,100);
   R_Date("OxA-14693",5811,34);
   R_Date("OxA-14705",5810,36);
   R_Date("OxA-14694",5775,39);
  };
  Boundary("End Divostin II");
```

```
  Span();
 };
};


{
 Sequence("Drenovac")
 {
  Boundary("Start Drenovac");
  Phase("Phase Drenovac")
  {
   R_Date("BRAMS-2242",6354,27);
   R_Date("BRAMS-2241",6302,27);
   R_Date("SUERC-54884",6274,36);
   R_Date("BRAMS-2237",6243,27);
   R_Date("BRAMS-2243",6226,27);
   R_Date("BRAMS-2238",6120,27);
   R_Date("BRAMS-2240",6110,27);
   R_Date("SUERC-54886",6049,36);
   R_Date("SUERC-54885",5882,36);
  };
  Boundary("End Drenovac");
  Span();
 };
};


{
 Sequence("Feudvar")
 {
  Boundary("Start Feudvar");
  Phase("Phase Feudvar")
  {
   R_Date("Bln-4142",3445,50);
   R_Date("Bln-4264",3420,50);
   R_Date("Bln-4267",3385,50);
   R_Date("Bln-4266",3365,70);
   R_Date("Bln-4140",3290,60);
   R_Date("Bln-4268",3255,50);
   R_Date("Bln-4270",3255,50);
   R_Date("Bln-4161",3245,40);
   R_Date("Bln-4131",3235,40);
   R_Date("Bln-4160",3230,50);
   R_Date("Bln-4135",3160,70);
  };
```

```
  Boundary("End Feudvar");
  Span();
 };
};


{
 Sequence("Füzesabony-Gubakút")
 {
  Boundary("Start Füzesabony-Gubakút");
  Phase("Phase Füzesabony-Gubakút")
  {
   R_Date("Fg-135",6660,55);
   R_Date("Deb-5906",6600,55);
   R_Date("Deb-5882",6550,100);
   R_Date("Deb-5746",6490,60);
   R_Date("Deb-13042",6489,40);
   R_Date("Deb-5943",6485,70);
   R_Date("Deb-5777",6390,45);
   R_Date("Deb-5900",6360,40);
   R_Date("Deb-5941",6345,70);
   R_Date("Deb-13052",6331,63);
   R_Date("VERA-4236",6325,35);
   R_Date("Deb-5875",6325,75);
   R_Date("VERA-4239",6320,40);
   R_Date("Deb-5740",6320,75);
   R_Date("Deb-5859",6320,65);
   R_Date("Deb-5939",6310,60);
   R_Date("Deb-5757",6300,85);
   R_Date("Deb-5937",6300,65);
   R_Date("VERA-4237",6295,35);
   R_Date("VERA-4240",6295,35);
   R_Date("VERA-4242",6295,40);
   R_Date("VERA-4238",6285,35);
   R_Date("Deb-5773",6285,90);
   R_Date("VERA-4241",6255,40);
   R_Date("Deb-4852",6252,58);
   R_Date("Deb-11092",6250,90);
   R_Date("Deb-5897",6250,30);
   R_Date("Deb-5857",6195,60);
   R_Date("Deb-11892",6110,50);
  };
  Boundary("End Füzesabony-Gubakút");
  Span();
```

```
 };
};

{
 Sequence("Füzesabony-Pusztaszikszó")
 {
  Boundary("Start Füzesabony-Pusztaszikszó");
  Phase("Phase Füzesabony-Pusztaszikszó")
  {
   R_Date("Deb-3385",5720,71);
   R_Date("Deb-3379",5682,57);
   R_Date("Deb-3380",5614,70);
   R_Date("Bln-3926",5590,80);
   R_Date("Bln-3930",5460,70);
   R_Date("Bln-3929",5380,60);
  };
  Boundary("End Füzesabony-Pusztaszikszó");
  Span();
 };
};

{
 Sequence("Gomolava (Vinca)")
 {
  Boundary("Start Gomolava (Vinca)");
  Phase("Phase Gomolava (Vinca)")
  {
   R_Date("GrN-13165",6060,35);
   R_Date("GrN-13093",6040,100);
   R_Date("GrN-13092",6025,35);
   R_Date("OxA-21135",6020,40);
   R_Date("GrN-13163",6015,35);
   R_Date("OxA-21136",6015,40);
   R_Date("GrN-7376",6010,70);
   R_Date("OxA-21137",5985,33);
   R_Date("OxA-21134",5971,37);
   R_Date("OxA-21139",5964,35);
   R_Date("OxA-21138",5946,35);
```

```
  R_Date("GrN-13097",5945,50);
  R_Date("OxA-22339",5944,34);
  R_Date("OxA-21133",5934,40);
  R_Date("GrN-13094",5930,45);
  R_Date("OxA-21140",5930,35);
  R_Date("OxA-14710",5922,36);
  R_Date("GrN-13166",5920,100);
  R_Date("GrN-13161",5895,35);
  R_Date("GrN-13164",5860,70);
  R_Date("OxA-14707",5848,38);
  R_Date("GrN-13162",5835,35);
  R_Date("OxA-14706",5824,37);
  R_Date("GrN-13091",5820,70);
  R_Date("OxA-14709",5773,35);
  R_Date("OxA-14708",5739,35);
  R_Date("GrN-13159",5720,140);
  R_Date("GrN-7374",5715,75);
  R_Date("GrN-13160",5710,60);
 };
 Boundary("End Gomolava (Vinca)");
 Span();
};
};
```

```
{
 Sequence("Gradište Iđoš")
 {
  Boundary("Start Gradište Iđoš");
  Phase("Phase Gradište Iđoš")
  {
   R_Date("KIA-51810",3172,19);
   R_Date("KIA-51804",3132,21);
   R_Date("OxA-38717",3127,22);
   R_Date("OxA-38714",3115,22);
   R_Date("OxA-42403",3083,20);
   R_Date("OxA-42402",3081,20);
   R_Date("OxA-39211",3076,21);
   R_Date("KIA-51807",3069,20);
   R_Date("KIA-51812",3052,20);
   R_Date("OxA-42405",3050,20);
   R_Date("OxA-42404",3045,20);
   R_Date("OxA-38716",3043,22);
```

```
   R_Date("OxA-41398",3042,19);
   R_Date("KIA-51814",3024,28);
   R_Date("OxA-38590",3021,19);
   R_Date("OxA-39297",3007,21);
   R_Date("KIA-51811",2985,27);
  };
  Boundary("End Gradište Iđoš");
  Span();
 };
};


{
 Sequence("Hódmezővásárhely-Gorzsa")
 {
  Boundary("Start Hódmezővásárhely-Gorzsa");
  Phase("Phase Hódmezővásárhely-Gorzsa")
  {
   R_Date("Poz-7969",6270,40);
   R_Date("Poz-7973",6270,40);
   R_Date("Poz-7971",6230,40);
   R_Date("Poz-7972",6160,40);
   R_Date("Poz-7967",6110,35);
   R_Date("Poz-7968",6110,40);
   R_Date("Deb-1202",6050,60);
   R_Date("Deb-1187",5990,60);
   R_Date("Fra-108",5970,100);
   R_Date("Fra-95",5970,100);
   R_Date("Poz-7695",5960,40);
   R_Date("Fra-114",5910,100);
   R_Date("Deb-1191",5910,60);
   R_Date("Deb-1240",5900,60);
   R_Date("Deb-1387",5900,60);
   R_Date("Z-2011",5890,110);
   R_Date("Deb-1173",5880,60);
   R_Date("Deb-1174",5860,60);
   R_Date("Deb-1172",5850,60);
   R_Date("Deb-1386",5840,60);
   R_Date("Deb-1354",5830,60);
   R_Date("Z-2010",5820,110);
   R_Date("Deb-1413",5790,60);
   R_Date("Deb-1384",5780,60);
   R_Date("Deb-1171",5760,60);
   R_Date("Deb-1175",5760,60);
   R_Date("Deb-1238",5750,60);
   R_Date("Fra-77",5670,100);
```

```
   R_Date("Fra-76",5650,110);
   R_Date("Bln-1934",5595,65);
   R_Date("Bln-",5580,100);
  };
  Boundary("End Hódmezővásárhely-Gorzsa");
  Span();
 };
};
```

```
{
 Sequence("Hódmezővásárhely-Kökénydomb")
 {
  Boundary("Start Hódmezővásárhely-Kökénydomb");
  Phase("Phase Hódmezővásárhely-Kökénydomb")
  {
   R_Date("Deb-1263",6190,60);
   R_Date("Deb-1365",6150,60);
   R_Date("Poz-36369",6100,50);
   R_Date("Deb-1412",6100,60);
   R_Date("Deb-1388",6090,60);
   R_Date("Deb-357",5980,200);
   R_Date("Deb-1367",5970,60);
   R_Date("Deb-1513",5900,60);
   R_Date("Bln-515",5890,120);
   R_Date("Deb-1255",5890,60);
   R_Date("Deb-1364",5870,60);
   R_Date("Deb-1411",5850,60);
   R_Date("Deb-1222",5800,60);
  };
  Boundary("End Hódmezővásárhely-Kökénydomb");
  Span();
 };
};
```

```
{
 Sequence("Lepenski Vir")
 {
  Boundary("Start Lepenski Vir");
  Phase("Phase Lepenski Vir")
```

```
{
 R_Date("OxA-26554",7462,44);
 R_Date("KN-405",7430,160);
 R_Date("OxA-26547",7396,40);
 R_Date("OxA-8725",7380,93);
 R_Date("OxA-11698",7374,80);
 R_Date("OxA-11704",7368,75);
 R_Date("Bln-740b",7360,100);
 R_Date("OxA-11700",7353,72);
 R_Date("OxA-11696",7346,57);
 R_Date("OxA-11701",7337,79);
 R_Date("OxA-34519",7333,62);
 R_Date("OxA-X-2176-19",7314,40);
 R_Date("X-2176-19",7314,40);
 R_Date("OxA-11705",7312,79);
 R_Date("OxA-5827",7311,108);
 R_Date("Bln-740a",7310,100);
 R_Date("Z-143",7300,124);
 R_Date("OxA-25214",7289,69);
 R_Date("OxA-16084",7285,37);
 R_Date("OxA-X-2176-18",7285,45);
 R_Date("X-2176-18",7285,45);
 R_Date("OxA-11693",7284,47);
 R_Date("KN-407",7280,160);
 R_Date("OxA-15998",7280,45);
 R_Date("OxA-25213",7269,68);
 R_Date("OxA-11697",7250,59);
 R_Date("OxA-16001",7235,40);
 R_Date("KN-1407",7230,60);
 R_Date("OxA-25090",7226,72);
 R_Date("Bln-738",7225,100);
 R_Date("OxA-16077",7225,40);
 R_Date("OxA-25204",7224,72);
 R_Date("OxA-16081",7219,37);
 R_Date("OxA-11692",7218,81);
 R_Date("KN-1405",7210,55);
 R_Date("KN-406",7210,200);
 R_Date("OxA-27901",7207,35);
 R_Date("OxA-25205",7203,73);
 R_Date("OxA-8618",7200,60);
 R_Date("OxA-16078",7191,40);
 R_Date("OxA-32887",7191,35);
 R_Date("OxA-16005",7190,45);
 R_Date("OxA-16006",7190,40);
 R_Date("UBA-29841",7187,73);
 R_Date("OxA-25211",7179,73);
```

```
R_Date("OxA-32865",7176,36);
R_Date("OxA-16003",7170,40);
R_Date("OxA-25209",7169,52);
R_Date("OxA-16009",7165,40);
R_Date("OxA-16002",7160,40);
R_Date("AA-57779",7157,79);
R_Date("OxA-12979",7157,77);
R_Date("OxA-16075",7157,39);
R_Date("OxA-32886",7156,36);
R_Date("OxA-25210",7155,54);
R_Date("OxA5830",7153,106);
R_Date("OxA-5830",7152,106);
R_Date("AA-63177",7148,77);
R_Date("OxA-16082",7138,37);
R_Date("OxA-16538",7136,37);
R_Date("OxA-11702",7133,75);
R_Date("OxA-32933",7133,37);
R_Date("OxA-16073",7125,40);
R_Date("BA-10653",7123,89);
R_Date("AA-57782",7122,57);
R_Date("OxA-25091",7119,74);
R_Date("OxA-15999",7111,40);
R_Date("OxA-16008",7092,42);
R_Date("OxA-34968",7080,42);
R_Date("OxA-16000",7070,40);
R_Date("AA-57783",7067,55);
R_Date("OxA-16083",7059,36);
R_Date("OxA-25206",7058,37);
R_Date("OxA-16007",7050,40);
R_Date("OxA-25089",7046,71);
R_Date("OxA-16213",7043,37);
R_Date("OxA-16212",7041,35);
R_Date("Bln-653",7040,100);
R_Date("OxA-16079",7037,39);
R_Date("OxA-5828",7032,95);
R_Date("AA-57780",7031,95);
R_Date("OxA-16211",7021,36);
R_Date("OxA-16253",7008,38);
R_Date("OxA-25207",6984,39);
R_Date("Z-115",6984,94);
R_Date("OxA-11695",6982,50);
R_Date("OxA-16010",6980,81);
R_Date("OxA-5831",6979,92);
R_Date("BA-10652",6973,48);
R_Date("UCLA--1407",6970,60);
R_Date("UCLA-1407",6970,60);
```

```
   R_Date("OxA-11694",6942,47);
   R_Date("OxA-16537",6924,37);
   R_Date("Bln-379",6900,100);
   R_Date("Bln-678",6900,100);
   R_Date("BM-379",6900,150);
   R_Date("OxA-25208",6893,45);
   R_Date("Bln-575",6860,100);
   R_Date("Bln-647",6845,100);
   R_Date("AA-58320",6829,53);
   R_Date("Bln-576",6820,100);
   R_Date("Bln-650",6820,100);
   R_Date("P-1598",6814,69);
   R_Date("Bln-649",6800,100);
   R_Date("OxA-5829",6721,93);
   R_Date("AA-58319",6690,54);
   R_Date("Bln-654",6630,100);
   R_Date("Bln-652",6620,100);
   R_Date("Bln-655",6560,100);
   R_Date("KN-1406",6560,120);
  };
  Boundary("End Lepenski Vir");
  Span();
 };
};


{
 Sequence("Mold Im Doppel")
 {
  Boundary("Start Mold Im Doppel");
  Phase("Phase Mold Im Doppel")
  {
   R_Date("VERA 400",6360,60);
   R_Date("VERA 4083",6330,35);
   R_Date("VERA 4080",6280,35);
   R_Date("VERA 4085",6255,40);
   R_Date("VERA 397",6250,70);
   R_Date("VERA 396",6240,45);
   R_Date("VERA 3950",6215,35);
   R_Date("VERA 395",6210,45);
   R_Date("VERA 4082",6200,40);
   R_Date("VERA 3949",6165,40);
   R_Date("VERA 3958",6165,40);
   R_Date("VERA 3959",6165,40);
   R_Date("VERA 4084",6145,35);
   R_Date("VERA 4270",6130,35);
```

```
   R_Date("VERA 3963",6125,40);
   R_Date("VERA 4059",6085,35);
   R_Date("VERA 3951",6060,40);
   R_Date("VERA 3948",6055,40);
   R_Date("VERA 4057",5975,40);
  };
  Boundary("End Mold Im Doppel");
  Span();
 };
};
```

```
{
 Sequence("Öscöd-Kováshalom")
 {
  Boundary("Start Öscöd-Kováshalom");
  Phase("Phase Öscöd-Kováshalom")
  {
   R_Date("Deb-1710",6240,60);
   R_Date("Deb-3314",6225,122);
   R_Date("Bln-3957b",6180,60);
   R_Date("Bln-3965",6170,80);
   R_Date("Deb-1707",6170,40);
   R_Date("Deb-3332",6142,70);
   R_Date("Deb-3325",6127,93);
   R_Date("Deb-1708",6120,40);
   R_Date("Deb-3336",6114,74);
   R_Date("MAMS-56021",6084,23);
   R_Date("Bln-3962",6070,60);
   R_Date("MAMS-56019",6064,23);
   R_Date("Bln-3964",6060,50);
   R_Date("Bln-3957c",6040,50);
   R_Date("Bln-3966",6040,70);
   R_Date("MAMS-56025",6027,23);
   R_Date("MAMS-56015",6022,23);
   R_Date("MAMS-56016",6017,23);
   R_Date("Bln-3957a",6010,100);
   R_Date("MAMS-56023",6003,23);
   R_Date("MAMS-56024",6001,23);
   R_Date("MAMS-56017",5999,23);
   R_Date("Deb-3350",5981,79);
   R_Date("MAMS-56020",5973,23);
   R_Date("MAMS-56022",5959,23);
   R_Date("Bln-3963",5920,50);
```

```
  R_Date("MAMS-56018",5801,24);
  };
 Boundary("End Öscöd-Kováshalom");
 Span();
 };
};


{
 Sequence("Opovo")
 {
  Boundary("Start Opovo");
  Phase("Phase Opovo")
  {
   R_Date("OxA-21124",6036,36);
   R_Date("CAMS-4411",6030,60);
   R_Date("OxA-21131",5992,39);
   R_Date("CAMS-4413",5990,60);
   R_Date("OxA-21123",5978,35);
   R_Date("OxA-21125",5973,35);
   R_Date("OxA-21130",5972,37);
   R_Date("OxA-21126",5966,36);
   R_Date("OxA-21129",5963,34);
   R_Date("OxA-21128",5926,35);
   R_Date("OxA-21127",5921,35);
   R_Date("OxA-21212",5913,30);
   R_Date("OxA-21454",5887,36);
  };
  Boundary("End Opovo");
  Span();
 };
};


{
 Sequence("Parţa")
 {
  Boundary("Start Parţa");
  Phase("Phase Parţa")
  {
   R_Date("GrN-28460",6860,60);
   R_Date("GrN-28877",6800,50);
   R_Date("GrN-28459",6660,60);
   R_Date("Lv-2145",6560,140);
```

```
  R_Date("Lv-2147",6500,130);
  R_Date("Lv-2146",6470,150);
  R_Date("Lv-2143",6340,100);
  R_Date("Lv-2139",6330,140);
  R_Date("Lv-2141",6290,80);
  R_Date("Lv-2142",6240,80);
  R_Date("Lv-2148",6240,70);
  R_Date("Lv-2151",6240,70);
  R_Date("Lv-2138",6160,100);
  R_Date("Lv-2149",6160,90);
  R_Date("Lv-2140",6140,80);
  R_Date("Lv-2144",6100,80);
  R_Date("Lv-2150",6070,90);
 };
 Boundary("End Parţa");
 Span();
};
};



{
 Sequence("Pecica-Şanţul Mare")
 {
  Boundary("Start Pecica-Şanţul Mare");
  Phase("Phase Pecica-Şanţul Mare")
  {
   R_Date("Beta-207591",3600,80);
   R_Date("Beta-207595",3580,40);
   R_Date("Beta-207597",3560,40);
   R_Date("Beta-207589",3550,60);
   R_Date("06-116",3520,40);
   R_Date("Beta-207598",3510,40);
   R_Date("Beta-207602",3510,50);
   R_Date("Beta-207593",3500,40);
   R_Date("Beta-207588",3490,50);
   R_Date("Beta-207600",3490,40);
   R_Date("Beta-207592",3480,40);
   R_Date("Beta-207590",3450,40);
   R_Date("06-133",3450,40);
   R_Date("06-105",3440,40);
   R_Date("06-115",3430,40);
   R_Date("Beta-207596",3410,40);
   R_Date("06-136",3400,40);
   R_Date("Beta-207601",3370,40);
   R_Date("Beta-207603",3360,40);
```

```
   R_Date("06-129",3240,40);
  };
  Boundary("End Pecica-Şanţul Mare");
  Span();
 };
};


{
 Sequence("Polgár-Csőszhalom")
 {
  Boundary("Start Polgár-Csőszhalom");
  Phase("Phase Polgár-Csőszhalom")
  {
   R_Date("Deb-1900",6100,40);
   R_Date("Deb-1942",6060,40);
   R_Date("Deb-9808",6060,60);
   R_Date("VERA-3064",6025,40);
   R_Date("Deb-3292",6024,70);
   R_Date("BM-2321",6020,170);
   R_Date("VERA-3069",6015,35);
   R_Date("VERA-3065",6005,45);
   R_Date("Deb-3236",5984,72);
   R_Date("Deb-3333",5981,37);
   R_Date("Deb-2506",5960,40);
   R_Date("Deb-1965",5950,40);
   R_Date("Deb-2540",5945,40);
   R_Date("Bln-513",5940,100);
   R_Date("Deb-1941",5940,40);
   R_Date("Deb-2554",5930,40);
   R_Date("VERA-3068",5930,35);
   R_Date("VERA-4197",5930,40);
   R_Date("Deb-3308",5929,63);
   R_Date("Deb-3266",5928,65);
   R_Date("Deb-1957",5920,40);
   R_Date("VERA-3062",5915,40);
   R_Date("Deb-3351",5910,70);
   R_Date("VERA-4768",5910,40);
   R_Date("Deb-3277",5906,58);
   R_Date("Deb-2504",5905,40);
   R_Date("Deb-2421",5900,40);
   R_Date("VERA-3061",5895,40);
   R_Date("Bln-510",5875,100);
   R_Date("Deb-3306",5872,58);
```

```
R_Date("Deb-2498",5870,40);
R_Date("Deb-10219",5865,80);
R_Date("Deb-1904",5860,40);
R_Date("Deb-2505",5860,40);
R_Date("Deb-3226",5855,57);
R_Date("Deb-3261",5855,73);
R_Date("VERA-3067",5855,30);
R_Date("Deb-2496",5850,40);
R_Date("Deb-3237",5848,66);
R_Date("Deb-2497",5845,40);
R_Date("GrN-1993",5845,60);
R_Date("Deb-3229",5842,58);
R_Date("Deb-2541",5840,40);
R_Date("Deb-10198",5835,50);
R_Date("Deb-2523",5830,40);
R_Date("VERA-3060",5830,35);
R_Date("Deb-10197",5825,40);
R_Date("Deb-2528",5825,40);
R_Date("Deb-10107",5810,50);
R_Date("Deb-2520",5810,40);
R_Date("Deb-2501",5805,40);
R_Date("Deb-1773",5800,40);
R_Date("Deb-1907",5800,40);
R_Date("Deb-2512",5800,40);
R_Date("Deb-2536",5795,40);
R_Date("Deb-1762",5790,40);
R_Date("Deb-1902",5790,40);
R_Date("Deb-2533",5790,40);
R_Date("Deb-2514",5780,40);
R_Date("Deb-3311",5780,71);
R_Date("Bln-512",5775,100);
R_Date("Deb-2538",5765,40);
R_Date("Deb-2534",5760,40);
R_Date("Deb-3309",5752,58);
R_Date("Deb-10196",5750,50);
R_Date("Deb-3234",5745,63);
R_Date("Deb-2513",5740,40);
R_Date("Deb-2521",5740,40);
R_Date("Deb-2552",5735,40);
R_Date("Deb-3321",5703,69);
R_Date("Deb-3228",5701,62);
R_Date("Deb-1763",5700,40);
R_Date("Deb-1932",5700,40);
R_Date("Deb-1966",5700,40);
R_Date("Deb-3293",5699,66);
R_Date("Deb-3268",5698,71);
```

```
   R_Date("Deb-3316",5684,70);
   R_Date("Deb-1756",5680,40);
   R_Date("Deb-3269",5654,65);
   R_Date("Deb-3205",5636,55);
   R_Date("Deb-1758",5610,40);
  };
  Boundary("End Polgár-Csőszhalom");
  Span();
 };
};


{
 Sequence("Rogoza near Maribor")
 {
  Boundary("Start Rogoza near Maribor");
  Phase("Phase Rogoza near Maribor")
  {
   R_Date("KIA-37296",2977,24);
   R_Date("KIA-37293",2952,22);
   R_Date("KIA-37307",2932,27);
   R_Date("KIA-37294",2916,29);
   R_Date("KIA-37298",2910,31);
   R_Date("KIA-37295",2906,24);
   R_Date("KIA-37237",2900,27);
   R_Date("KIA-37291",2896,31);
   R_Date("KIA-37288",2890,25);
   R_Date("KIA-37305",2876,24);
   R_Date("KIA-37290",2864,27);
  };
  Boundary("End Rogoza near Maribor");
  Span();
 };
};


{
 Sequence("Sântana-Cetatea Veche")
 {
  Boundary("Start Sântana-Cetatea Veche");
  Phase("Phase Sântana-Cetatea Veche")
  {
   R_Date("MAMS 37708",3227,20);
   R_Date("MAMS 37710",3147,20);
   R_Date("MAMS 33948",3131,24);
```

```
  R_Date("MAMS 33945",3118,23);
  R_Date("MAMS 37707",3104,19);
  R_Date("MAMS 37714",3102,24);
  R_Date("MAMS 37706",3087,25);
  R_Date("MAMS 37713",3087,20);
  R_Date("MAMS 37712",3081,19);
  R_Date("MAMS 33946",3066,24);
  R_Date("MAMS 33944",3064,27);
  R_Date("MAMS 37711",3055,20);
  R_Date("MAMS 37709",3047,19);
 };
 Boundary("End Sântana-Cetatea Veche");
 Span();
};
};

{
 Sequence("Szederkény")
 {
  Boundary("Start Szederkény");
  R_Date("SUERC- 48423",6366,34);
  R_Date("MAMS- 14811",6362,33);
  R_Date("SUERC- 48425",6354,34);
  R_Date("OxA- 30514",6350,32);
  R_Date("SUERC- 54938",6342,37);
  R_Date("OxA-30515",6339,34);
  R_Date("OxA-30517",6332,33);
  R_Date("OxA-29053",6329,31);
  R_Date("SUERC- 48417",6326,34);
  R_Date("SUERC- 54937",6322,37);
  R_Date("OxA- 29051",6320,30);
  R_Date("OxA-29054",6314,33);
  R_Date("SUERC- 54928",6313,37);
  R_Date("OxA-28931",6309,32);
  R_Date("SUERC- 48424",6308,34);
  R_Date("SUERC- 54935",6299,37);
  R_Date("OxA- 28932",6297,31);
  R_Date("SUERC- 54933",6295,37);
  R_Date("OxA-30522",6295,33);
  R_Date("SUERC- 54934",6279,37);
  R_Date("SUERC- 54939",6278,37);
  R_Date("OxA- 29052",6273,31);
  R_Date("SUERC- 54936",6272,37);
  R_Date("MAMS-14809",6267,33);
  R_Date("OxA-30521",6266,32);
  R_Date("OxA-29050",6264,34);
```

```
  R_Date("OxA-28930",6260,32);
  R_Date("SUERC- 54929",6259,37);
  R_Date("OxA-30513",6250,32);
  R_Date("SUERC- 48419",6247,34);
  R_Date("OxA- 30518",6239,34);
  R_Date("OxA-30519",6226,33);
  R_Date("MAMS- 14810",6224,29);
  R_Date("SUERC- 54943",6224,37);
  R_Date("MAMS- 14812",6220,29);
  R_Date("OxA- 30520",6168,33);
  R_Date("OxA- 30516",6168,33);
  R_Date("OxA- 28933",6118,31);
  R_Date("SUERC- 48418",6078,34);
  Boundary("End Szederkény");
  Span();
 };
};

{
 Sequence("Szeghalom-Kovácshalom")
 {
  Boundary("Start Szeghalom-Kovácshalom");
  Phase("Phase Szeghalom-Kovácshalom")
  {
   R_Date("UGAMS-13255",6120,30);
   R_Date("UGAMS-13254",6100,25);
   R_Date("UGAMS-13253",6100,30);
   R_Date("UGAMS-13249",6070,30);
   R_Date("UGAMS-13251",6060,25);
   R_Date("UGAMS-13248",6040,30);
   R_Date("UGAMS-13252",6040,25);
   R_Date("UGAMS-13258",6040,30);
   R_Date("UGAMS-13257",6030,30);
   R_Date("UGAMS-13247",6020,30);
   R_Date("UGAMS-13250",6000,30);
  };
  Boundary("End Szeghalom-Kovácshalom");
  Span();
 };
};



{
```

```
 Sequence("Szemely-Irtás")
 {
  Boundary("Start Szemely-Irtás");
  Phase("Phase Szemely-Irtás")
  {
   R_Date("Beta-310041",6140,30);
   R_Date("Beta-310038",6110,30);
   R_Date("Beta-310034",6020,40);
   R_Date("Beta-310037",5990,40);
   R_Date("Beta-310040",5930,40);
   R_Date("Beta-310039",5920,40);
  };
  Boundary("End Szemely-Irtás");
  Span();
 };
};



{
 Sequence("Tiszalúc-Sarkad")
 {
  Boundary("Start Tiszalúc-Sarkad");
  Phase("Phase Tiszalúc-Sarkad")
  {
   R_Date("GrN-16129",5100,40);
   R_Date("GrN-16130",5085,40);
   R_Date("Poz-36361",5070,40);
   R_Date("Poz-36363",5050,40);
   R_Date("Poz-36362",5020,40);
   R_Date("GrN-16128",5020,40);
   R_Date("GrN-16127",4920,60);
  };
  Boundary("End Tiszalúc-Sarkad");
  Span();
 };
};


{
 Sequence("Tolna-Mözs")
 {
  Boundary("Start Tolna-Mözs");
  Phase("Phase Tolna-Mözs")
  {
   R_Date("Poz-149786",6350,40);
```

```
   R_Date("Poz-149784",6320,40);
   R_Date("SUERC-100838",6319,20);
   R_Date("SUERC-100839",6308,24);
   R_Date("Poz-149757",6300,50);
   R_Date("SUERC-100840",6283,24);
   R_Date("SUERC-100836",6272,23);
   R_Date("MAMS-14145",6233,23);
   R_Date("Poz-149758",6230,40);
   R_Date("Poz-149785",6220,40);
   R_Date("SUERC-100835",6178,20);
   R_Date("MAMS-14144",6143,24);
   R_Date("Poz-149756",6130,40);
  };
  Boundary("End Tolna-Mözs");
  Span();
 };
};
```

```
{
 Sequence("Túrkeve-Terehalom")
 {
  Boundary("Start Túrkeve-Terehalom");
  Phase("Phase Túrkeve-Terehalom")
  {
   R_Date("AAR-31587",3649,28);
   R_Date("AAR-31577",3602,27);
   R_Date("AAR-31585",3595,27);
   R_Date("AAR-31581",3592,30);
   R_Date("AAR-31589",3583,33);
   R_Date("AAR-31575",3573,29);
   R_Date("AAR-31576",3568,29);
   R_Date("AAR-31590",3565,27);
   R_Date("AAR-31574",3549,27);
   R_Date("AAR-31588",3549,26);
   R_Date("AAR-31586",3548,28);
   R_Date("AAR-31573",3542,26);
   R_Date("AAR-31564",3539,29);
   R_Date("AAR-31567",3539,29);
   R_Date("AAR-31582",3535,28);
   R_Date("AAR-31571",3529,30);
   R_Date("AAR-31566",3518,26);
   R_Date("AAR-31584",3515,26);
   R_Date("AAR-31572",3509,29);
   R_Date("AAR-31569",3498,27);
```

```
  R_Date("AAR-31580",3497,26);
  R_Date("AAR-31565",3488,28);
  R_Date("AAR-31578",3485,27);
  R_Date("AAR-31579",3467,35);
  R_Date("AAR-31559",3459,31);
  R_Date("AAR-31561",3450,27);
  R_Date("AAR-31558",3449,28);
  R_Date("AAR-31563",3446,30);
  R_Date("AAR-31570",3441,26);
  R_Date("AAR-31562",3431,30);
  R_Date("AAR-31560",3378,27);
  R_Date("AAR-31557",3321,29);
 };
 Boundary("End Túrkeve-Terehalom");
 Span();
};
};
```

```
{
Sequence("Uivar")
{
 Boundary("Start Uivar");
 Phase("Phase Uivar")
 {
  R_Date("UBA-22489",6275,40);
  R_Date("SUERC-50920",6262,30);
  R_Date("SUERC-50944",6253,30);
  R_Date("SUERC-48343",6238,34);
  R_Date("SUERC-48192",6235,33);
  R_Date("UBA-22520",6230,59);
  R_Date("SUERC-48198",6228,32);
  R_Date("Hd-24592",6224,51);
  R_Date("SUERC-50916",6221,27);
  R_Date("Poz-28048",6220,40);
  R_Date("Poz-28042",6210,40);
  R_Date("Poz-28044",6210,40);
  R_Date("UBA-22474",6209,42);
  R_Date("Poz-28050",6205,35);
  R_Date("SUERC-50926",6203,31);
  R_Date("SUERC-48347",6197,34);
  R_Date("SUERC-50917",6196,33);
  R_Date("SUERC-48181",6195,32);
  R_Date("SUERC-48345",6193,34);
```

```
R_Date("Poz-28045",6190,40);
R_Date("UBA-22518",6190,38);
R_Date("Poz-28047",6180,40);
R_Date("SUERC-48190",6174,33);
R_Date("SUERC-50919",6173,28);
R_Date("SUERC-48183",6172,33);
R_Date("SUERC-50927",6171,30);
R_Date("UBA-22469",6166,31);
R_Date("SUERC-50925",6163,33);
R_Date("Poz-28046",6160,30);
R_Date("SUERC-48180",6158,29);
R_Date("Hd-27754",6156,24);
R_Date("UBA-22519",6154,34);
R_Date("UBA-22495",6154,43);
R_Date("Hd-24593",6153,34);
R_Date("SUERC-50918",6148,33);
R_Date("SUERC-48188",6147,33);
R_Date("UBA-22471",6143,38);
R_Date("SUERC-50924",6140,30);
R_Date("SUERC-50940",6136,30);
R_Date("UBA-22466",6135,31);
R_Date("SUERC-48191",6132,33);
R_Date("Hd-24594",6131,35);
R_Date("KN-5951",6130,36);
R_Date("Hd-24606",6128,87);
R_Date("UBA-22479",6124,40);
R_Date("Hd-27755",6115,24);
R_Date("UBA-22475",6112,36);
R_Date("Hd-22980",6112,57);
R_Date("UBA-22468",6110,35);
R_Date("SUERC-48193",6109,30);
R_Date("UBA-22476",6104,33);
R_Date("UBA-22490",6103,47);
R_Date("UBA-22503",6103,37);
R_Date("Poz-28051",6100,35);
R_Date("SUERC-48200",6097,32);
R_Date("SUERC-48344",6097,34);
R_Date("SUERC-48220",6096,32);
R_Date("SUERC-48209",6093,32);
R_Date("SUERC-48197",6091,33);
R_Date("UBA-22472",6091,37);
R_Date("Hd-24549",6070,39);
R_Date("SUERC-48189",6069,30);
R_Date("UBA-22467",6068,34);
R_Date("SUERC-48346",6065,34);
R_Date("UBA-22494",6063,39);
```

```
R_Date("SUERC-50928",6061,31);
R_Date("Hd-24516",6061,35);
R_Date("SUERC-48210",6060,32);
R_Date("Hd-24531",6059,29);
R_Date("SUERC-48203",6058,32);
R_Date("Hd-24591",6056,43);
R_Date("UBA-22506",6056,35);
R_Date("UBA-22504",6054,32);
R_Date("SUERC-48212",6052,30);
R_Date("Poz-18975",6050,40);
R_Date("SUERC-48187",6047,33);
R_Date("SUERC- 48208",6043,32);
R_Date("Poz-18969",6040,40);
R_Date("Poz-18971",6040,30);
R_Date("Hd-22737",6036,22);
R_Date("UBA-22496",6036,38);
R_Date("SUERC-48348",6036,34);
R_Date("SUERC-48207",6033,36);
R_Date("SUERC-50935",6026,29);
R_Date("UBA-22473",6025,32);
R_Date("SUERC-48199",6025,32);
R_Date("Hd-22735",6022,28);
R_Date("UBA-22482",6022,43);
R_Date("SUERC-50929",6021,33);
R_Date("SUERC-50937",6014,32);
R_Date("UBA-22511",6011,33);
R_Date("SUERC-48219",6010,27);
R_Date("Hd-22756",6008,27);
R_Date("SUERC-48211",6007,37);
R_Date("SUERC-48202",6003,33);
R_Date("SUERC-50939",6003,33);
R_Date("SUERC-48213",6002,32);
R_Date("UBA-22500",5998,37);
R_Date("Hd-22734",5996,60);
R_Date("UBA-22501",5994,36);
R_Date("UBA-22488",5990,31);
R_Date("UBA-22481",5989,36);
R_Date("Hd-22754",5989,26);
R_Date("Hd-22973",5988,17);
R_Date("SUERC-50930",5985,31);
R_Date("UBA-22480",5984,30);
R_Date("UBA-22498",5984,35);
R_Date("UBA-22483",5981,39);
R_Date("UBA-22484",5962,38);
R_Date("Hd-24617",5959,28);
R_Date("UBA-22505",5956,39);
```

```
R_Date("SUERC-50934",5954,31);
R_Date("Hd-24529",5953,30);
R_Date("Hd-24497",5951,32);
R_Date("Hd-22736",5949,38);
R_Date("UBA-22491",5948,39);
R_Date("Hd-22688",5947,41);
R_Date("Hd-24530",5943,29);
R_Date("UBA-22521",5942,34);
R_Date("UBA-22507",5942,35);
R_Date("SUERC-48217",5941,40);
R_Date("UBA-22522",5941,48);
R_Date("UBA-22478",5932,37);
R_Date("Hd-24545",5928,28);
R_Date("UBA-22510",5927,36);
R_Date("Hd-24509",5919,36);
R_Date("UBA-22493",5907,37);
R_Date("SUERC-48349",5904,34);
R_Date("SUERC-50938",5903,33);
R_Date("Hd-27349",5902,36);
R_Date("Erl-2043",5899,84);
R_Date("Hd-22751",5896,36);
R_Date("UBA-22499",5889,38);
R_Date("UBA-22502",5886,40);
R_Date("Hd-22965",5885,39);
R_Date("SUERC-48218",5882,32);
R_Date("SUERC-50936",5876,32);
R_Date("Poz-28040",5875,35);
R_Date("Hd-24554",5873,36);
R_Date("UBA-22487",5871,39);
R_Date("Poz-28041",5870,40);
R_Date("Hd-24595",5868,42);
R_Date("Hd-24498",5867,33);
R_Date("Hd-24517",5867,29);
R_Date("Hd-22659",5862,32);
R_Date("UBA-22509",5862,36);
R_Date("Hd-27795",5859,24);
R_Date("Hd-24508",5848,36);
R_Date("Hd-24550",5847,26);
R_Date("SUERC-48353",5847,34);
R_Date("Hd-27786",5847,25);
R_Date("Hd-22971",5846,28);
R_Date("Hd-24496",5839,29);
R_Date("SUERC-48201",5833,30);
R_Date("Hd-24528",5831,24);
R_Date("Hd-27794",5823,33);
R_Date("Erl-2044",5804,85);
```

```
  R_Date("Hd-22658",5782,27);
  R_Date("KN5950",5781,50);
  R_Date("UBA-22508",5760,37);
  R_Date("Poz-18976",5760,40);
  R_Date("Hd-22928",5740,55);
  R_Date("Hd-27742",5733,30);
  R_Date("Poz-18967",5730,40);
  R_Date("Poz-18970",5730,40);
  R_Date("Hd-22930",5726,77);
  R_Date("SUERC-48354",5562,34);
  R_Date("Hd-27691",5522,26);
  R_Date("Poz-18973",5470,40);
  R_Date("Poz-18972",5440,40);
  R_Date("UBA-22492",5426,40);
  R_Date("Poz-19390",5410,40);
 };
 Boundary("End Uivar");
 Span();
};
};
```

```
{
 Sequence("Úľany nad Žitavou 'Dolné diely'")
 {
  Boundary("Start Úľany nad Žitavou 'Dolné diely'");
  Phase("Phase Úľany nad Žitavou 'Dolné diely'")
  {
   R_Date("Poz-109590",6280,40);
   R_Date("Poz-109589",6270,40);
   R_Date("Poz-109489",6270,40);
   R_Date("Poz-109593",6230,35);
   R_Date("Poz-109591",6190,40);
  };
  Boundary("End Úľany nad Žitavou 'Dolné diely'");
  Span();
 };
};
```

```
{
 Sequence("Versend-Gilencsa")
```

```
{
Boundary("Start Versend-Gilencsa");
Phase("Phase Versend-Gilencsa")
{
 R_Date("UBA-22609",6348,45);
 R_Date("MAMS-14830",6321,28);
 R_Date("SUERC-58558",6306,32);
 R_Date("SUERC-58579",6305,31);
 R_Date("SUERC-58570",6299,32);
 R_Date("SUERC-67309",6280,29);
 R_Date("UBA-22601",6276,42);
 R_Date("UBA-22606",6272,44);
 R_Date("SUERC-58564",6270,32);
 R_Date("SUERC-58556",6267,34);
 R_Date("SUERC-58550",6266,31);
 R_Date("SUERC-58575",6264,33);
 R_Date("SUERC-67280",6260,29);
 R_Date("SUERC-67296",6258,32);
 R_Date("SUERC-58560",6257,33);
 R_Date("UBA-22613",6257,41);
 R_Date("SUERC-67295",6257,32);
 R_Date("UBA-22605",6253,58);
 R_Date("UBA-22607",6251,43);
 R_Date("SUERC-58566",6250,33);
 R_Date("SUERC-58569",6247,33);
 R_Date("SUERC-67279",6247,29);
 R_Date("SUERC-67300",6238,29);
 R_Date("SUERC-58568",6235,31);
 R_Date("SUERC-67287",6233,33);
 R_Date("SUERC-58559",6229,31);
 R_Date("SUERC-58554",6229,34);
 R_Date("SUERC-67288",6227,30);
 R_Date("SUERC-58577",6226,32);
 R_Date("MAMS-14832",6226,30);
 R_Date("UBA-22600",6221,40);
 R_Date("SUERC-67289",6220,30);
 R_Date("SUERC-58567",6211,32);
 R_Date("MAMS-14831",6202,31);
 R_Date("UBA-22611",6201,49);
 R_Date("SUERC-58555",6199,32);
 R_Date("UBA-22603",6198,41);
 R_Date("SUERC-58574",6198,32);
 R_Date("SUERC-67290",6198,29);
 R_Date("UBA-22617",6198,39);
 R_Date("MAMS-14833",6186,29);
 R_Date("SUERC-58557",6185,34);
```

```
  R_Date("SUERC-58576",6180,32);
  R_Date("UBA-22597",6180,51);
  R_Date("UBA-22616",6172,38);
  R_Date("UBA-22599",6172,40);
  R_Date("SUERC-67285",6171,30);
  R_Date("SUERC-58565",6168,32);
  R_Date("SUERC-67298",6167,29);
  R_Date("UBA-22598",6166,50);
  R_Date("SUERC-67308",6166,29);
  R_Date("UBA-22612",6165,40);
  R_Date("SUERC-67286",6163,30);
  R_Date("SUERC-67281",6162,29);
  R_Date("SUERC-67301",6155,32);
  R_Date("SUERC-67299",6152,32);
  R_Date("UBA-22610",6141,43);
  R_Date("SUERC-67310",6140,29);
  R_Date("SUERC-67307",6125,29);
  R_Date("SUERC-67305",6059,29);
  R_Date("SUERC-67306",6047,29);
  };
 Boundary("End Versend-Gilencsa");
 Span();
 };
};
```

```
{
 Sequence("Vésztő, Körösladány-Bikeri")
 {
  Boundary("Start Vésztő, Körösladány-Bikeri");
  Phase("Phase Vésztő, Körösladány-Bikeri")
  {
   R_Date("Beta-162069",5790,100);
   R_Date("Beta-214597",5740,40);
   R_Date("Beta-234310",5730,40);
   R_Date("Beta-162065",5700,40);
   R_Date("Beta-162066",5660,40);
   R_Date("Beta-179791",5620,40);
   R_Date("Beta-214589",5610,40);
   R_Date("Beta-179784",5580,50);
   R_Date("Beta-234308",5560,40);
   R_Date("Beta-179785",5560,50);
   R_Date("Beta-179790",5550,40);
   R_Date("Beta-179788",5540,40);
```

```
   R_Date("Beta-179786",5540,60);
   R_Date("Beta-234307",5520,40);
   R_Date("Beta-179783",5520,50);
   R_Date("Beta-162070",5490,50);
   R_Date("Beta-214593",5480,50);
   R_Date("Beta-162068",5480,40);
   R_Date("Beta-179789",5460,50);
   R_Date("Beta-179792",5440,50);
   R_Date("Beta-179787",5440,140);
   R_Date("Beta-234314",5430,50);
   R_Date("Beta-162071",5430,40);
   R_Date("Beta-214595",5420,40);
   R_Date("Beta-179793",5420,50);
   R_Date("Beta-234306",5410,80);
   R_Date("Beta-214592",5410,40);
   R_Date("Beta-234312",5380,40);
   R_Date("Beta-214596",5370,40);
   R_Date("Beta-234313",5370,60);
   R_Date("Beta-162067",5320,60);
   R_Date("Beta-179782",5310,50);
  };
  Boundary("End Vésztő, Körösladány-Bikeri");
  Span();
 };
};


{
 Sequence("Vésztő-Mágor")
 {
  Boundary("Start Vésztő-Mágor");
  Phase("Phase Vésztő-Mágor")
  {
   R_Date("Bln-1628",6250,60);
   R_Date("Bln-1625",6150,60);
   R_Date("Bln-1626",6000,60);
   R_Date("Bln-1342",5970,80);
   R_Date("UGAMS-71417",5950,30);
   R_Date("UGAMS-71405",5970,25);
};
  Boundary("End Vésztő-Mágor");
  Span();
 };
};

{
```

```
 Sequence("Vinkovci-Sopot")
 {
  Boundary("Start Vinkovci-Sopot");
  Phase("Phase Vinkovci-Sopot")
  {
   R_Date("Z-2826",6340,100);
   R_Date("Z-3868",6295,135);
   R_Date("Z-3139",6020,100);
   R_Date("Z-3140",6010,100);
   R_Date("OxA-23594",5988,34);
   R_Date("OxA-23595",5962,33);
   R_Date("Z-3141",5960,100);
   R_Date("Beta-251907",5940,40);
   R_Date("Z-3869",5900,75);
   R_Date("Beta-230029",5880,40);
   R_Date("OxA-23596",5861,33);
   R_Date("Beta-251912",5860,50);
   R_Date("Beta-251908",5840,50);
   R_Date("Z-3143",5840,100);
   R_Date("Z-3870",5840,80);
   R_Date("Beta-251913",5800,50);
   R_Date("Z-2753",5790,125);
   R_Date("Beta-230031",5780,40);
   R_Date("Beta-230033",5760,40);
   R_Date("Z-3867",5715,155);
   R_Date("Beta-230032",5680,40);
   R_Date("Z-2752",5675,120);
   R_Date("Z-3142",5550,130);
   R_Date("Z-3866",5415,195);
   R_Date("Z-2827",5380,100);
   R_Date("Z-2754",5360,130);
   R_Date("Z-2911",5330,90);
   R_Date("Beta-230030",5300,40);
   R_Date("Z-2909",5220,100);
  };
  Boundary("End Vinkovci-Sopot");
  Span();
 };
};

{
 Sequence("Vlkas 'Do hulského chotára'")
 {
```

```
  Boundary("Start Vlkas 'Do hulského chotára'");
  Phase("Phase Vlkas 'Do hulského chotára'")
  {
   R_Date("Poz-109595",6180,40);
   R_Date("Poz-109587",6170,40);
   R_Date("Poz-109410",6170,40);
   R_Date("Poz-109576",6160,35);
   R_Date("Poz-109573",6160,35);
   R_Date("Poz-109523",6110,35);
   R_Date("Poz-109575",6105,35);
   R_Date("Poz-109283",6090,40);
   R_Date("Poz-109594",6085,35);
   R_Date("Poz-109525",6075,35);
   R_Date("Poz-109526",6050,35);
   R_Date("Poz-109526",6050,35);
  };
  Boundary("End Vlkas 'Do hulského chotára'");
  Span();
 };
};


{
 Sequence("Vráble 'Véľke Lehemby'")
 {
  Boundary("Start Vráble 'Véľke Lehemby'");
  Phase("Phase Vráble 'Véľke Lehemby'")
  {
   R_Date("Poz-87436",6300,50);
   R_Date("Poz-87446",6270,40);
   R_Date("Poz-69565",6260,40);
   R_Date("Poz-98350",6240,40);
   R_Date("Poz-87448",6220,40);
   R_Date("Poz-60638",6220,35);
   R_Date("Poz-98369",6210,40);
   R_Date("Poz-87449",6200,40);
   R_Date("Poz-87453",6190,40);
   R_Date("Poz-87453",6190,40);
   R_Date("Poz-67229",6190,40);
   R_Date("Poz-69568",6190,40);
   R_Date("Poz-998446",6180,40);
   R_Date("Poz-98346",6180,40);
   R_Date("Poz-98347",6170,40);
   R_Date("Poz-98359",6170,40);
   R_Date("Poz-98344",6170,40);
   R_Date("Poz-87443",6170,35);
```

```
R_Date("KIA-52451",6166,24);
R_Date("KIA-52444",6155,20);
R_Date("Poz-98345",6150,40);
R_Date("Poz-98351",6150,40);
R_Date("Poz-98360",6150,40);
R_Date("Poz-60642",6145,30);
R_Date("Poz-98354",6140,40);
R_Date("Poz-98449",6140,40);
R_Date("Poz-87447",6140,35);
R_Date("Poz-87439",6140,40);
R_Date("Poz-69563",6140,40);
R_Date("Poz-98349",6130,40);
R_Date("Poz-87456",6130,40);
R_Date("Poz-87441",6130,40);
R_Date("Poz-87454",6130,40);
R_Date("Poz-69571",6130,40);
R_Date("KIA-52449",6119,25);
R_Date("KIA-52448",6119,25);
R_Date("Poz-60643",6115,35);
R_Date("Poz-87475",6115,35);
R_Date("Poz-87477",6110,40);
R_Date("Poz-87450",6110,40);
R_Date("Poz-87451",6110,40);
R_Date("Poz-69565",6110,40);
R_Date("Poz-98352",6100,40);
R_Date("Poz-98355",6100,40);
R_Date("Poz-98361",6100,40);
R_Date("Poz-98447",6100,40);
R_Date("Poz-87445",6100,35);
R_Date("Poz-98368",6100,40);
R_Date("Poz-69570",6100,35);
R_Date("Poz-998448",6090,40);
R_Date("Poz-98358",6090,40);
R_Date("KIA-52447",6081,25);
R_Date("Poz-98364",6080,40);
R_Date("Poz-98444",6080,40);
R_Date("Poz-60641",6080,30);
R_Date("Poz-87476",6080,40);
R_Date("Poz-87472",6080,35);
R_Date("Poz-87440",6080,40);
R_Date("Poz-69566",6080,40);
R_Date("Poz-69564",6080,40);
R_Date("KIA-52708",6075,35);
R_Date("Poz-98356",6070,40);
R_Date("Poz-87437",6070,40);
R_Date("KIA-52446",6060,24);
```

```
  R_Date("Poz-87474",6060,35);
  R_Date("Poz-98357",6050,40);
  R_Date("Poz-60611",6050,35);
  R_Date("KIA-52445",6044,25);
  R_Date("Poz-98362",6030,40);
  R_Date("Poz-60639",6015,35);
  R_Date("KIA-52450",6002,25);
  R_Date("Poz-98348",6000,40);
  R_Date("Poz-87438",6000,35);
  R_Date("Poz-60637",6000,50);
  R_Date("Poz-60609",5985,35);
  R_Date("Poz-87473",5960,40);
  R_Date("Poz-98342",5920,40);
  R_Date("Poz-60610",5920,35);
  R_Date("Poz-60640",5885,35);
  R_Date("Poz-998445",5870,40);
  R_Date("Poz-98367",5860,40);
  R_Date("Poz-98793",5840,35);
  R_Date("Poz-98336",5840,40);
 };
 Boundary("End Vráble 'Véľke Lehemby'");
 Span();
};
};
```

Results

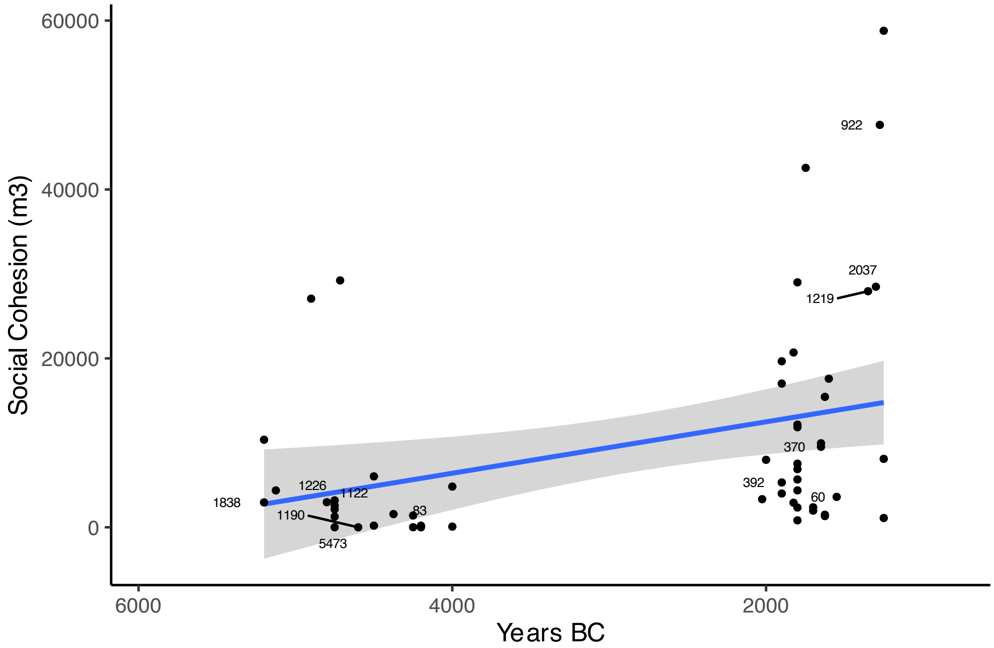

fig. S1. Social cohesion scores over time illustrating ditch lengths observed but not included in calculation. Cornești, an outlier at 223692 m^3, is not shown. Its unmeasured but identifiable ditches measure 20007 m in length.

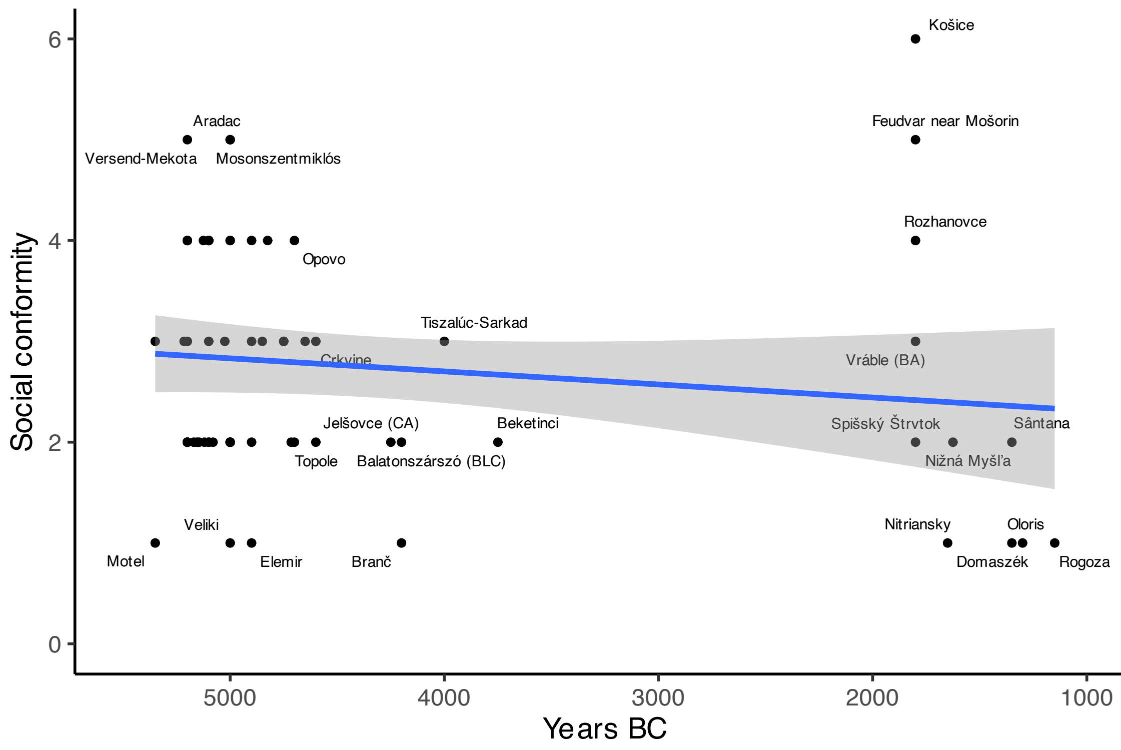

fig. S2. Social conformity scores over time.

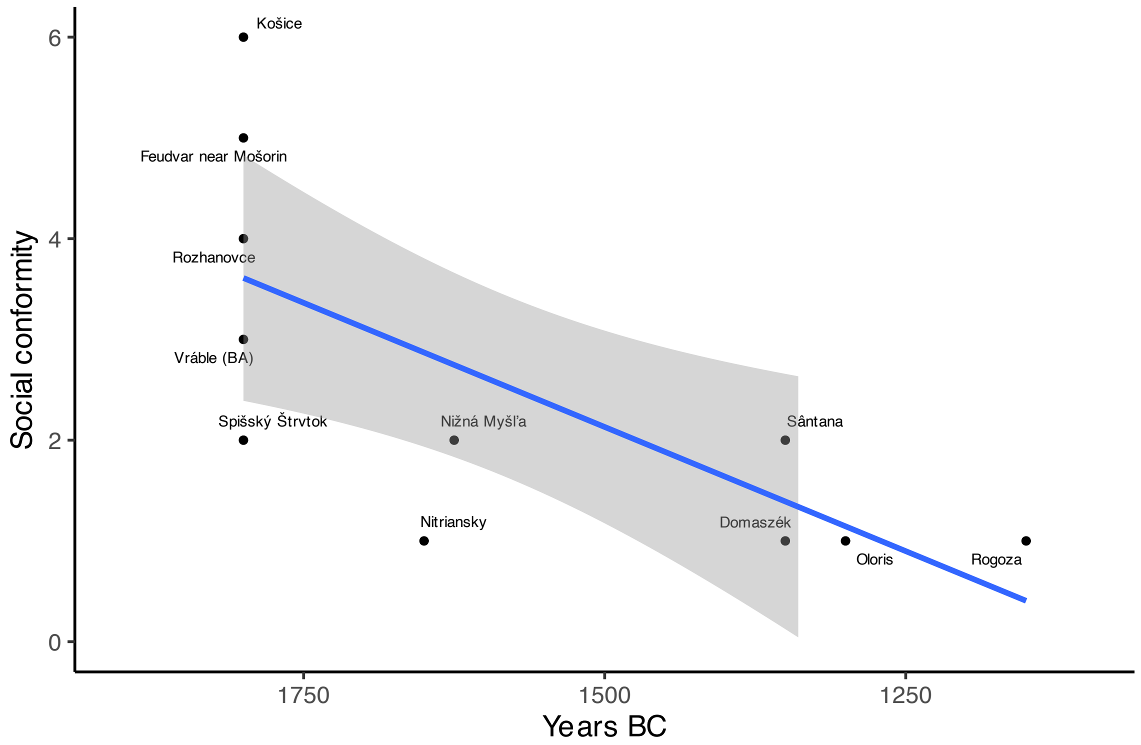

fig. S3. Social conformity over time in the Bronze Age.

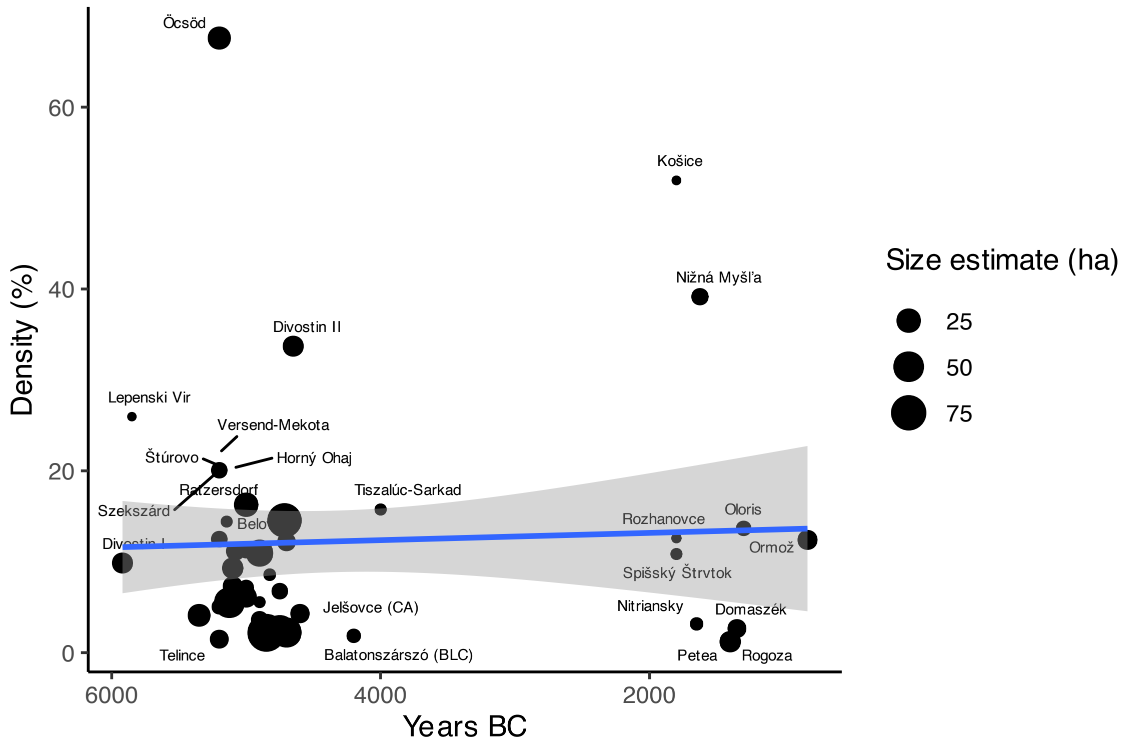

fig. S4. Site density over time, with proportional size.

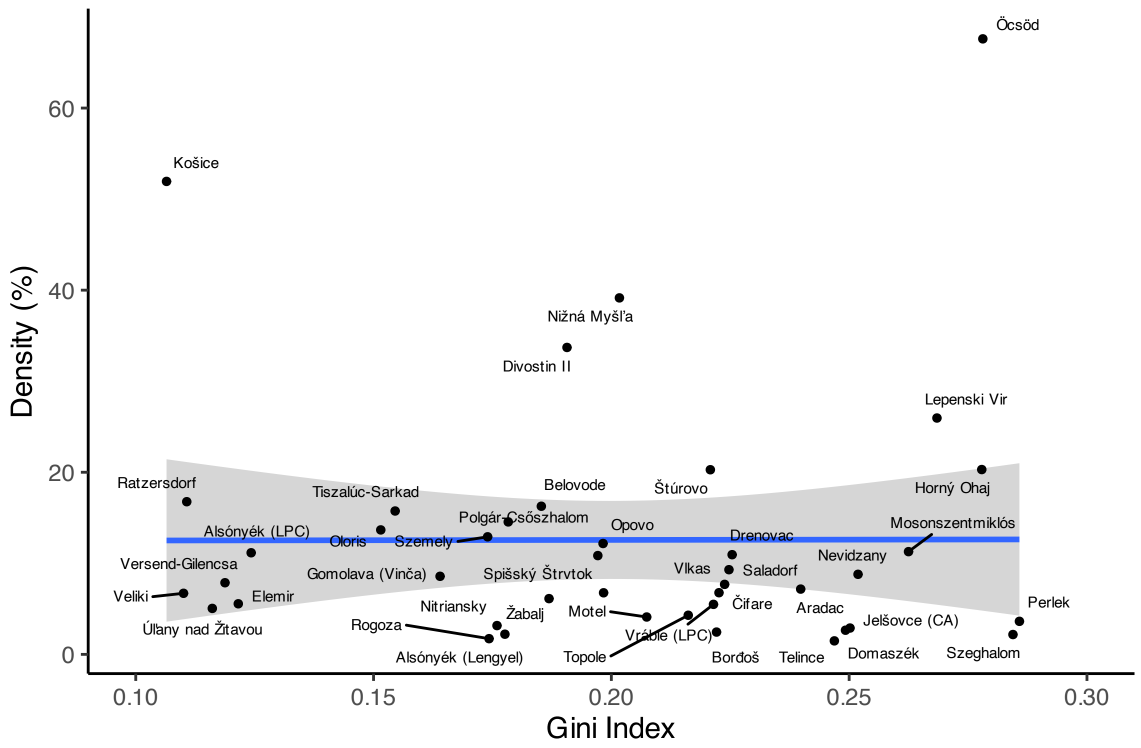

fig. S5. Scatterplot of social conformity scores against Gini index values.

table S1. Data for sites in the study sample.

ID	site_short	total_size_estimate	density_ha	social_conformity	period_fine_simple	vol_total	med	GINI
1	Aba	1.4			MBA	1970.5		
2	Alsónyék (Lengyel)	47.5	24.3	2	LN		880	0.178
3	Alsónyék (LPC)	10	4.5	2	MN			0.124
4	Aradac	4.3	2.3	5	LN			0.240
5	Balatonszárszó (BLC)	3	3	2	ECA	192		0.059
6	Balatonszárszó (LPC)	10	10	2	MN		140	0.222
7	Balatonszemes			2	MN			0.168
8	Banjica				LN			0.270
9	Beketinci		3.1	2	MCA			0.602
10	Belovode	25	16.1	4	LN		915	0.185
11	Bogács				MBA	12162.5		
12	Borđoš	50	31.1	3	LN		430	0.222
13	Borsodivánka	5.1			MBA	5664		
14	Branč	1.5	1.5	1	ECA			0.620
15	Brunn am Gebirge		16.74	3	EN		640	0.054
16	Carei Bobald	12.3			MBA	17017		
17	Čierne Kľačany	8.1	7.4	4	MN			0.216
18	Čifare	10.7	6.1	3	MN			0.223
19	Corneşti	1722			LBA	223692	290	
20	Crkvine	12.5	8	3	LN			0.162
21	Csanádpalota	460			LBA	47650.4	185	
22	Divostin I	15	0.24	1	EN		145	0.303

23	Divostin II	15	0.24	3	LN		290	0.191
24	Domaszék	10	0.98	1	LBA			0.249
25	Drenovac	35	10.6	4	LN		640	0.225
26	Elemir	0.95	0.7	1	LN			0.122
27	Feudvar near Mošorin	7.2		5	MBA	11850	255	
28	Füzesabony-G.	5.8	0.9	4	EN		335	0.082
29	Füzesabony-P.	0.5			MCA	9.1	15	
30	Gelej				MBA	7992		
31	Gomolava (Vinča)	1.38	0.5	4	LN		295	0.164
32	Gorjani	11	11	2	LN	1620		0.216
33	Gradište Iđoš	200			LBA	28483.9	190	
34	Hajdúböszörmény	8			LN	2971.5		
35	Hódmezővásárhely-Gorzsa	3.5			LN	2593.5	725	
36	Hódmezővásárhely-Kökénydomb	5.8	5.8	3	LN	2136.76	460	0.198
37	Horný Ohaj		0.5		MN			0.278
38	Jelšovce (CA)		3.7	2	ECA			0.250
39	Kajászó	1.8			MBA	17595		
40	Kakucs	2.2			MBA	2912		
41	Keszthely	12.4	1.8	2	MN			0.317
42	Košice	0.25	0.16	6	MBA	2340		0.106
43	Lepenski Vir	0.24	0.24	2	EN		545	0.268
44	Lovasberény	1.7			EBA	3330		
45	Makaranda	1.9	1.4	2	LN			0.180
46	Makó	36			LBA	1101.6		
47	Maňa		3.6	2	MN			0.232
48	Mezőcsát	8.92			MBA	6887.5		

49	Mold	4	1.18	2	MN		370	0.312
50	Mosonszentmiklós	6	1.8	5	MN			0.262
51	Motel	20	2.7	1	MN			0.207
52	Nagykőrös	2.83			MBA	2370		
53	Nevidzany		2.9	4	MN			0.252
54	Nitriansky Hrádok	2	0.8	1	EBA	9528.75		0.176
55	Nižná Myšl’a	7	0.12	2	MBA	15444		0.202
56	Nyíregyháza	13			LBA			0.242
57	Öcsöd	21	0.1	4	LN	2948.16	365	0.278
58	Oloris	4.2	0.169	1	LBA			0.152
59	Opovo	9.5	9.3	4	LN		60	0.198
60	Ormož	12.5	0.7	1	LBA			0.316
61	Orosháza	113			LBA	58792.5		
62	Otomani-Cetatea	5.2			MBA	9948		
63	Otomani-Cetățuie	2.77			MBA	4000.8		
64	Padina	2		3	EN			0.166
65	Parţa	9	0.15	4	MN		755	0.206
66	Pecica	27.2			MBA	42560	300	
67	Perlek	7	2.9	2	LN			0.286
68	Petea	16	0.93		LBA			0.331
69	Pir	2.4			MBA	3600		
70	Pişcolt	1.71			MBA	5310		
71	Polgár-Bosnyákdomb	6			LN	1267.2		
72	Polgár-Csőszhalom	70	3.04	2	LN	29232	355	0.178
73	Polgár-Nagy Kasziba				MN	191.75		

74	Ratzersdorf		1.03	3	MN			0.111
75	Rogoza		3	1	LBA		90	0.174
76	Rozhanovce	0.32	0.09	4	MBA	4371		0.073
77	Sălacea Dealul Vida	2			MBA	19660.2		
78	Saladorf		2.74	2	MN			0.224
79	Sântana	90		2	LBA	27954.5	150	
80	Soroksár	2.5			MBA	1328.65		
81	Spišský Štrvtok	1	0.66	2	MBA	822		0.197
82	Štúrovo		1.3	2	MN			0.221
83	Szakáld	6.86			MBA	10368		
84	Szarvas	1.8			MCA	4821		
85	Szederkény		6.16	2	EN		150	0.079
86	Szeghalom	90	50	3	LN		265	0.284
87	Szekszárd	5.7	1.8	3	EN			0.029
88	Szemely		4.8	3	MN		335	0.174
89	Taraš	2.2		2	LN			
90	Tard	5.37			MBA	7531.88		
91	Telince	10	8.2		MN			0.247
92	Tiszaalpár				MBA	1487.5		
93	Tiszalúc-Sarkad	1.07	1.07	3	MCA	79.976	95	0.155
94	Tiszaug-Kisrétpart	2			ECA	1398.6		
95	Tolna	30		2	LN		125	0.155
96	Túrkeve	3			MBA	20697.5	250	
97	Uivar	12			LN	27070.9	895	
98	Úľany nad Žitavou	4.2	4.2	2	MN		70	0.116

99	Végegyháza	7			LBA	8102.55		
100	Veliki	6	1	1	LN			0.110
101	Versend-Gilencsa		2.6	3	EN		205	0.119
102	Versend-Mekota		1.7	5	MN			0.016
103	Bikeri	1.5			ECA	1566.01	275	
104	Vésztő-Mágor	3.9			LN	3185	440	
105	Vinkovci	1.1			LN	6030	975	
106	Vlkas	15.8	6.6	4	MN		85	0.225
107	Vráble (LPC)	50	41	4	MN	4366.25	445	0.221
108	Vráble (BA)	14.2		3	MBA	29006.8		
109	Žabalj	15.6	12	4	LN			0.187
110	Zalavár	0.96	0.19	2	MCA			0.402

table S2. Ditch volume calculations for sites in the sample.

site_short	ID	vol_total	unmeasured
Aba	1	1970.5	
Alsónyék (Lengyel)	2		
Alsónyék (LPC)	3		
Aradac	4		
Balatonszárszó (BLC)	5	192	
Balatonszárszó (LPC)	6		
Balatonszemes	7		
Banjica	8		
Beketinci	9		
Belovode	10		
Bogács	11	12162.5	
Borđoš	12		
Borsodivánka	13	5664	
Branč	14		
Brunn am Gebirge	15		
Carei Bobald	16	17017	
Čierne Kľačany	17		
Čifare	18		
Corneşti	19	223692.17	20007.5
Crkvine	20		
Csanádpalota	21	47650.39	922
Divostin I	22		
Divostin II	23		
Domaszék	24		
Drenovac	25		
Elemir	26		
Feudvar near Mošorin	27	11850	
Füzesabony-G.	28		
Füzesabony-P.	29	9.1	83
Gelej	30	7992	
Gomolava (Vinča)	31		
Gorjani	32	1620	920
Gradište Iđoš	33	28483.884	2037
Hajdúböszörmény	34	2971.5	1226
Hódmezővásárhely-Gorzsa	35	2593.5	
Hódmezővásárhely-Kökénydomb	36	2136.755	1122
Horný Ohaj	37		
Jelšovce (CA)	38		

Kajászó	39	17595	
Kakucs	40	2912	
Keszthely	41		
Košice	42	2340	
Lepenski Vir	43		
Lovasberény	44	3330	
Makaranda	45		
Makó	46	1101.6	
Maňa	47		
Mezőcsát	48	6887.5	
Mold	49		
Mosonszentmiklós	50		
Motel	51		
Nagykőrös	52	2370	
Nevidzany	53		
Nitriansky Hrádok	54	9528.75	
Nižná Myšl'a	55	15444	
Nyíregyháza	56		
Öcsöd	57	2948.1563	1838
Oloris	58		
Opovo	59		
Ormož	60		
Orosháza	61	58792.5	
Otomani-Cetatea	62	9948	
Otomani-Cetățuie	63	4000.8	
Padina	64		
Parţa	65		
Pecica	66	42560	
Perlek	67		
Petea	68		
Pir	69	3600	60
Pişcolt	70	5310	392
Polgár-Bosnyákdomb	71	1267.2	
Polgár-Csőszhalom	72	29232	
Polgár-Nagy Kasziba	73	191.75	
Ratzersdorf	74		
Rogoza	75		
Rozhanovce	76	4371	
Sălacea Dealul Vida	77	19660.2	
Saladorf	78		
Sântana	79	27954.52	1219
Soroksár	80	1328.652	
Spišský Štrvtok	81	822	

Štúrovo	82		
Szakáld	83	10368	
Szarvas	84	4821	
Szederkény	85		
Szeghalom	86		
Szekszárd	87		
Szemely	88		
Taraš	89		
Tard	90	7531.875	370
Telince	91		
Tiszaalpár	92	1487.5	
Tiszalúc-Sarkad	93	79.976	
Tiszaug-Kisrétpart	94	1398.6	
Tolna	95		
Túrkeve	96	20697.5	
Uivar	97	27070.906	
Úľany nad Žitavou	98		
Végegyháza	99	8102.55	
Veliki	100		
Versend-Gilencsa	101		
Versend-Mekota	102		
Bikeri	103	1566.01	
Vésztő-Mágor	104	3185	
Vinkovci	105	6030	
Vlkas	106		
Vráble (LPC)	107	4366.25	
Vráble (BA)	108	29006.75	
Žabalj	109		
Zalavár	110		

table S3. Details of coding scheme for social conformity. An additional point was awarded when houses were similarly oriented.

Score	Pattern
1	one individual house or houses fully autonomous, i.e. spatially isolated, or total chaos
2	some houses forming a group relating to a linear or circular structure, or several different such groups
3	most houses following pattern (linear or circular), but there are different patterns
4	most houses somewhat following one pattern (linear or circular)
5	most houses strictly following a linear or circular pattern

Data S1. Site sample data. This including details of ditch features for social cohesion score, house sizes for Gini index, and calculated Span outputs representing Site Apogee. (separate file)

Data S2. Raw radiocarbon data used to calculate Site Apogee. (separate file)

REFERENCES AND NOTES

1. A. Bogaard, M. Fochesato, S. Bowles, The farming-inequality nexus: New insights from ancient Western Eurasia. *Antiquity* **93**, 1129–1143 (2019).

2. T. A. Kohler, M. E. Smith, A. Bogaard, G. M. Feinman, C. E. Peterson, A. Betzenhauser, M. Pailes, E. C. Stone, A. Marie Prentiss, T. J. Dennehy, L. J. Ellyson, L. M. Nicholas, R. K. Faulseit, A. Styring, J. Whitlam, M. Fochesato, T. A. Foor, S. Bowles, Greater post-Neolithic wealth disparities in Eurasia than in North America and Mesoamerica. *Nature* **551**, 619–622 (2017).

3. S. Bowles, M. Fochesato, The origins of enduring economic inequality. *J. Natl. Med. Assoc.* **62**, 1475–1537 (2024).

4. L. A. White, *The Science of Culture, a Study of Man and Civilization* (Farrar, 1949).

5. R. C. Allen, The Neolithic Revolution in the Middle East. *Econ. Hist. Rev.* **77**, 1154–1196 (2024).

6. W. Scheidel, *The Great Leveler: Violence and the History of Inequality from the Stone Age to the Twenty-First Century* (Princeton University Press, 2017).

7. F. Schlütz, R. Hofmann, M. Dal Corso, G. Pashkevych, S. Dreibrodt, M. Shatilo, A. Ţerna, K. Fuchs, D. Filipović, P. Flohr, C. A. Makarewicz, S. Ţerna, M. Videiko, V. Rud, J. Müller, W. Kirleis, Isotopes prove advanced, integral crop production, and stockbreeding strategies nourished Trypillia mega-populations. *Proc. Natl. Acad. Sci. U.S.A.* **120**, e2312962120 (2023).

8. R. Hofmann, N. Müller-Scheeßel, J. Müller, Trypillia mega-sites: A social levelling concept? *Antiquity* **98**, 380–400 (2024).

9. J. Laabs, Wealth consumption, sociopolitical organization, and change: A perspective from burial analysis on the Middle Bronze Age in the Carpathian Basin. *Open Archaeol.* **9**, 20220281 (2023).

10. F. Wilkes, “Die Entwicklung sozialer Ungleichheit im Karpatenbecken zwischen 5200 und 1000 v.u.Z. Eine quantitative Studie an neolithischen, kupferzeitlichen und bronzezeitlichen Gräberfeldern und methodische Überlegungen,” thesis, Christian-Albrechts-Universität zu Kiel (2024).

11. M. Borgerhoff Mulder, S. Bowles, T. Hertz, A. Bell, J. Beise, G. Clark, I. Fazzio, M. Gurven, K. Hill, P. L. Hooper, W. Irons, H. Kaplan, D. Leonetti, B. Low, F. Marlowe, R. McElreath, S. Naidu, D. Nolin, P. Piraino, R. Quinlan, E. Schniter, R. Sear, M. Shenk, E. A. Smith, C. von Rueden, P. Wiessner, Intergenerational wealth transmission and the dynamics of inequality in small-scale societies. *Science* **326**, 682–688 (2009).

12. D. M. Carballo, *Cooperation and Collective Action: Archaeological perspectives* (Univ. Press of Colorado, 2013).

13. J. Lund, M. Furholt, K. I. Austvoll, Reassessing power in the archaeological discourse. How collective, cooperative and affective perspectives may impact our understanding of social relations and organization in prehistory. *Archaeol. Dialogues.* **29**, 33–50 (2022).

14. G. M. Feinman, D. M. Carballo, Collaborative and competitive strategies in the variability and resiliency of large-scale societies in Mesoamerica. *Econ. Anthropol.* **5**, 7–19 (2018).

15. R. E. Blanton, L. Fargher, *Collective Action in the Formation of Pre-Modern States*, Fundamental Issues in Archaeology (Springer, 2008).

16. G. M. Feinman, J. E. Neitzel, The social dynamics of settling down. *J. Anthrop. Archaeol.* **69**, 101468 (2023).

17. J. Chapman, S. Souvatzi, The Neolithic of Southeast Europe: Recent trends. *Annu. Rev. Anthrop.* **49**, 123–140 (2020).

18. A. Anders, Z. Siklósi, *The Körös Culture in Eastern Hungary*, The First Neolithic Sites in Central/South-East European Transect (Archaeopress, 2012).

19. L. Bartosiewicz, “New data on the prehistoric fauna of the Iron Gates: A case study from Schela Cladovei, Romania,” in *From the Mesolithic to the Neolithic (Proceedings of the*

International Archaeological Conference Held in the Damjanich Museum of Szolnok, September 22–27, 1996), C. Bonsall, V. Boroneanţ, Eds. (Archaeolingua, 2001), pp. 15–21.

20. P. Sümegi, R. Kertész, G. Timár, K. Herbich, "Palaeoenvironmental factors and Neolithization process of the Carpathian Basin: Some aspects of a new geoarchaeological model," in *Acts of the XIVth Union International of Prehistoric and Protohistoric Sciences, Liège, Belgium, 2–8 September 2001, Section 1: Theory and Methods,* P. Cattelain, Ed. (British Archaeolgoical Reports, 2003), vol. 1145, pp. 135–141.

21. F. Draşovean, W. Schier, "The Neolithic tell sites of Parţa and Uivar (Romanian Banat). A comparison of their architectural sequence and organization of social space," in *Leben auf dem Tell als soziale Praxis: Beiträge des Internationalen Symposiums in Berlin vom 26.–27. Februar 2007*, S. Hansen, Ed. (Dr. Rudolf Habelt GmbH, 2010), pp. 165–187.

22. M. Marić, B. Roberts, M. Radivojević, "Chapter 10: Belovode: Excavation results," in *The Rise of Metallurgy in Eurasia: Evolution, Organisation And Consumption of Early Metal in the Balkans,* M. Radivojević, B. Roberts, M. Marić, J. Kuzmanović Cvetković, T. Rehren, Eds. (Archaeopress, 2021), pp. 108–122.

23. M. Furholt, I. Cheben, J. Müller, A. Bistáková, M. Wunderlich, N. Müller-Scheeßel, *Archaeology in the Žitava Valley I: The LBK and Želiezovce Settlement Site of Vráble* (Sidestone Press, 2020).

24. M. Furholt, N. Müller-Scheeßel, M. Wunderlich, I. Cheben, J. Müller, Communality and discord in an Early Neolithic settlement agglomeration: The LBK site of Vráble, Southwest Slovakia. *Camb. Archaeol. J.* **30**, 469–489 (2020).

25. P. Raczky, A. Anders, Neolithic enclosures in Eastern Hungary and their survival into the Copper Age. *Tagungen des Landesmuseums für Vorgeschichte* **8**, 271–309 (2012).

26. R. Hofmann, A. Medović, M. Furholt, I. Medović, T. S. Pešterac, S. Dreibrodt, S. Martini, A. Hofmann, Late Neolithic multicomponent sites of the Tisza region and the emergence of centripetal settlement layouts. *Praehist Z.* **94**, 351–378 (2019).

27. D. Riebe, J. Dani, A. Gyucha, M. Stibrányi, G. Mesterházy, M. Pethe, A. Sarris, A. V. Argyriou, V. Nuccio, Tell a different story: Preliminary results from investigations at the Late Neolithic Herpály settlement complex of Csökmő-Káposztás-domb. *Hung. Archaeol.* **12**, 13 (2023).

28. A. Gyucha, W. A. Parkinson, R. W. Yerkes, The evolution of a Neolithic tell on the Great Hungarian Plain: Site formation and use at Szeghalom-Kovácshalom. *J. Field Archaeol.* **44**, 458–479 (2019).

29. W. A. Parkinson, A. Gyucha, P. Karkanas, N. Papadopoulos, G. Tsartsidou, A. Sarris, P. R. Duffy, R. W. Yerkes, A landscape of tells: Geophysics and microstratigraphy at two Neolithic tell sites on the Great Hungarian Plain. *J. Archaeol. Sci. Rep.* **19**, 903–924 (2018).

30. J. Řídký, P. Květina, H. Stäuble, I. Pavlů, What is changing and when – Post linear pottery culture life in central Europe. *Anthropologie* **52**, 333–340 (2015).

31. W. A. Parkinson, Tribal boundaries: Stylistic variability and social boundary maintenance during the transition to the Copper Age on the Great Hungarian Plain. *J. Anthrop. Archaeol.* **25**, 33–58 (2006).

32. A. Gyucha, W. A. Parkinson, R. W. Yerkes, "Late Neolithic to the Early Copper Age: Multidisciplinary investigations in the Körös region of the Great Hungarian Plain," in *The Neolithic and Eneolithic in Southeast Europe: New Approaches to Dating and Cultural Dynamics in the 6th to 4th Millennium BC*, W. Schier, F. Draşovean, Eds. (Leidorf, 2014), pp. 273–296.

33. W. P. Ridge, "Sociocultural and demographic change in villages on the Great Hungarian Plain c. 4500-3600 BCE," thesis, University of Illinois at Chicago, Chicago (2023).

34. J. Dani, Preda-Bălănică, J. Angi, "The emergence of a new elite in southeastern Europe," in *First Kings of Europe: From Farmers to Rulers in Prehistoric Southeastern Europe*, A. Gyucha, W. A. Parkinson, Eds. (UCLA Cotsen Institute of Archaeology Press, 2022), pp. 60–77.

35. L. Bartosiewicz, “Nature or nurture? Climate, landscape and animal exploitation in Copper Age Hungary,” in *Walking Among Ancient Trees : Studies in Honour of Ryszard Grygiel and Peter Bogucki on the 45th Anniversary of their Research Collaboration*, G. Michal, J. O. Peter, Eds. (Fundacja Badań Archeologicznych Imienia Profesora Konrada Jażdżewskiego, 2023), pp. 305–316.

36. M. Jaeger, *Bronze Age Fortified Settlements in Central Europe* (Wydawnictwo Nauka i Innowacje, Dr Rudolf Habelt GmbH, 2016).

37. K. P. Fischl, V. Kiss, G. Kulcsár, V. Szeverényi, “Old and new narratives for Hungary around 2200 BC,” in *2200 BC - Ein Kilmasturz als Ursache für den Zerfall der Alten Welt? 2200 BC - A Climatic Breakdown as a Cause for the Collapse of the Old World?,* H. H. Meller, H. W. Arz, R. Jung, R. Risch, Eds. (Landesamt für Denkmalpflege und Archäeologie Sachsen-Anhalt, 2015), pp. 503–523.

38. J. Bátora, *Das Gräberfeld von Jelšovce / Slowakei*: *Prähistorische Archäologie in Südosteuropa: Ein Beitrag zur Frühbronzezeit im nordwestlichen Karpatenbecken* (Oetker/Voges, 2000), vol. 16.

39. P. R. Duffy, *Complexity and Autonomy in Bronze Age Europe: Assessing Cultural Developments in Eastern Hungary* (Archaeolingua, 2014).

40. T. L. Kienlin, K. P. Fischl, L. Marta, Exploring divergent trajectories in Bronze Age landscapes: Tell settlement in the Hungarian Borsod Plain and the Romanian Ier Valley. *Ziridava. Stud. Arch.* **31**, 93–128 (2017).

41. G. Fazecaş, F. Gogâltan, “The Otomani communities in the Crişuri Rivers Basin. A new perspective on the south-eastern border of the Otomani-Füzesabony Cultural Complex (OFCC),” in *Beyond Divides - The Otomani-Füzesabony Phenomenon*, K. P. Fischl, T. L. Kienlin, Eds. (Verlag Dr. Rudolf Habelt GmbH, 2019), pp. 317–350.

42. V. Kiss, *Middle Bronze Age Encrusted Pottery in Western Hungary* (Archaeolingua, 2012).

43. M. Jaeger, "Open communities – enclosed spaces. Kakucs-Turján settlement in the context of local tradition and interregional relations," in *Kakucs-Turján: A Middle Bronze Age Multi-layered Fortified Settlement in Central Hungary,* M. Jaeger, G. Kulcsár, N. Taylor, R. Staniuk, Eds. (Habelt, 2018), pp. 191–211.

44. R. Staniuk, Early and Middle Bronze Age chronology of the Carpathian Basin revisited: Questions answered or persistent challenges? *Radiocarbon* **63**, 1525–1546 (2021).

45. C. Cavazzuti, A. Arena, A. Cardarelli, M. Fritzl, M. Gavranović, T. Hajdú, V. Kiss, K. Köhler, G. Kulcsár, E. Melis, K. Rebay-Salisbury, G. Szabó, V. Szeverényi, the first 'urnfields' in the plains of the Danube and the Po. *J. World Prehist.* **35**, 45–86 (2022).

46. B. Molloy, D. Jovanović, C. Bruyere, M. Estanqueiro, M. Birclin, L. Milašinović, A. Šalamon, K. Penezić, C. B. Ramsey, D. Grosman, Resilience, innovation and collapse of settlement networks in later Bronze Age Europe: New survey data from the southern Carpathian Basin. *PLOS ONE* **18**, e0288750 (2023).

47. C. Metzner-Nebelsick, C. Kacsó, L. D. Nebelsick, A Bronze Age ritual structure on the edge of the Carpathian Basin. *Satu Mare - Studii şi Comunicări* **26**, 219–234 (2010).

48. B. S. Heeb, A. Szentmiklosi, A. Bălărie, R. Lehmpuhl, R. Krause, "Corneşti-Iarcuri – 10 years of research (2007–2016). Some important preliminary results," in *Fortifications: The Rise And Fall Of Defended Sites In Late Bronze And Early Iron Age Of South-East Europe,* B. S. Heeb, A. Szentmiklosi, R. Krause, M. Wemhoff, Eds. (Staatliche Museen zu Berlin, 2017), pp. 217–228.

49. S. van Willigen, S. Ozainne, M. Guélat, A.-L. G. Haller, M. Haller, New evidence for prehistoric ploughing in Europe. *Humanit. Soc. Sci. Commun.* **11**, 372 (2024), 10.1057/s41599-024-02837-5.

50. J. Dani, "Fortified tell settlements from the Middle Bronze Age in the Hungarian reach of the Berettyó Valley," in *Enclosed Space - Open Society. Contact and Exchange in the Context of Bronze Age Fortified Settlements in Central Europe*, M. Jaeger, J. Czebresuk, K. P.Fischl, Eds., SAO/SPEŚ 9 (Dr. Rudolf Habelt GmbH, 2012), pp. 27–37.

51. T. K. Earle, K. Kristiansen, *Organizing Bronze Age Societies: The Mediterranean, Central Europe, and Scandinavia Compared,* Dr. Rudolf Habelt GmbH (Cambridge Univ. Press, 2012).

52. M. Fochesato, A. Bogaard, S. Bowles, Comparing ancient inequalities: The challenges of comparability, bias and precision. *Antiquity* **93**, 853–869 (2019).

53. M. Fochesato, C. Higham, A. Bogaard, C. C. Castillo, Changing social inequality from first farmers to early states in Southeast Asia. *Proc. Natl. Acad. Sci. U.S.A.* **118**, e2113598118 (2021).

54. F. G. De Maio, Income inequality measures. *J. Epidemiol. Community Health* **61**, 849–852 (2007).

55. L. Osberg, On the limitations of some current usages of the Gini index. *Rev. Income Wealth.* **63**, 574–584 (2017).

56. G. Deltas, The small-sample bias of the Gini coefficient: Results and implications for empirical research. *Rev. Econ. Stat.* **85**, 226–234 (2003).

57. E. C. Stone, "The trajectory of social inequality in Ancient Mesopotamia," in *Ten Thousand Years of Inequality: The Archaeology of Wealth Differences,* T. A. Kohler, M. E. Smith, Eds., Amerind Studies in Anthropology (The University of Arizona Press, 2018), pp. 230–261.

58. P. Basri, D. Lawrence, Wealth inequality in the ancient Near East: A preliminary assessment using Gini coefficients and household size. *Camb. Archaeol. J.* **30**, 689–704 (2020).

59. J. Beck, C. P. Quinn, Balancing the scales: Archaeological approaches to social inequality. *Wld. Archaeol.* **54**, 572–583 (2022).

60. M. Gurven, M. B. Mulder, P. L. Hooper, H. Kaplan, R. Quinlan, R. Sear, E. Schniter, C. von Rueden, S. Bowles, T. Hertz, A. Bell, Domestication alone does not lead to inequality. *Curr. Anthrop.* **51**, 49–64 (2010).

61. G. M. Feinman, “The emergence of social complexity: Why more than population size matters,” in *Cooperation and Collective Action : Archaeological Perspectives*, D. M. Carballo, Ed. (University Press of Colorado, 2013), pp. 35–56.

62. G. A. Johnson, “Organizational structure and scalar stress,” in *Theory and Explanation in Archaeology: The Southampton Conference*, C. Renfrew, M. J. Rowlands, B. A. Segraves, Eds. (Academic Press, 1982), pp. 87–112.

63. G. M. Feinman, L. M. Nicholas, “Compact versus dispersed settlement in pre-Hispanic Mesoamerica,” in *The Neighborhood as a Social and Spatial Unit in Mesoamerican Cities*, M. C. Arnauld, L. R. Manzanilla, M. E. Smith, Eds. (University of Arizona Press, 2012), pp. 132–155.

64. G. M. Feinman, D. M. Carballo, L. M. Nicholas, S. A. Kowalewski, Sustainability and duration of early central places in prehispanic Mesoamerica. *Front. Ecol. Evol.* **11**, (2023).

65. G. M. Feinman, D. M. Carballo, Communication, computation, and governance: A multiscalar vantage on the prehispanic Mesoamerican World. *J. Soc. Comput.* **3**, 91–118 (2022).

66. G. M. Feinman, R. E. Blanton, L. M. Nicholas, S. A. Kowalewski, Reframing the foundation of Monte Albán. *Archaeology* **5**, 155–175 (2022).

67. K. A. Crawford, A. C. Huster, M. A. Peeples, N. Gauthier, M. E. Smith, J. Lobo, A. M. York, D. Lawrence, A systematic approach for studying the persistence of settlements in the past. *Antiquity* **97**, 213–230 (2023).

68. M. E. Smith, J. Lobo, M. A. Peeples, A. M. York, B. W. Stanley, K. A. Crawford, N. Gauthier, A. C. Huster, The persistence of ancient settlements and urban sustainability. *Proc. Natl. Acad. Sci. U.S.A.* **118**, e2018155118 (2021).

69. P. J. Reimer, W. E. N. Austin, E. Bard, A. Bayliss, P. G. Blackwell, C. Bronk Ramsey, M. Butzin, H. Cheng, R. L. Edwards, M. Friedrich, P. M. Grootes, T. P. Guilderson, I. Hajdas, T. J. Heaton, A. G. Hogg, K. A. Hughen, B. Kromer, S. W. Manning, R. Muscheler, J. G.

Palmer, C. Pearson, J. van der Plicht, R. W. Reimer, D. A. Richards, E. M. Scott, J. R. Southon, C. S. M. Turney, L. Wacker, F. Adolphi, U. Büntgen, M. Capano, S. M. Fahrni, A. Fogtmann-Schulz, R. Friedrich, P. Köhler, S. Kudsk, F. Miyake, J. Olsen, F. Reinig, M. Sakamoto, A. Sookdeo, S. Talamo, The Intcal20 Northern Hemisphere radiocarbon age calibration curve (0–55 CAL kBP). *Radiocarbon* **62**, 725–757 (2020).

70. C. Bronk Ramsey, Bayesian analysis of radiocarbon dates. *Radiocarbon* **51**, 337–360 (2009).

71. M. A. Adler, R. H. Wilshusen, Large-scale integrative facilities in tribal societies: Cross-cultural and Southwestern US examples. *Wld. Archaeol.* **22**, 133–146 (1990).

72. M. Dietler, I. Herbich, "Feasts and labor mobilization: Dissecting a fundamental economic practice," in *Feasts: Archaeological and Ethnographic Perspectives*, M. Dietler, B. Hayden, Eds. (Smithsonian Institution Press, 2001), pp. 240–264.

73. C. Fowler, Personhood and social relations in the British Neolithic with a study from the Isle of Man. *J. Mater.l Cult.* **6**, 137–163 (2001).

74. J. Thomas, *Understanding the Neolithic* (Routledge, ed. 2, 1999).

75. T. L. Kienlin, *Bronze Age Tell Communities in Context: An Exploration into Culture, Society, and the Study of European Prehistory. Part 2: Practice, the Social, Space and Materiality* (Archaeopress Archaeology, 2020).

76. W. A. Parkinson, P. R. Duffy, Fortifications and enclosures in European prehistory: A cross-cultural perspective. *J. Archaeol. Res.* **15**, 97–141 (2007).

77. L. Klassen, *Along the Road: Aspects of Causewayed Enclosures in South Scandinavia and Beyond* (Aarhus Univ. Press, 2014).

78. G. L. Miller, Ritual, labor mobilization, and monumental construction in small-scale societies. *Curr. Anthrop.* **62**, 164–197 (2021).

79. M. Furholt, Settlement layout and social organisation in the earliest European Neolithic. *Antiquity* **90**, 1196-1212 (2016).

80. P. Bourdieu, R. Nice, *Outline of a Theory of Practice* (Cambridge Univ. Press, 2013).

81. P. Bourdieu, *The Logic of Practice* (Polity Press, 1990).

82. A. Giddens, *The Constitution of Society: Outline of the Theory of Structuration* (Polity Press, 1984).

83. B. Hillier, J. Hanson, *The Social Logic of Space* (Cambridge Univ. Press, 2009).

84. J. Lobo, L. M. Bettencourt, M. E. Smith, S. Ortman, Settlement scaling theory: Bridging the study of ancient and contemporary urban systems. *Urban Stud.* **57**, 731–747 (2020).

85. G. M. Feinman, G. Cervantes Quequezana, A. Green, D. Lawrence, J. Munson, S. Ortman, C. Petrie, A. Thompson, L. M. Nicholas, Assessing grand narratives of economic inequality across time. *Proc. Natl. Acad. Sci. U.S.A.* **122**, e2400698121 (2025).

86. M. D. McCoy, J. Birch, S. Chirikure, P. Cruz, A. S. Green, D. Gronenborn, D. Lawrence, P. Roscoe, War both reduced and increased inequality over the past ten thousand years. *Proc. Natl. Acad. Sci. U.S.A.* **122**, e2400695121 (2025).

87. M. Porčić, Evaluating social complexity and inequality in the Balkans between 6500 and 4200 BC. *J. Archaeol. Res.* **27**, 335–390 (2019).

88. K. Hegedűs, J. Makkay, "Vésztő-Mágor: A settlement of the Tisza culture," in *The Late Neolithic of the Tisza Region: A Survey of Recent Excavations and Their Findings,* L. Tálas, P. Raczky, Eds. (Szolnok County Museums, 1987), pp. 85–103.

89. C.-M. Lazarovici, G. Lazarovici, *Neoliticul. Vol. 1. Architectura neoliticului şi epocii Cuprului din Romania*, Bibliotheca Archaeologica Moldaviae 4 (Trinitas, 2004).

90. L. Scholtus, J. Müller, European settlement demography. *Doc. Praehistor.* **50**, 22–34 (2023).

91. J. Müller, N. Müller-Scheeßel, I. Cheben, M. Wunderlich, M. Furholt, "On the demographic development of the LBK and Želiezovce settlement sites of Vráble and the Upper Žitava Valley," in *Archaeology in the Žitava Valley I: The LBK and Želiezovce Settlement Site of*

Vráble, M. Furholt, I. Cheben, J. Müller, A. Bistáková, M. Wunderlich, N. Müller-Scheeßel, Eds. (Sidestone Press, 2020), pp. 493–502.

92. P. R. Duffy, River networks and funerary metal in the Bronze Age of the Carpathian Basin. *PLOS ONE* **15**, e0238526 (2020).

93. G. Parditka, P. R. Duffy, From the ashes of Bronze Age fires: A framework for comparison across body treatments. *J. Anthrop. Archaeol.* **71**, 101525 (2023).

94. J. M. O'Shea, A. Nicodemus, ". . . the nearest run thing . . ." The genesis and collapse of a Bronze Age polity in the Maros Valley of Southeastern Europe," in *Coming Together: Comparative Approaches to Popultion Aggregation and Early Urbanization*, A. Gyucha, Ed. (State University of New York Press, 2019), pp. 61–80.

95. P. R. Duffy, Site size hierarchy in middle-range societies. *J. Anthrop. Archaeol.* **37**, 85–99 (2015).

96. K. Kanne, Riding, ruling, and resistance. *Curr. Anthrop.* **63**, 289–329 (2022).

97. N. Ialongo, G. Lago, Consumption patterns in prehistoric Europe are consistent with modern economic behaviour. *Nat. Hum.Behav.* **8**, 1660–1675 (2024).

98. T. Polányi, The rise of idiôtês: Micro-politics of death and community reproduction in Bronze Age Hungary. *J. Anthrop. Archaeol.* **68**, 101445 (2022).

99. M. B. Mulder, I. Fazzio, W. Irons, R. L. McElreath, S. Bowles, A. Bell, T. Hertz, L. Hazzah, Pastoralism and wealth inequality: Revisiting an old question. *Curr. Anthrop.* **51**, 35–48 (2010).

100. V. Isaakidou, P. Halstead, E. Stroud, A. Sarpaki, E. Hatzaki, E. Nitsch, A. M. Y. Bogaard, Changing land use and political economy at Neolithic and Bronze Age Knossos, Crete: Stable carbon ($\delta^{13}C$) and nitrogen ($\delta^{15}N$) isotope analysis of charred crop grains and faunal bone collagen. *Proc. Prehist. Soc.* **88**, 155–191 (2022).

101. A. Bogaard, P. Cruz, M. Fochesato, J. Birch, G. Cervantes Quequezana, S. Chirikure, E. R. Crema, G. M. Feinman, A. S. Green, H. Hamerow, G. Jin, T. Kerig, D. Lawrence, M. D. McCoy, J. Munson, S. G. Ortman, C. A. Petrie, P. Roscoe, Labor, land, and the global dynamics of economic inequality. *Proc. Natl. Acad. Sci. U.S.A.* **122**, e2400694122 (2025).

102. A. K. Styring, C. U. Carmona, V. Isaakidou, A. Karathanou, G. K. Nicholls, A. Sarpaki, A. Bogaard, Urban form and scale shaped the agroecology of early 'cities' in northern Mesopotamia, the Aegean and Central Europe. *J. Agrar.Chang.* **22**, 831–854 (2022).

103. T. Kerig, E. R. Crema, J. Birch, G. M. Feinman, A. S. Green, D. Gronenborn, D. Lawrence, C. A. Petrie, P. Roscoe, A. E. Thompson, T. A. Kohler, 100 generations of wealth equality after the Neolithic transitions. *Proc. Natl. Acad. Sci. U.S.A.* **122**, e2400697122 (2025).

104. R. Risch, H. Meller, Change and continuity in Europe and the Mediterranean around 1600 BC. *Proc. Prehist. Soc.* **81**, 239–264 (2015).

105. K. Kristiansen, T. Earle, "Modelling modes of production: European 3rd and 2nd millennium BC economies," in *Ancient Economies in Comparative Perspective: Material Life, Institutions and Economic Thought*, M. Frangipane, M. Poettinger, B. Schefold, Eds. (Springer International Publishing, 2022), pp. 131–163.

106. E. R. Crema, Statistical inference of prehistoric demography from frequency distributions of radiocarbon dates: A Review and a guide for the perplexed. *J. Archaeol. Method Theory* **29**, 1387–1418 (2022).

107. A. K. Styring, M. Charles, F. Fantone, M. M. Hald, A. McMahon, R. H. Meadow, G. K. Nicholls, A. K. Patel, M. C. Pitre, A. Smith, A. Soltysiak, G. Stein, J. A. Weber, H. Weiss, A. Bogaard, Isotope evidence for agricultural extensification reveals how the world's first cities were fed. *Nat Plants* **3**, 17076 (2017).

108. J. Todorović, A. Cermanović, *Banjica. Naselje Vinčanske Kulture = Banjica: Siedlung der Vinča-Gruppe* (Muzej Grada, 1961).

109. J. Vladár, J. Lichardus, Erforschung der frühäneolithischen Siedlungen in Branč. *Slov. Archeol.* **16**, 263–352 (1968).

110. G. Lazarovici, F. Draşovean, Z. Maxim, *Parţa: Monografie Arheologică Vol. I.1* (Waldpress, 2001).

111. B. Molloy, D. Jovanović, C. Bruyère, M. Marić, J. Bulatović, P. Mertl, C. Horn, L. Milašinović, N. Mirković-Marić, A new Bronze Age mega-fort in southeastern Europe: Recent archaeological investigations at Gradište Iđoš and their regional significance. *J. Field Archaeol.* **45**, 293–314 (2020).

112. S. Perić, V. Miletić, "Geophysical Surveys at Drenovac in 2012 and 2013," in *The Neolithic in the Middle Morava Valley. Interdisciplinary Contributions to Research and Preservation of Archaeological Heritage,* S. Perić, Ed. (Biograf Comp, 2019), pp. 29–45.

113. T. C. Stocker, I. Steinke, *Statistik: Grundlagen und Methodik* (De Gruyter Oldenbourg, ed. 2, 2022).

114. C. Brell, J. Brell, S. Kirsch, *Statistik von Null auf Hundert: Mit Kochrezepten schnell zum Statistik-Grundwissen* (Springer Spektrum, ed. 2, 2017).

115. J. Marzian, "Measuring wealth inequality in pre-Christian Europe using burial mounds data," thesis, Christian-Albrechts-Universität zu Kiel (2021).

116. J. Marzian, J. Laabs, J. Müller, T. Requate, Inequality in relational wealth within the upper societal segment: Evidence from prehistoric Central Europe. *Humanit. Soc. Sci. Commun.* **11**, 557 (2024).

117. A. Harding, "Cornești-Iarcuri and the rise of mega-forts in Bronze Age Europe," in *Fortifications: The Rise and Fall of Defended Sites in Late Bronze and Early Iron Age of South-East Europe,* B. S. Heeb, A. Szentmiklosi, R. d. Krause, M. Wemhoff, Eds. (Staatliche Museen zu Berlin, 2017), pp. 9–14.

118. R. Lehmphul, B. S. Heeb, A. Szentmiklosi, A. Stobbe, R. Krause, "The genesis of the Fortification of Corneşti-larcuri near the Mureş lower course (Romanian Banat) – A phase

model on the chronology of the settlement and fortification structures," in *Bronze Age Fortresses in Europe*, S. Hansen, R. d. Krause, Eds. (Habelt-Verlag, 2019), pp. 253–278.

119. A. Szécsényi-Nagy, G. Brandt, W. Haak, V. Keerl, J. Jakucs, S. Möller-Rieker, K. Köhler, B. G. Mende, K. Oross, T. Marton, A. Osztás, V. Kiss, M. Fecher, G. Pálfi, E. Molnár, K. Sebők, A. Czene, T. Paluch, M. Šlaus, M. Novak, N. Pećina-Šlaus, B. Ősz, V. Voicsek, K. Somogyi, G. Tóth, B. Kromer, E. Bánffy, K. W. Alt, Tracing the genetic origin of Europe's first farmers reveals insights into their social organization. *Proc. Biol. Sci.* **282**, 20150339 (2015).

120. K. Oross, *The Middle Neolithic Settlement Structure of the Site at Balatonszárszó–Kis-erdei-dűlő in a Central European Context* (Archaeopress Archaeology, 2013).

121. A. Osztás, E. Bánffy, I. Zalai-Gaál, K. Oross, T. Marton, K. Somogyi, Alsónyék-Bátaszék: Introduction to a major Neolithic settlement complex in south-east Transdanubia, Hungary. *Bericht RGK* **94**, 7–21 (2016).

122. E. Bánffy, K. Oross, A. Osztás, I. Zalai-Gaál, T. Marton, É. Á. Nyerges, K. Köhler, A. Bayliss, D. Hamilton, A. Whittle, The Alsónyék story: Towards the history of a persistent place. *Bericht RGK* **94**, 283–318 (2016).

123. J. Jakucs, E. Bánffy, K. Oross, V. Voicsek, C. Bronk Ramsey, E. Dunbar, B. Kromer, A. Bayliss, D. Hofmann, P. Marshall, A. Whittle, Between the Vinča and Linearbandkeramik worlds: The diversity of practices and identities in the 54th–53rd centuries cal BC in Southwest Hungary and beyond. *J. World Prehist.* **29**, 267–336 (2016).

124. K. Oross, L. J. E. Cramp, G. Gortva, J. Jakucs, K. Lyublyanovics, T. Marton, G. Serlegi, B. Vágvölgyi, A. Whittle, 'It's still the same old story': The current southern Transdanubian approach to the Neolithisation process of central Europe. *Quat. Int.* **560-561**, 154–178 (2020).

125. M. Marić, M. Radivojević, B. Roberts, D. C. Orton, "Chapter 37: Relative and absolute chronology of Belovode and Pločnik," in *The Rise of Metallurgy in Eurasia: Evolution,*

Organisation and Consumption of Early Metal in the Balkans, M. Radivojević, B. Roberts, M. Marić, J. Kuzmanović Cvetković, T. Rehren, Eds. (Archaeopress, 2021), pp. 439–454.

126. P. Stadler, N. S. Kotova, *Early neolithic settlement Brunn am Gebirge, Wolfholz, site 3 in Lower Austria and the Milanovce phase of the Linear Pottery Culture (LPC)*, Beiträge zur Ur- und Frühgeschichte Mitteleuropas (Beier & Beran, Archäologische Fachliteratur, 2021), pp. 782.

127. V. Szeverényi, P. Czukor, A. Priskin, C. Szalontai, Csanádpalota-Földvár: A Late Bronze Age 'mega-fort' in Southeastern Hungary. *Antaeus* **38**, 213–250 (2022).

128. N. Tasić, "Comparative C-14 dates for the Neolithic settlements in Serbia," in *Vinča and Its World,* D. Srejović, N. Tasić, Eds. (Belgrade, 1988), pp. 45–47.

129. A. McPherron, D. Srejović, Eds. *Divostin and the Neolithic of Central Serbia: Divostin i Neolit Centralne Srbije*, (Dept. of Anthropology, University of Pittsburgh, 1988).

130. D. Borić, "Absolute dating of metallurgical innovations in the Vinča culture of the Balkans," in *Metals and Societies,* T. L. Kienlin, Ed., Universitätsforschungen zur prähistorischen Archäologie (Habelt, 2009), pp. 191–245.

131. M. Porčić, T. Blagojević, J. Pendić, S. Stefanović, The Neolithic demographic transition in the Central Balkans: Population dynamics reconstruction based on new radiocarbon evidence. *Philos. Trans. R. Soc. Lond. B Biol. Sci.* **376**, 20190712 (2021).

132. M. Roeder, 14c-daten und archäologischer befund am beispiel eines hauses von Feudvar bei Mosorin in der Vojvodina. *Germania* **70**, 259–277 (1991).

133. J. Görsdorf, Interpretation der 14C—Datierungen im Berliner labor an materialien eines hauses von Feudvar bei Mosorin in der Vojvodina. *Germania* **70**, 279 (1991).

134. L. Domboróczki, "Settlement structures of the Alföld Linear Pottery Culture (ALPC) in Heves county (North-eastern Hungary): Development models and historical reconstructions on micro, meso and macro levels," in *Interactions Between Different Models of*

Neolithization North of the Central European Agro-Ecological Barrier, Prace Komisji Prehistorii Karpat (Polska Akademia Umiejętności, 2009), vol. 5, pp. 75–127.

135. P. Raczky, Z. Siklósi, Reconsideration of the Copper Age chronology of the eastern Carpathian Basin: A Bayesian approach. *Antiquity* **87**, 555–573 (2013).

136. V. Kiss, M. Csányi, J. Dani, K. P. Fischl, G. Kulcsár, I. Szathmári, Chronology of the Early and Middle Bronze Age in Hungary. New result. *Studia Hercyni* **2**, 173–197 (2019).

137. D. Borić, "Absolute dating of metallurgical innovations in the Vinča culture of the Balkans," in *Metals and Societies: Studies in Honour of Barbara*, S. Ottaway, T. L. Kienlin, B. Roberts, Eds., Universitatsforschungen zur prahistorischen Archaeologie (Habelt, 2009), vol. 169, pp. 191–245.

138. D. Orton, Herding, settlement, and chronology in the Balkan Neolithic. *Eur. J. Archaeol.* **15**, 5–40 (2012).

139. R. W. Yerkes, A. Gyucha, W. A. Parkinson, A multiscalar approach to modeling the end of the Neolithic on the Great Hungarian Plain using calibrated radiocarbon dates. *Radiocarbon* **51**, 1071–1109 (2009).

140. S. Gulyás, P. Sümegi, M. Molnár, New radiocarbon dates from the Late Neolithic tell settlement of Hódmezővásárhely-Gorzsa, SE Hungary. *Radiocarbon* **52**, 1458–1464 (2010).

141. A. Bayliss, B. Gydarska, A. Whittle, F. Draşovean, W. Schier, "Chronological modelling," in *Uivar "Gomilă": A Prehistoric Settlement in the Romanian Banat. Vol. I; Site, Architecture, Stratigraphy and Dating,* F. Draşovean, W. Schier, Eds. (Leidorf, 2020), pp. 509–535.

142. E. Hertelendi, F. Horváth, Radiocarbon chronology of Late Neolithic settlements in the Tisza-Maros Region, Hungary. *Radiocarbon* **34**, 859–866 (1992).

143. M. Jaeger, G. Kulcsár, N. Taylor, R. Staniuk, *Kakucs-Turján: A Middle Bronze Age Multi-layered Fortified Settlement in Central Hungary* (Dr. Rudolf Habelt GmbH, 2018).

144. R. Staniuk, *Kakucs-Turján: Tradition and Practice: Study on Pottery, Chronology and Social Dynamic of the Hungarian Bronze Age*, Studien zur Archäologie in Ostmitteleuropa (Dr. Rudolf Habelt GmbH, 2020).

145. D. Borić, Lepenski Vir chronology and stratigraphy revisited. *Starinar* **49**, 9–60 (2019).

146. P. Stadler, "Auswertung der 14C-daten von Mold mittels kombinationskalibrationen und sequencing der durch seriation ermittelten hausabfolge," in *Die bandkeramische Siedlung von Mold bei Horn in Niederösterreich: Teil 1 - Naturwissenschaftliche Beiträge und Einzelanalysen,* E. Lenneis, Ed., Internationale Archäologie (Verlag Marie Leidorf GmbH, 2010), vol. 115, pp. 23–31.

147. E. Hertelendi, E. Svingor, P. Raczky, F. Horváth, I. Futó, L. Bartosiewicz, Duration of tell settlements at four prehistoric sites in Hungary. *Radiocarbon* **40**, 659–665 (1997).

148. P. Raczky, A. Anders, Régészeti kutatások egy késő neolitikus településen - Polgár-Bosnyákdomb: Előzetes jelentés. *Archaeol. Ert.* **134**, 5–21 (2009).

149. P. Raczky, A. Füzesi, Öcsöd-Kováshalom. *A retrospective look at the interpretations of a Late Neolithic site. Dissertationes Archaeologicae ex Instituto Archaeologico Universitatis de Rolando Eötvös nominatae ser.* **3**, 9–42 (2017).

150. A. Füzesi, I. Hohle, N. Faragó, K. Rassmann, E. Bánffy, P. Raczky, On the 'pseudo-ditch' system of the Late Neolithic Öcsöd-Kováshalom settlement complex on the Great Hungarian Plain. *Doc. Praeh.* **50**, 82–109 (2023).

151. P. Biagi, M. Spataro, "New observations on the radiocarbon chronology of the Starčevo-Criş and Körös cultures," in *Prehistoric Archaeology and Anthropological Theory and Education*, L. Nikolova, J. Fritz, J. Higgins, Eds. (RPRP, 2005), pp. 35–40.

152. J. M. O'Shea, A. W. Barker, A. Nicodemus, S. Sherwood, A. Szentmiklosi, Archaeological investigations at Pecica "Şanţul Mare": The 2006 campaign. *Analele Banatului (Serie nouă) Arheologie - Istorie* **14**, 211–228 (2006).

153. J. M. O'Shea, A. W. Barker, S. Sherwood, A. Szentmiklosi, New archaeological investigations at Pecica-Şanţul Mare. *Analele Banatului (Serie nouă) Arheologie - Istorie* **12–13**, 81–110 (2005).

154. P. Raczky, A. Anders, Polgár-Bosnyákdomb, a Late Neolithic tell-like settlement on Polgár Island (NE Hungary). Preliminary results of the investigations. *Folia Quaternaria,* Comenius University in Bratislava, **84**, 99–112 (2016).

155. P. Raczky, A. Anders, "The times they are a-changin': Revisiting the chronological framework of the Late Neolithic settlement complex at Polgár–Csőszhalom," in *PANTA RHEI. Studies in Chronology and Cultural Development of the South-Eastern and Central Europe in Earlier Prehistory Presented to J. Pavúk on the Occasion of his 75th Birthday,* P. Kalábková, B. Kovár, P. Pavúk, J. Šuteková, Eds., Studia Archaeologica et Mediaevalia 11 (Comenius Univ. in Bratislava, 2010), pp. 357–378.

156. M. Črešnar, Poskus Določitve kulturne skupine Kisapostag v vzhodni Slovenji. *Zbornik soboøkega muzeja* **15**, 107–134 (2010).

157. F. Gogâltan, V. Sava, R. d. Krause, "Sântana-Cetatea Veche. A Late Bronze Age mega-fort in the Lower Mureș Basin in southwestern Romania," in *Bronze Age Fortresses in Europe*, S. Hansen, R. d. Krause, Eds. (Habelt-Verlag, 2019), pp. 191–221.

158. J. Jakucs, LBK and Vinča in South-East Transdanubia: Comments on merging, interleaving and diversity. *Quat. Int.* **560-561**, 119–141 (2020).

159. N. Kalicz, P. Raczky, "The Late Neolithic of the Tisza region: A survey of recent archaeological research," in *The Late Neolithic of the Tisza Region: A Survey of Recent Excavations and Their Findings,* L. Tálas, P. Raczky, Eds. (Szolnok County Museums, 1987), pp. 11–30.

160. K. Oross, J. Jakucs, T. Marton, E. Gál, A. Whittle, "Pioneers, carpenters, outsiders: Radiocarbon dating of early farmers in western Hungary," in *Relatively Absolute*, M. Marić, J. Bulatović, N. Marković, Eds. (Belgrade, 2023), pp. 135–158.

161. T.-T. Daróczi, M. Csányi, J. Tárnoki, F. Nagy, J. Olsen, Túrkeve-Terehalom in the Eastern Carpathian Basin. Bronze Age multi-stratified site provides high-precision chronology with continental implications. *Praehist. Z.* **98**, 136–190 (2023).

162. F. Draşovean, W. Schier, *Uivar "Gomilă":A prehistoric settlement in the Romanian Banat. Vol. I; Site, Architecture, Stratigraphy and Dating* (Leidorf, 2020).

163. J. Jakucs, K. Oross, E. Bánffy, V. Voicsek, E. Dunbar, P. Reimer, A. Bayliss, P. Marshall, A. Whittle, Rows with the neighbours: The short lives of longhouses at the Neolithic site of Versend-Gilencsa. *Antiquity* **92**, 91–117 (2018).

164. A. Gyucha, R. W. Yerkes, W. A. Parkinson, "Chapter 8: Settlement chronology and layout," in *Bikeri: Two Copper Age villages on the Great Hungarian Plain*, W. A. Parkinson, A. Gyucha, R. W. Yerkes, Eds. (Cotsen Institute Press, 2021), pp. 163–186.

165. M. Krznarić-Škrivanko, The results of Dimitrijević's excavations at Sopot in light of recent research. *OpArch.* **37**, 371–395 (2014).

166. R. Staniuk, M. Furholt, N. Müller-Scheeßel, I. Cheben, "The archaeological features from the LBK and Želiezovce settlement site of Vráble," in *Archaeology in the Žitava Valley I: The LBK and Želiezovce Settlement Site of Vráble*, M. Furholt, I. Cheben, J. Müller, A. Bistáková, M. Wunderlich, N. Müller-Scheeßel, Eds., Scales of Transformation (Sidestone Press, 2020), pp. 95–158.

167. T. Horváth, M. Kozák, Á. Pető, The complex investigations of the stone artefacts from Vatya-earthworks of Fejér county. Part *I. Alba. Regia* **XXX 7-37**, (2001).

168. K. Oross, Das neolitische dorf von Balatonszarszo (Forschungen zwischen 2000-2002). *Antaeus* **27**, 61–80 (2004).

169. T. L. Kienlin, K. P. Fischl, T. Pusztai, "III. Catalogue," in *Borsod Region Bronze Age Settlement (BORBAS) Catalogue of the Early to Middle Bronze Age Tell Sites Covered by Magnetometry and Surface Survey*, T. L. Kienlin, K. P. Fischl, T. Pusztai, Eds. (Dr. Rudolf Habel GmbH, 2018).

170. I. Németi, Descoperiri arheologice din hotarul orașului Carei (Jud. Satu Mare). *Satu Mare - Studii şi Comunicări* **1981–1982**, 179 (1982).

171. T. L. Kienlin, "Diversity rather than uniformity: Bronze Age tell settlements in North-Western Romania. Part II. Catalogue of sites covered by magnetometry in Bihor and Satu Mare Counties," in *Bronze Age Tell Settlements in North-Western Romania,* T. L. Kienlin, A. Găvan, Eds. (Dr. Rudolf Habelt GmbH, 2021), pp. 79–216.

172. F. Falkenstein, B. Hänsel, P. Medović, "Feudvar near Mošorin (Serbia) – Excavations and research in a micro-region at the confluence of the Danube and Tisza: A recapitulation after thirty years," in *Feudvar III: Die Archäobotanik*, H. Kroll, F. Falkenstein, K. Reed, Eds. (Würzburg Univ. Press, 2016), pp. 5–35.

173. F. Falkenstein, B. Hänsel, *Die Siedlungsgeschichte des Titeler Plateaus*, Prähistorische Archäologie in Südosteuropa (Oetker/Voges, 1998).

174. V. Á. S. Kállay, Die kupferzeitliche Ringanlage von Füzesabony. *Jschr. Mitteldt. Vorgesch.* **73**, 125–130 (1990).

175. R. Šošić Klindžić, B. Šiljeg, H. Kalafatić, Multiscale and multitemporal remote sensing for Neolithic settlement detection and protection—The case of Gorjani, Croatia. *Remote Sens* **16**, 736 (2024).

176. P. Raczky, I. Fodor, Z. Mester, Régészeti kutatások Hajdúböszörmény-Pródi-halom. *Archaeol. Ert.* **135**, 161–182 (2010).

177. F. Horváth, "Late Neolithic ditches, fortifications and tells in the Hungarian Tisza-region," in *Gomolava - Chronologie und Stratigraphie der vorgeschichtlichen und antiken Kulturen de Donauniederung und Südosteuropas,* N. Tasić, J. Petrović, Eds. (Institut za Izucavanje Istorije Vojvodine i Savez Arheoloskih Drustava Jugoslavije, Novi Sad, 1988), pp. 145–149.

178. P. Raczky, A. Fűzesi, K. Rassmann, H. Höler-Brockmann, M. Podgorelec, E. Bánffy, "Neolithic settlement mounds in the southern Alföld (previous research and new investigations at Hódmezővásárhely-Kökénydomb," in *From Farmers to Heroes?*

Archaeological Studies in Honor of Sławomir Kadrow, M. Dębiec, J. Górski, J. Müller, M. Nowak, A. Pelisiak, T. Saile, P. Włodarczak, Eds., Universitätsforschungen zur Prähistorischen Archäologie (Verlag Dr. Rudolf Habelt GmbH, 2022), pp. 255–277.

179..P ető, G. Serlegi, J. Niebieszczański, M. Molnár, M. Jaeger, G. Kulcsár, N. Taylor, "Report on the geoarchaeological survey of Kakucs-Turján site," in *Kakucs-Turján: A Middle Bronze Age Multi-layered Fortified Settlement in Central Hungary*, M. Jaeger, G. Kulcsár, N. Taylor, R. Staniuk, Eds. (Dr. Rudolf Habelt GmbH, 2018), pp. 25–40.

180. J. Vladár, *Pohrebiská zo staršej doby bronzovej v Branči: Gräberfelder aus der älteren Bronzezeit in Branč* (Vydavatel'stvo Slovenskej Akadémie Vied, 1973), pp. 267.

181. V. Furmánek, L. Veliačik, J. Vladár, *Die Bronzezeit im Slowakischen Raum*, Prahistorische archaologie in sudosteuropa (Verlag Marie Leidorf GmbH, 1999).

182. V. Szeverényi, P. Czukor, A. Priskin, C. Szalontai, "Recent work on Late Bronze Age fortified settlements in south-east Hungary," in *Fortifications: The Rise And Fall Of Defended Sites In Late Bronze And Early Iron Age Of South-East Europe,* B. S. Heeb, A. Szentmiklosi, R. Krause, M. Wemhoff, Eds. (Staatliche Museen zu Berlin, 2017), pp. 135–148.

183. I. Poroszlai, "Nagykőrös-Földvár," in *Le Bel Âge du Bronze en Hongrie,* I. Bóna, P. Raczky, Eds. (Centre Européen d'Archéologie du Mont-Beuvray, 1994), pp. 156–158.

184. A. Točik, *Nitriansky Hrádok-Zámeček: Bronzezeitliche befestigte Ansiedlung der Mađarovce-Kultur 2* (Archeologicý Ústav SAV, 1981).

185. L. Olexa, *Nižna Myšľa. Osada a pohrebisko z doby bronzovej* (Archeologické pamätníky Slovenska, 2003).

186. M. Jaeger, *Bronze Age Fortified Settlements in Central Europe*, Studien zur Archäologie in Ostmitteleuropa (Dr. Rudolf Habelt GmbH, 2016).

187. J. Banner, A hódmezővásárhelyi Nagytatársánc. *Dolgozatok* **15**, 93–114 (1939).

188. I. Ordentlich, M. Lie, C. Ghemiș, "Otomani "Cetatea de pămant = Földvár", Bihor County," in *Bronze Age Tell, Tell-Like and Mound-Like Settlements on the Eastern Frontier of the Carpathian Basin: History of Research*, F. Gogâltan, C. Cordoş, A. Ignat, Eds. (Editura Mega, 2014), pp. 129–138.

189. I. Ordentlich, Probleme der Befestigungsanlagen in der Siedlungen der Otomanikultur in deren rumanischem Verbreitungs-gebiet. *Dacia* **13**, 457–474 (1969).

190. F. Gogâltan, "Fortified Bronze Age tell settlements in the Carpathian Basin. A general overview," in *Defensive Structures from Central Europe to the Aegean in the 3rd and 2nd Millennia BC,* J. Czebresuk, S. Kadrow, J. Müller, Eds. (SAO, 2008), pp. 39–56.

191. A. Nicodemus, "Bronze Age economies of the Carpathian Basin: Trade, craft production, and agro-pastoral intensification," thesis, University of Michigan, Ann Arbor, MI (2014).

192. Z. Székely, Aşezările şi necropola culturii Otomani de la Pir (Judeţul Satue Mare) (the settlements and the cemetery of the Otomani culture from Pir [Satu Mare county]). *Thraco-Dacica* **21**, 103–146 (2000).

193. L. Marta, "Pișcolt-Ógát, Satu Mare County," in *Bronze Age Tell, Tell-Like and Mound-Like Settlements on the Eastern Frontier of the Carpathian Basin: History of Research*, F. Gogâltan, C. Cordoş, A. Ignat, Eds. (Editura Mega, 2014), pp. 186–188.

194. P. Raczky, W. Meier-Arendt, A. Anders, Z. Hajdú, E. Nagy, K. Kurucz, L. Domboróczki, K. Sebok, P. Sümegi, E. Magyari, Z. Szántó, S. Gulyás, K. Dobó, E. Bácskay, K. T. Bíró, C. Schwartz, "Polgár-Csőszhalom (1989-2000): Summary of the Hungarian-German excavations on a Neolithic settlement in Eastern Hungary," in *Mauerschau: Festschrift für Manfred Korfmann,* M. Korfmann, R. Aslan, Eds. (Verlag Bernhard, 2002), pp. 833–860.

195. P. Raczky, A. Anders, E. Nagy, K. Kurucz, Z. Hajdú, "Polgár-Nagy Kasziba. Rézkori sírok a Kr. e. V. évezred végéről," in *Utak a Múltba: Az M3-as autópálya régéeszeti leletmentései,* P. Raczky, T. Kovács, A. Anders, Eds. (Magyar Nemzeti Múzeum és az Eötvös Loránd Tudomány egyetem Régészettudományi Intézet, 1997), pp. 47–50.

196. P. Raczky, “Cultural context of the Late Neoltihic site at Polgár-Csőszhalom (Hungary),” in *Karanova Band III: Beiträge zum Neolithikum in Südosteuropa,* S. Hiller, V. Nikolov, Eds. (Phoibos Verlag, 2000), pp. 405–414.

197. M. Jaeger, “Bronze Age defensive settlement in the context of long-range relationships: How far is it from fortified villages to Citadels?,” in *Rebellion and Inequality in Archaeology: Proceedings of the Kiel Workshops “Archaeology of Rebellion” (2014) and “Social Inequality as a Topic in Archaeology” (2015)*, S. Hansen, J. Müller, Eds., Human Development in Landscapes (Dr. Rudolf Habelt GmbH, 2017), pp. 185–204.

198. T. Bader, “Die befestigten bronzezeitlichen Siedlungen in Nordwestrumänien,” in *Beiträge zum bronzezeitlichen Burgenbau in Mitteleuropa,* B. Chropovsky, J. Herrmann, Eds. (Zentralinstitut für Alte Geschichte und Archäologie der Akademie der Wissenschaften der DDR, 1982), pp. 47–70.

199. I. Ordentlich, A. Găvan, C. Ghemiș, “Sălacea Dealul Vida = Vida hegy”, *Bihor County, in Bronze Age Tell, Tell-Like and Mound-Like Settlements on the Eastern Frontier of the Carpathian Basin: History of Research*, F. Gogâltan, C. Cordoş, A. Ignat, Eds. (Editura Mega, 2014), pp. 207–230.

200. M. Rusu, E. Dörmer, I. Ordentlich, “Die erdburg von Sântana-Arad in dem zeitgleichen archäologischen kontext,” in *Transsilvanica: Archäologische Untersuchungen zur älteren Geschichte des südöstischen Mitteleuropa: Gedenkschift für Kurt Horedt,* N. G. O. Boroffka, T. Sorocenau, Eds. (Leidorf, Rahden, 1999), pp. 143–165.

201. A. Endrődi, F. Gyulai, Soroksár - Várhegy: A fortified bronze age settlement in the outskirts of Budapest. *Commun. Archaeol. Hung.* **1999**, 5–34 (1999).

202. J. Vladár, Osteuropäische und mediterrane Einflüsse im Bebiet der Slowakei wahrend der Bronzezeit. *Slov. Arch.* **12**, 253–357 (1973).

203. J. Makkay, M. Sériadès, L’Enclos sacré de Szarvas. *Archéologia* **390**, 56–65 (2002).

204. J. Makkay, *Die Grabenanlagen im indogermanischen Raum* (J. Makkay, 2001).

205. K. P. Fischl, T. L. Kienlin, Results of a systematic survey programme on the Hatvan sites of Emőd-Nagyhalom and Tard-Tatárdomb in northern Hungary. *cta Archaeol. Acad. Sci. Hung.* **64**, 5–32 (2013).

206. I. Bóna, G. Nováki, “Alpár bronzkori és Árpád-kori vára,” in *Cumania, Vol. VII,* A. Horváth, Ed. (Bács-Kiskun Megyei Múzeumok Közleményei, 1982), pp. 17–117.

207. P. Patay, *Kupferzeitliche Siedlung von Tiszalúc*, Inventaria praehistorica Hungariae (Magyar Nemzeti Múzeum, 2005).

208. C. Siklódi, Előzetes jelentés a Tiszaug-Kisrétparti rézkori telep ásatásáról. *Archaeol. Ert.,* Tisza Nyomda Kft. **109**, 231–238 (1982).

209. M. Csányi, J. Tárnoki, “Bronzkori teli-telepek a Közép-Tisza-vidéken,” in *Vendégségben őseinnk háza táján. Állandó régészeti kiállítás a Szolnoki Damjanich János Múzeumban*, L. Madaras, R. Kertész, Z. Polgár, Eds. (Tisza Nyomda Kft, 1996), pp. 31–48.

210. L. Lichtenstein, Z. Rózsa, J. Szigeti, B. Tugya, Chronological observations and the origin of the appearance of red slip coating on Árpád Age cauldron fragments in the light of the excavations at Végegyháza, Zsibrik-domb site. *Acta Archaeol. Acad. Sci. Hung.* **72**, 215–233 (2021).

211. A. Sarris, “Chapter 6: Geophysical remote sensing,” in *Bikeri: Two Early Copper Age Settlements on the Great Hungarian Plain*, W. A. Parkinson, A. Gyucha, R. W. Yerkes, Eds. (UCLA Cotsen Institute of Archaeological Press, 2021), pp. 75–92.

212. R. W. Yerkes, W. A. Parkinson, A. Gyucha, M. Morris Downing, “Chapter 7: Excavation methods and results,” in *Bikeri: Two Early Copper Age Settlements on the Great Hungarian Plain*, W. A. Parkinson, A. Gyucha, R. W. Yerkes, Eds. (UCLA Cotsen Institute of Archaeological Press, 2021), pp. 95–161.

213. A. Sarris, N. Papadopoulos, A. Agapiou, M. C. Salvi, D. G. Hadjimitsis, W. A. Parkinson, R. W. Yerkes, A. Gyucha, P. R. Duffy, Integration of geophysical surveys, ground hyperspectral measurements, aerial and satellite imagery for archaeological prospection of prehistoric

sites: The case study of Vésztő-Mágor Tell, Hungary. *J. Archaeol. Sci.* **40**, 1454–1470 (2013).

214. J. Bátora, B. A, J. Gresky, M. Ivanova, K. Rassmann, P. Tóth, K. Winkelmann, "The rise and decline of the Early Bronze Age settlement Fidvár near Vráble, Nitra," in *Collapse or Continuity? Environment and Development of Bronze Age Human Landscapes*, J. Kneisel, W. Kirleis, M. DalCorso, N. Taylor, T. V., Eds. (Dr. Rudolf Habelt GmbH, 2012), pp. 111–129.
